# Candidate SNP Markers Significantly Altering the Affinity of TATA-Binding Protein for the Promoters of Human Hub Genes for Atherogenesis, Atherosclerosis and Atheroprotection

**DOI:** 10.3390/ijms24109010

**Published:** 2023-05-19

**Authors:** Anton Bogomolov, Sergey Filonov, Irina Chadaeva, Dmitry Rasskazov, Bato Khandaev, Karina Zolotareva, Anna Kazachek, Dmitry Oshchepkov, Vladimir A. Ivanisenko, Pavel Demenkov, Nikolay Podkolodnyy, Ekaterina Kondratyuk, Petr Ponomarenko, Olga Podkolodnaya, Zakhar Mustafin, Ludmila Savinkova, Nikolay Kolchanov, Natalya Tverdokhleb, Mikhail Ponomarenko

**Affiliations:** 1 Institute of Cytology and Genetics, Siberian Branch of Russian Academy of Sciences (SB RAS), Novosibirsk 630090, Russia; mantis_anton@bionet.nsc.ru (A.B.); filonovsv@yandex.ru (S.F.); ichadaeva@bionet.nsc.ru (I.C.); rassk@bionet.nsc.ru (D.R.); b.khandaev@g.nsu.ru (B.K.); ka125699ri@yandex.ru (K.Z.); kazachekav@bionet.nsc.ru (A.K.); diman@bionet.nsc.ru (D.O.); salix@bionet.nsc.ru (V.A.I.); demps@bionet.nsc.ru (P.D.); pnl@bionet.nsc.ru (N.P.); kandy@ngs.ru (E.K.); pon.petr@gmail.com (P.P.); opodkol@bionet.nsc.ru (O.P.); mustafinzs@bionet.nsc.ru (Z.M.); lksav@bionet.nsc.ru (L.S.); nata@bionet.nsc.ru (N.T.); pon@bionet.nsc.ru (M.P.); 2 The Natural Sciences Department, Novosibirsk State University, Novosibirsk 630090, Russia; 3 Institute of Computational Mathematics and Mathematical Geophysics, Novosibirsk 630090, Russia

**Keywords:** human, atherogenesis, atheroprotection, atherosclerosis, hub gene, promoter, TBP, TATA box, SNP, candidate SNP marker, gene expression change, natural selection, in silico verification

## Abstract

Atherosclerosis is a systemic disease in which focal lesions in arteries promote the build-up of lipoproteins and cholesterol they are transporting. The development of atheroma (atherogenesis) narrows blood vessels, reduces the blood supply and leads to cardiovascular diseases. According to the World Health Organization (WHO), cardiovascular diseases are the leading cause of death, which has been especially boosted since the COVID-19 pandemic. There is a variety of contributors to atherosclerosis, including lifestyle factors and genetic predisposition. Antioxidant diets and recreational exercises act as atheroprotectors and can retard atherogenesis. The search for molecular markers of atherogenesis and atheroprotection for predictive, preventive and personalized medicine appears to be the most promising direction for the study of atherosclerosis. In this work, we have analyzed 1068 human genes associated with atherogenesis, atherosclerosis and atheroprotection. The hub genes regulating these processes have been found to be the most ancient. In silico analysis of all 5112 SNPs in their promoters has revealed 330 candidate SNP markers, which statistically significantly change the affinity of the TATA-binding protein (TBP) for these promoters. These molecular markers have made us confident that natural selection acts against underexpression of the hub genes for atherogenesis, atherosclerosis and atheroprotection. At the same time, upregulation of the one for atheroprotection promotes human health.

## 1. Introduction

Atherosclerosis is characterized by deposits made of lipids, pieces of connective tissue and inflammatory and smooth muscle cells inside the arteries [1]. All its stages from atheroma formation and growth to complications (e.g., myocardial infarction [2] and stroke [3] as the leading causes of death, according to the World Health Organization (WHO) [4]) represent the inflammatory response to the lesion caused by specific cytokines. Atherosclerosis is an aging-related disease [5], with such factors as acquired dyslipidemia [6], acquired diabetes [7], tobacco smoking [8], past diseases [9], lack of physical activity and excessive physical activity that are collectively called lifestyle increasing the risk to develop it. However, atheromas in artery walls can be detected pathoanatomically even as early as during embryogenesis [10].

Genetic predisposition plays an extremely important role. The most prevalent and severe form of monogenic hypercholesterolemia (familial hypercholesterolemia, FH), which was first reported in 1973, has become the first genetic lipid metabolism disorder to have been characterized clinically and at the molecular level quite recently [11]. This is an autosomal codominant genetic disease and is caused by mutations in several genes. As is known, the plasma levels of low-density lipoproteins in persons homozygous for FH in China, where foods are low in cholesterol and saturated fat, and in western countries are similar [12]. It therefore appears that there must be a way to control atherogenesis by changing living conditions and lifestyle on the basis of the information about an individual’s predisposition to abnormal developmental patterns, and this information can become known through the sequencing of the individual’s genome [13].

According to the modern view, atherogenesis begins with an accumulation of low-density lipoproteins on the internal vascular wall [14]. Monocytes internalize lipoproteins and differentiate into macrophages [15], which, once dead, become part of the lipid patch [16]. Its further growth into a streak and fibrotic thickening leads to the development of an atheroma with the calcification of blood vessels [17]. Clinical observations describe atherogenesis as a non-monotonous process with rises primarily postprandially [18] and in acute infectious diseases [19]. The latter was illustrated by the consequences of the COVID-19 pandemic described by many authors (e.g., [20]).

Hopefully, changes to lifestyle can be exceptionally beneficial. The Mediterranean fish diet enriched for antioxidants can retard both atherogenesis and atherosclerosis, hence its famed atheroprotective properties [21]. Additionally, 23 formulas of traditional Chinese medicine-based herbal medicines with anti-inflammatory, antioxidant and atheroprotective properties with promise for decreasing the number of plaques and improving lipid metabolism have been patented [22]. Similarly, bilberry has recently been associated with the prevention of atherosclerosis, and this finding gave rise to an intensive study of the molecular bases of the atheroprotective properties that berries possess [23]. In addition to a healthy diet and physical exercises [24], new approaches exist in medicine for the improvement of the rheological properties of the blood: apheresis [25] and activation of low-density lipoproteins’ receptors [26]. Thus, atherogenesis and atheroprotection are intermittent, counteracting processes, and their balance depends equally on lifestyle and genetic predisposition. As far as the genetic predisposition is concerned, the most promising direction for the study of atherosclerosis [27] is through identification of the molecular mechanisms underlying this pathology in particular, and atherogenesis and atheroprotection [28] in general, by searching for molecular markers [29] for predictive, preventive, personalized and participatory (4P) medicine [30].

The most large-scale research effort of the 21st century is the 1000 Genomes Project [31,32]. As a result, the genomes of more than 10,000 individuals have been sequenced [33], and more than 515,000 single nucleotide polymorphisms (SNPs) that make these genomes different from the reference human genome used as their consensus have been revealed [34]. The Ensembl [35] database provides free access to the reference human genome, and the dbSNP [36] database documents human SNPs. The alleles of these SNPs or their combinations (haplogroups), which occur at a significantly higher frequency in patients with certain diseases than in unaffected people, are called the clinical SNP markers of predisposition to these diseases [37].

Because personalized medicine [30] uses SNP markers of diseases for diagnosing these diseases and offering an adequate course of therapy, a comparison of representative cohorts of patients and healthy volunteers, including a test of Hardy–Weinberg equilibrium [38], should necessarily precede any clinical use of such markers. However, cohort studies are so demanding in terms of time, money and labor that it is just impractical to think of using them as a means to check whether or not each of more than 515,000 SNPs is a molecular marker of predisposition to something from among more than 55,000 diseases [39]. At the same time, Haldane’s dilemma [40] and the neutral theory of molecular evolution [41] provide independent evidence that an absolute majority of human SNPs are neutral. Consequently, as far as neutral SNPs are concerned, their much less demanding computer-assisted identification for exclusion from the cohort search for clinical molecular markers can be helpful to personalized medicine [30].

The clinical SNP markers that have been identified to date can be found in the freely accessible databases OMIM [42], ClinVar [43] and GWAS Central [44]. By far the majority of these markers were found in the protein-coding regions of genes in the form of damage to protein molecules, which is easier to detect, because protein damage is subject to no variation in all human tissues. However, protein damage attributable to SNPs cannot be repaired due to an ethical prohibition to edit individual human genomes [45]. Admittedly, research into genome editing with the use of animal models to treat human diseases is one of the hot spots in modern biomedical genetics and pharmacogenomics (see, for example, [46]).

At the same time, only an infinitesimally small share of the known SNP markers of diseases have been identified in the regulatory regions of genes [35,42,43,44], where SNPs only modulate their expression levels in humans, not affecting the proteins encoded by these genes. Because the expression levels of the genes depend on the tissue and the stimulus even without a single SNP in the regulatory regions of these genes, regulatory SNP markers of diseases are more difficult to detect [47].

Nevertheless, the pathogenic effects of such molecular markers can be eliminated by changing lifestyle [48], breaking unhealthy habits [49], using low-molecular inhibitors of proteins encoded by the corresponding genes as medicines against excess of these proteins [50], using exogenous recombinant human proteins to compensate for the lack of these proteins caused by the corresponding SNPs [51] and using short antisense oligonucleotides as pharmacotherapy treatment of SNP-promoted misregulation of human gene expression [52]. That is why regulatory SNP markers are becoming more and more in focus as personalized medicine progresses [53].

Various regulatory biomedical SNP markers were found due to a significant change in the affinity of the TATA-binding protein (TBP) to the promoters of the human genes carrying these SNPs. This change takes place when TBP initiates the assembly of pre-initiation complexes from a dense transcriptionally inactive packaging of these promoters into nucleosomes [54,55], this assembly being one of the earliest stages in the initiation of transcription of these genes [56,57,58,59]. If it were not for this molecular event, no primary initiation of gene transcription would have happened: knockout model animals (TBP-/-) run out of maternal TBPs at the blastula stage and have their development arrested [60,61], while chromatin immunoprecipitation followed by sequencing (ChIP-Seq) locates TBP binding sites before the starts of most transcripts [62,63,64].

Therefore, the SNPs that alter TBP-promoter affinity are phenotypically easy to observe: the higher the affinity of TBP to the promoter carrying that particular SNP, the higher the expression level of the gene regulated by this promoter, which was confirmed by a large number of independent experiments [65,66,67,68,69]. By contrast, the in silico assessment of all other regulatory SNPs’ phenotypic manifestations remains a challenge bioinformatics has to overcome [70,71].

In our previous works, we proposed a bioinformatics model of TBP binding to the promoter in three consecutive steps [72]: (i) TBP slides [73] along the slightly bending DNA helix of the promoter [74] <=>; (ii) TBP stops at the potential TBP binding site [75,76] <=>; and (iii) the DNA helix of the promoter has a bend angle of 90°, so the TBP-promoter [77,78,79] complex becomes fixed. The model was confirmed by an independent in vitro experiment [80]. Nevertheless, we additionally checked this in silico model using 68 independent experimental cases [68] in PubMed [81] in our real-time in vitro experiments [82,83] in equilibrium [84] and nonequilibrium [85,86] conditions, and with transfected human cell cultures ex vivo [87]. This led us to the development of SNP_TATA_Comparator, a freely accessible web service [88]. SNP_TATA_Comparator uses two 90-bp DNA sequences of the promoter before the transcription start site (one corresponding to the norm, the other to the minor variant of the given SNP) as input data and outputs in silico estimates of TBP affinity for these promoter variants expressed as nanomoles per liter (nM) of the equilibrium dissociation constant, Kd, of the TBP-DNA complex, standard errors of these estimates and the statistical significance, p, of the mutation-induced change in the expression level of the gene regulated by this promoter according to Fischer’s Z-test [89]. With this web service, we have previously revealed candidate SNP markers of autoimmune disorders [90], behavioral disorders [91] and chronopathologies [92] Following a 50-year-old tradition to compare the frequencies of different types of mutations (transitions vs. transversions [93], inserts vs. deletions [94] and synonymous vs. nonsynonymous substitutions [95]) in order to estimate the parameters of molecular evolution, we compared the respective frequencies of SNPs that increase and decrease TBP affinity to the promoters of the same genes. This comparison was done in response to the question as to whether these genes are under natural selection or neutral drift [96]. In so doing, we have already named the likely modes of evolution of human genes associated with aggression [97], rheumatoid arthritis [98], hypertension [99] and all protein-coding human Y chromosome genes associated with the male reproductive potential [100]. Finally, based on this standpoint, we have previously used SNP_TATA_Comparator [88] and analyzed all 1189 SNPs in all 90-bp promoters before the transcription start sites of 26 and 8 human genes associated with atherogenesis and atheroprotection, respectively [101,102]. As a result, we have revealed 238 candidate SNP markers that significantly change the affinity of the TATA-binding protein (TBP) for these promoters and are significantly consistent with stabilizing selection as the sum of neutral drift accelerating atherogenesis and natural selection favoring enhanced atheroprotection.

For that reason, here we have used the same way to analyze all human hub genes for atherogenesis, atherosclerosis and atheroprotection and verified the results obtained, assuming the hypothesis about a role of self-domestication in human evolution [103,104].

## 2. Results and Discussion

### 2.1. The Human Hub Genes for Atherogenesis, Atherosclerosis and Atheroprotection

**Figure 1:**
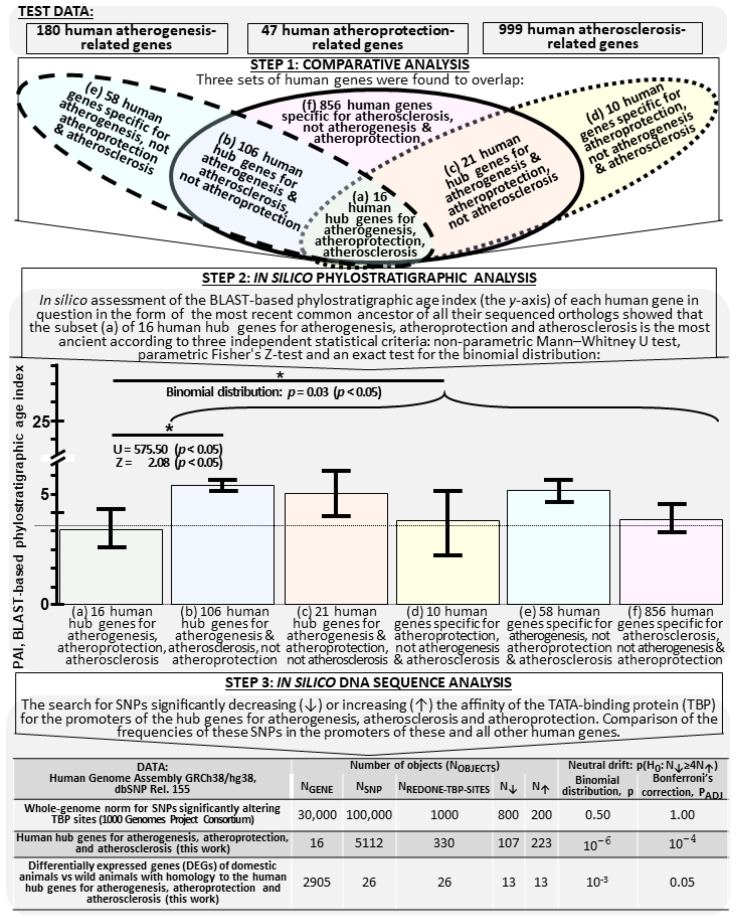
The flowchart depicting the steps in the study of all 1068 human genes associated with atherogenesis, atherosclerosis and atheroprotection according to the NCBI Gene database [105] as accessed on 15 March 2023. Legend: the heights of the gene bars and error bars in the chart in the central part are proportional to the arithmetic mean of the PAIs and the standard error of the mean (SEM), the numerical values whereof are given in Table S1 (hereinafter: see Supplementary Materials); the dotted line is an auxiliary line running above the gray-green bar for the subset (a) of 16 human hub genes for atherogenesis, atherosclerosis and atheroprotection, the tops of the other five bars being above the line. (*): statistical significance *p* < 0.05. U and Z are the values of the statistics in the nonparametric Mann–Whitney U test and parametric Fischer’s Z-test according to STATISTICA (StatSoft^TM^, Tulsa, OK, USA). PAI, genes’ phylostratigraphic age index evaluated against the BLAST-based scale [106] using the freely available web service Orthoscape [107]; BLAST-based PAI scale: 0, Cellular organisms; 1, Eukaryota; 2, Opisthokonta; 3, Metazoa; 4, Eumetazoa; 5, Bilateria; 6, Deuterostomia; 7, Chordata; 8, Craniata; 9, Vertebrata; 10, Gnathostomata; 11, Teleostomi; 12, Euteleostomi; 13, Sarcopterygii; 14, Dipnotetrapodomorpha; 15, Tetrapoda; 16, Amniota; 17, Mammalia; 18, Theria; 19, Eutheria; 20, Euarchontoglires; 21, Primates; 22, Haplorrhini; 23, Simiiformes; 24, Catarrhini; 25, Hominoidea; 26, Hominidae; 27, Homininae; and 28, Homo.

We have studied all 1068 human genes that were associated with atherogenesis (*n* = 180), atherosclerosis (*n* = 999) and atheroprotection (*n* = 47) according to the most current build of NCBI’s Gene database [105] (the workflow is explained in Section 3.1 and depicted [106,107] in Figure 1). Symbols for each of these genes are explained in Table S1 (here and elsewhere: see Supplementary Materials). As the first step, we performed a comparative analysis of the overlap between these three sets of human genes (for the results, see the Venn diagram in the upper part of Figure 1). A total of six non-overlapping subsets have been revealed, their members being presented in Table S1. For example, 16 hub genes for atherogenesis, atherosclerosis and atheroprotection have been found: *APOA1*, *C1QTNF9*, *CD163*, *CRP*, *CXCR4*, *HMOX1*, *KLF2*, *LCAT*, *NFE2L2*, *NR1H3*, *PF4*, *PON1*, *PON2*, *SERPINF1*, *TLR2* and *YAP1* (Figure 1, gray-green bar).

### 2.2. Human Hub Genes for Atherosgenesis, Atherosclerosis and Atheroprotection Are the Most Ancient on the Molecular Evolution Scale

As the second step, we performed an in silico phylostratigraphic analysis of all the 1068 human genes (see the central part of Figure 1). This was done using Orthoscape [107,108], our previously developed plugin for Cytoscape [109], a freely available web service. With Orthoscape, we obtained estimates of each gene’s phylostratigraphic age index (PAI) on the molecular evolution scale of the Basic Local Alignment Search Tool (BLAST) [106]. The results obtained are given in Table S1. This table also displays the arithmetic mean of the PAIs and the standard error of the mean (SEM) for each of the non-overlapping subsets of genes, these values being proportional to the heights of the gene bars and error bars in the chart in the central part of Figure 1. As can be seen, the gray-green bar for the subset of 16 human hub genes for atherogenesis, atherosclerosis and atheroprotection falls below the auxiliary dotted line, which is below the tops of the other five bars. This is statistically significant according to the binomial distribution (*p* < 0.05, (*) in the upper part of the chart). It means that the hub genes for atherogenesis, atherosclerosis and atheroprotection in the 16-strong set are the most ancient of those being studied.

Finally, as can be seen from the central part of this chart, the subset (a) of the hub genes for atherogenesis, atherosclerosis and atheroprotection is significantly (*p* < 0.05) more ancient than the subset (b) of 106 hub genes for atherogenesis and atherosclerosis, but without relevance to atheroprotection (gray-blue bar), according to the nonparametric Mann–Whitney U test and parametric Fischer’s Z-test (*p* < 0.05). This could be regarded as an additional piece of independent evidence in support of the conclusion that the subset of 16 human hub genes for atherogenesis, atherosclerosis and atheroprotection is the most ancient. With this in mind, we focused our further study on these 16 genes as on the fundamental feature that arose in many-celled animals of the subkingdom Eumetazoa, which have the nervous system, differentiated tissues and specialized intercellular contacts (Table S1: mean PIA = 4.06 ± 1.02) in the course of their evolution from more ancient forms of animals in the kingdom Metazoa (PIA = 3). If the genes in question promoted wound healing (as does atherogenesis or atheroprotection) in eumetazoans of reproductive age, then at later age a pathology like atherosclerosis [19] as an age-related disease [110] could be the cost for this adaptive advantage. This is consistent with the conclusions from an independent in silico modeling of evolution in heterogeneous microorganismal communities [111]. Thus we estimated the overall effect of atherogenesis, atherosclerosis and atheroprotection on the health of quite diverse humans, without belittling the importance of other genes described in Figure 1: (b) 106 hub genes for atherogenesis and atherosclerosis, (c) 21 hub genes for atheroprotection and atherosclerosis, (d) 10 genes specific for atheroprotection, (e) 58 genes specific for atherogenesis, and (f) 856 genes specific for atherosclerosis. The data on these genes provide molecular evidence of how animal complexity has grown on its way from Metazoa to Homo (PIA = 28).

### 2.3. Candidate SNP Markers Significantly Changing TBP for the Promoters of the Hub Genes for Atherogenesis, Atherosclerosis and Atheroprotection

**Figure 2:**
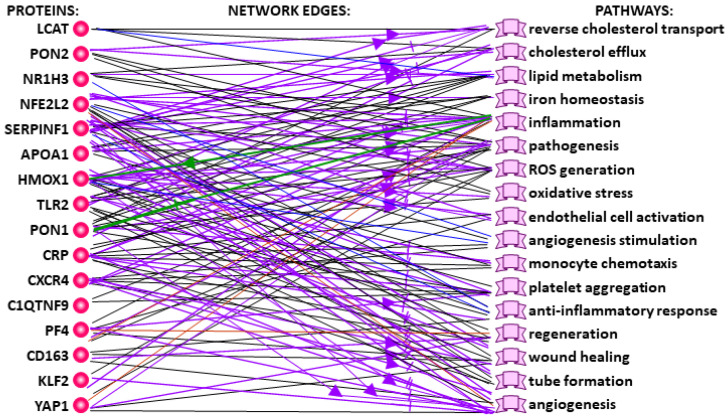
The associative network of signaling pathways (**right**) including the proteins (**left**) encoded by the 16 human hub genes for an atherogenesis, atherosclerosis and atheroprotection. The network was built using the automated mode of our publicly available web service ANDSystem [112] with “Human, proteins, APOA1, C1QTNF9, CD163, CRP, CXCR4, HMOX1, KLF2, LCAT, NFE2L2, NR1H3, PF4, PON1, PON2, SERPINF1, TLR2, YAP1, pathways” as input. Legend: Nodes: red circles, proteins. Proteins: APOA1, apolipoproteins A1; C1QTNF9, C1q and TNF related protein 9; CD163, molecules CD163; CRP, C-reactive protein; CXCR4, C-X-C motif chemokine receptor 4; HMOX1, heme oxygenase 1; KLF2, KLF transcription factors 2; LCAT, lecithin-cholesterol acyltransferase; NFE2L2, NFE2 like bZIP transcription factor 2; NR1H3, nuclear receptor subfamily 1 group H members 3; PF4, platelet factor 4; PON1, and PON2, paraoxonases 1 and 2, respectively; SERPINF1, serpin family F member 1; TLR2, toll like receptor 2; YAP1, Yes1 associated transcriptional regulator. Network edges: green barb arrow and T-shaped arrow: activation and deactivation, respectively; purple barb arrow and T-shaped arrow: positive and negative regulation, respectively; black, blue, brown and purple lines: association, involvement upregulation and regulation, respectively.

At the third, final step, we performed an in silico analysis of DNA sequences in the 90-bp proximal promoters before the starts of the protein-coding transcripts in each of the 16 human hub genes for atherogenesis, atherosclerosis and atheroprotection (see the lower part of Figure 1). For this purpose, we built an associative network of the proteins encoded by these human genes, with the use of our publicly available web service ANDSystem [112] run in the automated mode with “Human, proteins, APOA1, C1QTNF9, CD163, CRP, CXCR4, HMOX1, KLF2, LCAT, NFE2L2, NR1H3, PF4, PON1, PON2, SERPINF1, TLR2, YAP1, pathways” as input data, all the other parameters set at their default values (accessed on 15 March 2023). The result obtained is given in Figure 2.

This figure is a graphical rendering of the report following an intellectual “data mining” analysis of articles and databases about the molecular pathways in which the proteins encoded by human hub genes for atherogenesis, atherosclerosis and atheroprotection are involved. First of all, the figure displays “Reverse cholesterol transport”, “Cholesterol efflux” and “Lipid metabolism”, which are key for atherogenesis [113]. Furthermore, the molecular pathways “Inflammation”, “Oxidative stress” and “Reactive oxygen species (ROS) generation” in this associative network have been found to be relevant to the development of atherosclerosis [114]. Finally, “Regeneration”, “Wound healing” and “Anti-inflammatory response” are integral molecular pathways involved in atheroprotection [115]. With the use of this associative network (Figure 2) and the PubMed database [81] as accessed on 15 March 2023, we have annotated each of the 16 human hub genes for atherogenesis, atherosclerosis and atheroprotection (Figure 1) and answered the question as to how the deficiency or excess of their protein products accelerates or slows down the development of each of these three processes [116,117,118,119,120,121,122,123,124,125,126,127,128,129,130,131,132,133,134,135,136,137,138,139,140,141,142,143,144,145,146,147,148,149,150,151,152,153,154,155,156,157,158,159,160,161,162,163,164,165,166,167,168,169,170,171,172,173,174,175,176,177,178,179,180,181,182,183,184,185,186,187,188,189,190,191,192,193,194,195,196,197,198,199,200,201,202,203,204,205,206,207,208,209]. Our answers to this question can be found in Table S2.

Next, with our freely available web service SNP_TATA_Comparator [88] run in the automated mode, we analyzed all 5112 SNPs in the 90-bp proximal promoters before the starts of all protein-coding transcripts from each of the 16 human hub genes for atherogenesis, atherosclerosis and atheroprotection (see the bottom row of the table in the lower part of Figure 1). For example, the human CRP gene is one of the 16 hub genes for atherogenesis, atherosclerosis and atheroprotection (Figure 2). It encodes C-reactive protein as a key molecular marker for an inflammatory process in vessel walls, the development of atherosclerosis and an associated risk of cardiovascular diseases and their complications [130]. In the promoters located before the starts of all the five protein-coding transcripts from this gene, we found 226 SNPs, of which only two were consistent with a significant decrease in TBP affinity for these promoters (Table S3: rs1660782424:C and rs1660782480:G). In particular, this table includes SNP rs1660782480:G, in which G replaces A at position −28 relative to the start of transcript CRP-201 (position “+1”), according to Ensembl [35] and dbSNP [36]. According to our calculations described in Section 3.5, this substitution in the canonical TATA box (the TBP-binding site is in **bold type and underlined**) “tgctttgga**tAtaaat**ccagg => tgctttgga**t***G***taaat**ccagg” reduces TBP affinity for this promoter from normal 2.26 ± 0.23 nanomoles per liter (nM) for the equilibrium dissociation constant K_D_ of the TBP-promoter complex with the A nucleotide to mutant 7.64 ± 0.76 nM with the G nucleotide. Similarly, SNP rs1660782424:C replaces T with C at position −28 of the same TATA-box, namely: “tgctttgga**taTaaat**ccagg => tgctttgga**ta**C**aaat**ccagg”, which corresponds to a decrease in TBP affinity for the same promoter as follows: 2.26 ± 0.23 nM => 6.19 ± 0.62 nM (Table S3).

According to a large body of experimental data reported by independent authors (for review, see [67,68,69]), any decrease in TBP affinity for the promoter of a gene causes a decrease in the expression of this gene. In the example being considered, this provides evidence in favor of a reduction in C-reactive protein, which can have a beneficial effect on human health by retarding atherogenesis [130], enhancing atheroprotection in severe obesity [132] and reducing the risk of cardiovascular events in atherosclerosis [133].

As can be seen from the lower part of Figure 1, we have similarly revealed 330 candidate SNP markers, of which 150 decreased TBP affinity to the promoters of these hub genes and 316 increased it. According to multiple independent empirical observations [67], the former decreased the expression levels of these genes, while the latter increased them. An increased frequency of SNPs increasing TBP affinity for the promoters compared to that of SNPs decreasing it is significantly (Figure 1: P_ADJ_ < 10^−4^, binomial distribution with Bonferroni’s correction) different from the whole-genome ratio of their respective frequencies, according to the 1000 Genomes Project Consortium [32,210,211]. This implies that the human genome is under neutral drift [40,41]. This observation provides evidence that natural selection favors elevated expression of the human hub genes for atherogenesis, atherosclerosis and atheroprotection.

Finally, to understand whether natural selection favors or disfavors the candidate SNP markers that significantly changing TBP affinity for the promoters of the hub genes for atherogenesis, atherosclerosis and atheroprotection, we counted how many of these SNP markers improve or adversely affect human health as a result of their influence on these three processes (see Table S3, column “Effect on human health during atherosclerosis, atherogenesis and atheroprotection [Ref]”). The results obtained are given in Table 1.

As can be seen from this table, the candidate SNP markers that significantly change TBP affinity for the promoters of the human hub genes for atherogenesis, atherosclerosis and atheroprotection significantly improve human health indicators due to improved atheroprotection.

### 2.4. Verification of the Results Obtained by Analysis of Human Hub Genes for Atherogenesis, Atherosclerosis and Atheroprotection Using the Most Current Build of the ClinVar Database

First of all, we checked ClinVar [43] (accessed on 25 April 2023) for clinical data on each of 330 candidate SNP markers in hub genes for atherogenesis, atherosclerosis and atheroprotection, predicted for the first time in this work (Table S3). We found the only entry about a patient diagnosed as having “Osteogenesis Imperfecta, Recessive” with SNP rs541151948 in the SERPINF1 gene encoding protein serpin F1 (see Table S3). The clinical significance value for this ClinVar entry [43] was “Uncertain significance”, to indicate an unclear role of this SNP in this disease.

According to this table, this SNP has two minor alleles, rs541151948:A and rs541151948:T, when the nucleotide C (norm) is replaced by A and T, respectively, at position −60 relative to the start of the SERPINF1-205 transcript: “gagtgcaggtCgctttaagaa => gagtgcaggtAgctttaagaa” and “gagtgcaggtCgctttaagaa => gagtgcaggtTgctttaagaa”. These substitutions are consistent with an increase in TBP affinity to the promoter of this transcript from 10.21 ± 0.92 nM (norm) to 7.27 ± 0.51 nM and up to 8.53 ± 0.77 nM, respectively, which is statistically significant at *p* < 10^−6^ and *p* < 10^−6^ according to Fischer’s Z-test. This implies that the carriers of the minor alleles rs541151948:A and rs541151948:T can be expected to have excessive amounts of serpin F1, according to our estimates in Table S3.

As can be seen from this table, excessive serpin F1 in the models of human atherogenesis using senescent vascular smooth muscle cells is a biomarker of atherosclerotic plaques [185] and, consequently, narrowed blood vessels, as were clinically observed in the patient with osteogenesis imperfecta [192]. Additionally, according to a comprehensive overview [183], overexpression of SERPINF1 inhibited regeneration in mouse-based models of human wound healing [184]. A similar reduction in regeneration rates was observed in delayed fracture healing, according to a historical cohort study of patients with osteogenesis imperfecta [193]. Finally, elevated serpin F1 as a pro-inflammatory cytokine inhibitor guarantees a controllable reduced level of inflammatory processes [189,190,191], which were observed in a cohort study of children with osteogenesis imperfecta [194]. Thus, the phenotypical manifestations of excessive SERPINF1 in osteogenesis imperfecta are consistent with the symptoms of accelerated atherogenesis, reduced atheroprotection and mild atherosclerosis (Table S3).

Thus, our original proposal is to use the known clinical SNP marker rs541151948 for recessive osteogenesis imperfecta documented in ClinVar [43] as being a candidate SNP marker of accelerated atherogenesis, reduced atheroprotection and mild atherosclerosis.

Next, to elucidate the unknown role of the clinically known SNP-marker of recessive osteogenesis imperfecta, rs541151948, in the development of this disease, we propose the following possible molecular mechanism. Minor alleles rs541151948:A and rs541151948:T can significantly enhance serpin F1 levels, which can narrow blood vessels [185], delay regeneration in injury healing [183,184] and inhibit pro-inflammatory cytokines [189,190,191].

Finally, as the reader can see, with our resource-friendly in silico analysis of 5112 SNPs in the promoters of 16 hub genes for atherogenesis, atherosclerosis and atheroprotection, we have identified two candidate biomedical SNP markers, rs541151948:A and rs541151948:T, as being the most promising for a less resource-friendly cohort search for clinical SNP markers for personalized medicine, including their testing for the Hardy–Weinberg equilibrium [38].

### 2.5. Verification of the Results Obtained by Analysis of Human Hub Genes for Atherogenesis, Atherosclerosis and Atheroprotection Using RNA-Seq Data on Domestic and Wild Animals

Our further interest was to verify whether natural selection does favor human health improvement through overexpression of the hub genes for atherogenesis, atherosclerosis and atheroprotection, assuming the hypothesis about a role of self-domestication in human evolution [103,104]. To this end, we collected all freely available transcriptomes (RNA-Seq data) of domestic and wild animals (Table 2) using the PubMed databases [81]. As can be seen from the bottom row of Table 2, a total of 2905 differentially expressed genes (DEGs) have been found in nine tissues of seven domestic species and seven wild counterparts, according to 11 original works [212,213,214,215,216,217,218,219,220,221].

The 26 animal DEGs that we have found to be homologous to the human hub genes for atherogenesis, atherosclerosis and atheroprotection are listed in the right-hand part of Table S4; the effects that changes in the expression levels of these hub genes have on human health are listed in the left-hand part. As can be seen from the bottom row of the table in Figure 1, the DEGs whose expression levels are higher in domestic than wild animals occur at significantly higher frequencies than they would have if they had been under neutral drift [32,33,34,35,36,37,38,39,40,41] (P_ADJ_ < 0.05, binomial distribution with Bonferroni’s correction). This is consistent with a case of destabilizing selection as a necessary attribute in animal domestication [222].

Additionally, we compared changes in expression levels between (a) the human hub genes for atherogenesis, atherosclerosis and atheroprotection, and (b) the DEGs in the domestic animals (the upper part of Table 3). The lower part of this table contains estimates of the statistical significance of the correlations between the effects of same-direction changes in the expression levels in the homologous human genes on human health and in animals under domestication. As can be seen from the bottom line, elevated expression levels of the human hub genes for atherogenesis, atherosclerosis and atheroprotection, and of their homologs, are equally significant molecular markers for health improvement and domestication.

Thus, with the use of independent experimental RNA-Seq data on domestic and wild animals, and assuming the hypothesis that self-domestication has a role in human evolution [103,104], we significantly confirmed the fact that natural selection acts against underexpression of the human hub genes for atherogenesis, atherosclerosis and atheroprotection. At the same time, enhanced atheroprotection prevents the formation of atheromas, thus promoting health.

## 3. Materials and Methods

### 3.1. The Human Genes

We have studied the human genes in the lists of search results for keyword entries in the dialog box of the NCBI Gene database [105] accessed on 15 March 2023 with the following filters activated: “Genomic”, “Protein-coding”, “Annotated genes”, “Ensembl”, and “Current”. With [“atherogenesis” AND “Homo sapiens”] as input data, a list of 180 protein-coding human genes associated with atherogenesis was produced. Similarly, the use of input data in the form of [“atherosclerosis” AND “Homo sapiens”] produced a list of 999 human genes associated with atherosclerosis, while [“atheroprotective” AND “Homo sapiens”] produced a list of 47 human genes associated with atheroprotection (see Figure 1). Taking into account the overlaps between these three lists in the Venn diagram (Figure 1), we have studied 1068 human genes (see Table S1).

### 3.2. In Silico Assessment of the BLAST-Based PAIs for a Human Gene

We calculated the BLAST-based [106] PAI for an arbitrary human gene using its NCBI Entrez gene number as input data for our Orthoscape plug-in [107,108] within the Cytoscape software suite [109]. The output was the most recent common ancestor of all animal species in which the DNA sequence of this gene is known. The following evolutionary rank scale was used: 0, Cellular organisms; 1, Eukaryota; 2, Opisthokonta; 3, Metazoa; 4, Eumetazoa; 5, Bilateria; 6, Deuterostomia; 7, Chordata; 8, Craniata; 9, Vertebrata; 10, Gnathostomata; 11, Teleostomi; 12, Euteleostomi; 13, Sarcopterygii; 14, Dipnotetrapodomorpha; 15, Tetrapoda; 16, Amniota; 17, Mammalia; 18, Theria; 19, Eutheria; 20, Euarchontoglires; 21, Primates; 22, Haplorrhini; 23, Simiiformes; 24, Catarrhini; 25, Hominoidea; 26, Hominidae; 27, Homininae; and 28, Homo.

### 3.3. Data Mining of Literature Sources and Databases Publicly Available on the Internet

We performed data mining using our previously published freely available web service ANDSystem [112] run in the automated mode, with “Human, proteins, APOA1, C1QTNF9, CD163, CRP, CXCR4, HMOX1, KLF2, LCAT, NFE2L2, NR1H3, PF4, PON1, PON2, SERPINF1, TLR2, YAP1, pathways” as input data, all the other parameters set at their default values. As a result, we built the associative network shown in Figure 2, which characterizes the participation of the above-mentioned proteins encoded by the human hub genes for atherogenesis, atherosclerosis and atheroprotection (left-hand border) in the molecular pathways (right-hand border), according to ANDSystem [112] accessed on 15 March 2023.

### 3.4. DNA Sequences

For in silico analysis of the human hub genes for atherogenesis, atherosclerosis and atheroprotection, we retrieved the DNA sequences and SNPs of their 90-bp proximal promoters from Ensembl [35] and dbSNP [36], respectively, with SNP_TATA_Comparator run in the automated mode [88], when it uses the Bioperl library [223] for access to these databases.

### 3.5. In Silico Analysis of DNA Sequences

We analyzed the SNPs in the DNA sequences with SNP_TATA_Comparator [88] run in the automated mode, and this analysis was identical to those described previously [88,103]. It implements our bioinformatic model of the three-stage binding of the TATA-binding protein (TBP) to a given 90-bp proximal promoter of the human gene [224,225,226,227] (for the most detailed description, see Section S1 “Supplementary methods for DNA sequence analysis”).

### 3.6. DEGs

We have analyzed all independent experimental RNA-Seq datasets of the transcriptomes of tissues from domestic versus wild animals [212,213,214,215,216,217,218,219,220,221] publicly available in the PubMed database [81], as accessed on 15 March 2023. The lists of homologous genes were taken from the section “Paralogs” of the freely available GeneCards database [228], as accessed on 15 March 2023.

### 3.7. Human_SNP_TATAdb and PetDEGsDB: Our All-New Knowledge Bases

We have documented (1) the candidate SNP markers that we have found to significantly change TBP affinity for the promoters of 16 human hub genes for atherogenesis, atherosclerosis and atheroprotection, and (2) the estimates of the potential effect of these molecular markers on human health (Table S3) in an Excel-compatible flat file. Similarly, we have generated a similar Excel file for associations between the human hub genes and the DEGs (Table S4), and added it to PetDEGsDB, a freely available knowledge base, with its new assembly accessible at www.sysbio.ru/domestic-wild (accessed on 15 March 2023) in the MariaDB 10.2.12 database management system (MariaDB Corporation Ab, Espoo, Finland).

Finally, we have similarly generated another knowledge base for the candidate SNP markers that significantly change TBP affinity for human gene promoters, Human_SNP_TATAdb, its first version being publicly accessible at www.sysbio.ru/Human_SNP_TATAdb in MariaDB 10.2.12 (as accessed on 15 March 2023).

### 3.8. Statistical Analysis

We performed the Mann–Whitney U test, Fisher’s Z-test, and an exact test for the binomial distribution using appropriate options in STATISTICA (Statsoft^TM^).

## 4. Conclusions

We have for the first time estimated the phylostratigraphic age indices (PAIs) of all 1068 human genes associated by the most current NCBI gene build [105] with atherogenesis, atherosclerosis and an atheroprotection and placed them on the BLAST-based molecular evolution scale (Table S1). We have thus found that the 16-strong set of hub genes regulating these three processes is the most ancient (Figure 1).

Next, we have for the first time performed an in silico assessment of the effects of all SNPs localized in 90-bp promoters before the starts of all protein-coding transcripts from these 16 hub genes on TBP affinity for these promoters according to Ensembl [35] and dbSNP [36] as accessed on 15 March 2023. We have thus found 330 candidate SNP markers that significantly change this affinity and, therefore, the expression of the hub genes for atherogenesis, atherosclerosis and atheroprotection, and, consequently, have a bearing on human health. We have found that natural selection acts against underexpression of the hub genes for atherogenesis, atherosclerosis and atheroprotection, and, due to enhanced atheroprotection, favors human health improvement.

Finally, we have verified all 330 candidate SNP markers in the hub genes for atherogenesis, atherosclerosis and atheroprotection with the use of the ClinVar [43] database (accessed on 25 April 2023). We have thus for the first time proposed to use the known clinical SNP marker rs541151948 for recessive osteogenesis imperfecta [43] as a candidate SNP marker of accelerated atherogenesis, reduced atheroprotection and mild atherosclerosis.

## Figures and Tables

**Table 1:** Candidate SNP makers significantly changing TBP affinity for the promoters of the human hub genes for atherogenesis, atherosclerosis and atheroprotection.

Atherosclerosis-Related Processes	The Number of Candidate SNP Markers That Have Been Found to Significantly Change TBP Affinity for the Promoters of the Hub Genes for Atherogenesis, Atherosclerosis and Atheroprotection		Statistical Significance	
	Human Health Failure	Human Health Improvement	Binomial Distribution, *p*	Bonferroni’s Correction P_ADJ_.
**Atherogenesis**	186	144	0.05	1.00
**Atherosclerosis**	195	135	10^−3^	0.19
**Atheroprotection**	91	239	10^−6^	10^−4^

**Table 2:** RNA-Seq data on domestic animals versus their wild counterparts (PubMed data [81]).

#	Domestic Animals	Wild Animals	Tissue	N_DEG_	Ref.
1	tame rats	aggressive rats	hypothalamus	46	[212]
2	tame rats	aggressive rats	hippocampus	42	[99]
3	tame rats	aggressive rats	midbrain tegmentum	31	[213]
4	tame rats	aggressive rats	periaqueductal gray matter	39	[214]
5	tame rats	aggressive rats	frontal cortex	20	[215]
6	guinea pigs	cavy	frontal cortex	883	[215]
7	domestic rabbits	wild rabbits	frontal cortex	17	[215]
8	domestic rabbits	wild rabbits	parietal-temporal cortex	216	[216]
9	domestic rabbits	wild rabbits	amygdala	118	[216]
10	domestic rabbits	wild rabbits	hypothalamus	43	[216]
11	domestic rabbits	wild rabbits	hippocampus	100	[216]
12	dogs	wolves	blood	450	[217]
13	dogs	wolves	frontal cortex	13	[215]
14	tame foxes	aggressive foxes	pituitary	327	[218]
15	pigs	boars	frontal cortex	30	[215]
16	pigs	boars	frontal cortex	34	[219]
17	pigs	boars	pituitary	22	[220]
18	domestic chicken	wild chicken	pituitary	474	[221]
Σ	7 domestic animal species	7 wild animal species	9 tissues	2905	11 Refs

**Table 3:** Verification of the results obtained by analysis of human hub genes for atherogenesis, atherosclerosis and atheroprotection using RNA-Seq data on domestic and wild animals.

	(a) Humans	The Effect of Changes in the Expression of the Human Hub Genes for Atherogenesis, Atherosclerosis and Atheroprotection on Human Health during Each of These Three Processes						
		Atherogenesis		Atherosclerosis		Atheroprotection		
(b) Domestic Animals		Worsen	Relief	Worsen	Relief	Worsen	Relief	
The number of DEGs that have their expression levels changed in the same direction as have their homologous human hub genes for atherogenesis, atherosclerosis and atheroprotection, with the effect on human health as indicated		10	16	14	12	2	24	
Statistical significance	Binomial distribution, *p*	0.16		0.42		10^−5^		
	Bonferroni’s correction, P_ADJ_	1.00		1.00		10^−3^		

## Data Availability

Not applicable.

## Supplementary Materials

The supporting information can be downloaded at: https://www.mdpi.com/article/10.3390/ijms24109010/s1.

## Abbreviations

BLAST	basic local alignment search tool
DEG	differentially expressed gene
FH	familial hypercholesterolemia
PAI	phylostratigraphic age index
RNA-Seq	RNA sequencing
ROS	reactive oxygen species
SNP	single-nucleotide polymorphism
TBP	TATA-binding protein
WHO	World Health Organization

## References

[1] Zhou F., Zhu X., Liu Y., Sun Y., Zhang Y., Cheng D., Wang W. (2023). Coronary atherosclerosis and chemotherapy: From bench to bedside. Front. Cardiovasc. Med..

[2] Yu X., Yang X., Cao J. (2023). Application of single-cell genomics in cardiovascular research. Cardiol. Ther..

[3] Yang X.H., Zhang B.L., Cheng Y., Fu S.K., Jin H.M. (2023). Association of remnant cholesterol with risk of cardiovascular disease events, stroke, and mortality: A systemic review and meta-analysis. Atherosclerosis.

[4] Barquera S., Pedroza-Tobias A., Medina C., Hernandez-Barrera L., Bibbins-Domingo K., Lozano R., Moran A.E. (2015). Global overview of the epidemiology of atherosclerotic cardiovascular disease. Arch. Med. Res..

[5] Xiang Q., Tian F., Xu J., Du X., Zhang S., Liu L. (2022). New insight into dyslipidemia-induced cellular senescence in atherosclerosis. Biol. Rev. Camb. Philos. Soc..

[6] Esfarjani S.V., Zakerkish M. (2022). Dyslipidemia in youth: Epidemiology, pathophysiology, screening, management, and treatment: A review of the literature. J. Family Med. Prim. Care..

[7] Perumalsamy S., Huri H.Z., Abdullah B.M., Mazlan O., Wan Ahmad W.A., Vethakkan S.R.D.B. (2023). Genetic markers of insulin resistance and atherosclerosis in type 2 diabetes mellitus patients with coronary artery disease. Metabolites.

[8] Pamminger M., Mayr A. (2022). Cardiovascular consequences of smoking: Imaging overview. Radiologie.

[9] Vesnina A., Prosekov A., Atuchin V., Minina V., Ponasenko A. (2022). Tackling atherosclerosis via selected nutrition. Int. J. Mol. Sci..

[10] Napoli C., D’Armiento F.P., Mancini F.P., Postiglione A., Witztum J.L., Palumbo G., Palinski W. (1997). Fatty streak formation occurs in human fetal aortas and is greatly enhanced by maternal hypercholesterolemia. Intimal accumulation of low density lipoprotein and its oxidation precede monocyte recruitment into early atherosclerotic lesions. J. Clin. Invest..

[11] Goldstein J., Hobbs H., and Brown M., Scriver C., Beaudet A., Sly W., Valle D. (2001). Familial hypercholesterolemia. The Metabolic and Molecular Bases of Inherited Disease.

[12] Sun X.M., Patel D.D., Webb J.C., Knight B.L., Fan L.M., Cai H.J., Soutar A.K. (1994). Familial hypercholesterolemia in China: Identification of mutations in the LDL-receptor gene that result in a receptor-negative phenotype. Arterioscler. Thromb..

[13] Spence J.D. (2017). Rational medical therapy is the key to effective cardiovascular disease prevention. Can. J. Cardiol..

[14] Goldberg D., Khatib S. (2022). Atherogenesis, transcytosis, and the transmural cholesterol flux: A critical review. Oxid. Med. Cell. Longev..

[15] Lee-Rueckert M., Lappalainen J., Kovanen P.T., Escola-Gil J.C. (2022). Lipid-laden macrophages and inflammation in atherosclerosis and cancer: An integrative view. Front. Cardiovasc. Med..

[16] Li A.C., Glass C.K. (2002). The macrophage foam cell as a target for therapeutic intervention. Nat. Med..

[17] Lusis A.J. (2000). Atherosclerosis. Nature.

[18] Hirayama S., Soda S., Ito Y., Matsui H., Ueno T., Fukushima Y., Ohmura H., Hanyu O., Aizawa Y., Miida T. (2010). Circadian change of serum concentration of small dense LDL-cholesterol in type 2 diabetic patients. Clin. Chim. Acta.

[19] Lathe R., Sapronova A., Kotelevtsev Y. (2014). Atherosclerosis and Alzheimer—Diseases with a common cause? Inflammation, oxysterols, vasculature. BMC Geriatr..

[20] Makarova Y.A., Ryabkova V.A., Salukhov V.V., Sagun B.V., Korovin A.E., Churilov L.P. (2023). Atherosclerosis, cardiovascular disorders and COVID-19: Comorbid pathogenesis. Diagnostics.

[21] Petrucci G., Rizzi A., Hatem D., Tosti G., Rocca B., Pitocco D. (2022). Role of oxidative stress in the pathogenesis of atherothrombotic diseases. Antioxidants.

[22] Chen S., Wu X., Li T., Li Y., Wang B., Cheng W., Teng Y., Yang J., Meng H., Wang L. (2021). Atheroprotective effects and mechanisms of postmarketing Chinese patent formulas in atherosclerosis models: A systematic review. Evid. Based Complement. Alternat. Med..

[23] Wu X., Wang T.T.Y., Prior R.L., Pehrsson P.R. (2018). Prevention of atherosclerosis by berries: The case of blueberries. J. Agric. Food Chem..

[24] Smith J.K. (2001). Exercise and atherogenesis. Exerc. Sport. Sci. Rev..

[25] Schatz U., Schettler V.J.J., Julius U. (2023). State of the art: Lipoprotein apheresis. Dtsch. Med. Wochenschr..

[26] Luoma P.V. (1997). Gene activation, apolipoprotein A-I/high density lipoprotein, atherosclerosis prevention and longevity. Pharmacol. Toxicol..

[27] Glass C.K., Witztum J.L. (2001). Atherosclerosis: The road ahead. Cell..

[28] Ahmad P., Alvi S.S., Iqbal D., Khan M.S. (2020). Insights into pharmacological mechanisms of polydatin in targeting risk factors-mediated atherosclerosis. Life Sci..

[29] Pal K., Manescu I.B., Lupu S., Dobreanu M. (2023). Emerging biomarkers for predicting clinical outcomes in patients with heart disease. Life.

[30] Trovato G.M. (2014). Sustainable medical research by effective and comprehensive medical skills: Overcoming the frontiers by predictive, preventive and personalized medicine. EPMA J..

[31] Lowy-Gallego E., Fairley S., Zheng-Bradley X., Ruffier M., Clarke L., Flicek P. (2019). 1000 Genomes Project Consortium. Variant calling on the GRCh38 assembly with the data from phase three of the 1000 Genomes Project. Wellcome Open. Res..

[32] Birney E. (2021). The International Human Genome Project. Hum. Mol. Genet..

[33] Telenti A., Pierce L.C., Biggs W.H., di Iulio J., Wong E.H., Fabani M.M., Kirkness E.F., Moustafa A., Shah N., Xie C. (2016). Deep sequencing of 10,000 human genomes. Proc. Natl. Acad. Sci. USA.

[34] Singh V., Pandey S., Bhardwaj A. (2022). From the reference human genome to human pangenome: Premise, promise and challenge. Front. Genet..

[35] Zerbino D., Wilder S., Johnson N., Juettemann T., Flicek P. (2015). The Ensembl regulatory build. Genom. Biol..

[36] Day I.N. (2010). dbSNP in the detail and copy number complexities. Hum. Mutat..

[37] Lam C.W., Lau K.C., Tong S.F. (2010). Microarrays for personalized genomic medicine. Adv. Clin. Chem..

[38] Pergialiotis V., Fanaki M., Bellos I., Stefanidis K., Loutradis D., Daskalakis G. (2020). The impact of vascular endothelial growth factor single nucleotide polymorphisms in the development and severity of endometriosis: A systematic review of the literature. J. Gynecol. Obstet. Hum. Reprod..

[39] Pocai B. (2019). The ICD-11 has been adopted by the World Health Assembly. World Psychiatry.

[40] Haldane J.B.S. (1957). The cost of natural selection. J. Genet..

[41] Kimura M. (1968). Evolutionary rate at the molecular level. Nature.

[42] Amberger J., Bocchini C.A., Scott A.F., Hamosh A. (2009). McKusick’s Online Mendelian Inheritance in Man (OMIM). Nucleic Acids Res..

[43] Landrum M.J., Lee J.M., Riley G.R., Jang W., Rubinstein W.S., Church D.M., Maglott D.R. (2014). ClinVar: Public archive of relationships among sequence variation and human phenotype. Nucleic Acids Res..

[44] Beck T., Rowlands T., Shorter T., Brookes A.J. (2023). GWAS Central: An expanding resource for finding and visualising genotype and phenotype data from genome-wide association studies. Nucleic Acids Res..

[45] Yotova R. (2020). Regulating genome editing under international human rights law. Int. Comp. Law Q..

[46] De Plano L.M., Calabrese G., Conoci S., Guglielmino S.P.P., Oddo S., Caccamo A. (2022). Applications of CRISPR-Cas9 in Alzheimer’s disease and related disorders. Int. J. Mol. Sci..

[47] Deplancke B., Alpern D., Gardeux V. (2016). The genetics of transcription factor DNA binding variation. Cell.

[48] Colognesi I., Pasquali V., Foa A., Renzi P., Bernardi F., Bertolucci C., Pinotti M. (2007). Temporal variations of coagulation factor VII activity in mice are influenced by lighting regime. Chronobiol. Int..

[49] Wu K.S., Zhou X., Zheng F., Xu X.Q., Lin Y.H., Yang J. (2010). Influence of interleukin-1 beta genetic polymorphism, smoking and alcohol drinking on the risk of non-small cell lung cancer. Clin. Chim. Acta.

[50] Al-Shakfa F., Dulucq S., Brukner I., Milacic I., Ansari M., Beaulieu P., Moghrabi A., Laverdiere C., Sallan S., Silverman L. (2009). DNA variants in region for noncoding interfering transcript of dihydrofolate reductase gene and outcome in childhood acute lymphoblastic leukemia. Clin. Cancer Res..

[51] Kopp C.W., Kopp H.P., Steiner S., Kriwanek S., Krzyzanowska K., Bartok A., Roka R., Minar E., Schernthaner G. (2003). Weight loss reduces tissue factor in morbidly obese patients. Obes. Res..

[52] Belancic A., Strbad T., Kucan Stiglic M., Vitezic D. (2023). Effectiveness of Nusinersen in type 1, 2 and 3 spinal muscular atrophy: Croatian real-world data. J. Clin. Med..

[53] Ren N., Dai S., Ma S., Yang F. (2023). Strategies for activity analysis of single nucleotide polymorphisms associated with human diseases. Clin. Genet..

[54] Ioshikhes I., Trifonov E.N., Zhang M.Q. (1999). Periodical distribution of transcription factor sites in promoter regions and connection with chromatin structure. Proc. Natl. Acad. Sci. USA.

[55] Bednar J., Horowitz R.A., Dubochet J., Woodcock C.L. (1995). Chromatin conformation and salt-induced compaction: Three-dimensional structural information from cryoelectron microscopy. J. Cell. Biol..

[56] Ponomarenko M., Mironova V., Gunbin K., Savinkova L., Maloy S., Hughes K. (2013). Hogness Box. Brenner’s Encyclopedia of Genetics.

[57] Tsai F.T., Sigler P.B. (2000). Structural basis of preinitiation complex assembly on human pol II promoters. EMBO J..

[58] Frank J. (2002). A cold look at transcription. Structure.

[59] Kornberg R.D. (1998). Mechanism and regulation of yeast RNA polymerase II transcription. Cold Spring Harb. Symp. Quant. Biol..

[60] Martianov I., Viville S., Davidson I. (2002). RNA polymerase II transcription in murine cells lacking the TATA binding protein. Science.

[61] Muller F., Lakatos L., Dantonel J., Strahle U., Tora L. (2001). TBP is not universally required for zygotic RNA polymerase II transcription in zebrafish. Curr. Biol..

[62] Yang M.Q., Laflamme K., Gotea V., Joiner C.H., Seidel N.E., Wong C., Petrykowska H.M., Lichtenberg J., Lee S., Welch L. (2011). Genome-wide detection of a TFIID localization element from an initial human disease mutation. Nucleic Acids Res..

[63] Rhee H.S., Pugh B.F. (2012). Genome-wide structure and organization of eukaryotic pre-initiation complexes. Nature.

[64] Choukrallah M.A., Kobi D., Martianov I., Pijnappel W.W., Mischerikow N., Ye T., Heck A.J., Timmers H.T., Davidson I. (2012). Interconversion between active and inactive TATA-binding protein transcription complexes in the mouse genome. Nucleic Acids Res..

[65] Borggrefe T., Davis R., Bareket-Samish A., Kornberg R.D. (2001). Quantitation of the RNA polymerase II transcription machinery in yeast. J. Biol. Chem..

[66] Kim T.K., Hashimoto S., Kelleher R.J., Flanagan P.M., Kornberg R.D., Horikoshi M., Roeder R.G. (1994). Effects of activation-defective TBP mutations on transcription initiation in yeast. Nature.

[67] Mogno I., Vallania F., Mitra R.D., Cohen B. (2010). TATA is a modular component of synthetic promoters. Genome Res..

[68] Ponomarenko P., Suslov V., Savinkova L., Ponomarenko M., Kolchanov N. (2010). A precise equilibrium equation for four steps of binding between TBP and TATA-box allows for the prediction of phenotypical expression upon mutation. Biofizika.

[69] Ravarani C.N., Chalancon G., Breker M., de Groot N.S., Babu M.M. (2016). Affinity and competition for TBP are molecular determinants of gene expression noise. Nat. Commun..

[70] Fornes O., Gheorghe M., Richmond P.A., Arenillas D.J., Wasserman W.W., Mathelier A. (2018). MANTA2, update of the Mongo database for the analysis of transcription factor binding site alterations. Sci. Data.

[71] Abramov S., Boytsov A., Bykova D., Penzar D.D., Yevshin I., Kolmykov S.K., Fridman M.V., Favorov A.V., Vorontsov I.E., Baulin E. (2021). Landscape of allele-specific transcription factor binding in the human genome. Nat. Commun..

[72] Ponomarenko P., Savinkova L., Drachkova I., Lysova M., Arshinova T., Ponomarenko M., Kolchanov N. (2008). A step-by-step model of TBP/TATA box binding allows predicting human hereditary diseases by single nucleotide polymorphism. Dokl. Biochem. Biophys..

[73] Coleman R.A., Pugh B.F. (1995). Evidence for functional binding and stable sliding of the TATA binding protein on nonspecific DNA. J. Biol. Chem..

[74] Dickerson R.E., Drew H.R. (1981). Structure of a B-DNA dodecamer. II. Influence of base sequence on helix structure. J. Mol. Biol..

[75] Bucher P. (1990). Weight matrix descriptions of four eukaryotic RNA polymerase II promoter elements derived from 502 unrelated promoter sequences. J. Mol. Biol..

[76] Berg O.G., von Hippel P.H. (1987). Selection of DNA binding sites by regulatory proteins. Statistical-mechanical theory and application to operators and promoters. J. Mol. Biol..

[77] Flatters D., Lavery R. (1998). Sequence-dependent dynamics of TATA-box binding sites. Biophys. J..

[78] Kim J.L., Nikolov D.B., Burley S.K. (1993). Co-crystal structure of TBP recognizing the minor groove of a TATA element. Nature.

[79] Kim Y., Geiger J.H., Hahn S., Sigler P.B. (1993). Crystal structure of a yeast TBP/TATA-box complex. Nature.

[80] Delgadillo R.F., Whittington J.E., Parkhurst L.K., Parkhurst L.J. (2009). The TATA-binding protein core domain in solution variably bends TATA sequences via a three-step binding mechanism. Biochemistry.

[81] Lu Z. (2011). PubMed and beyond: A survey of web tools for searching biomedical literature. Database.

[82] Arkova O., Kuznetsov N., Fedorova O., Kolchanov N., Savinkova L. (2014). Realtime interaction between TBP and the TATA box of the human triosephosphate isomerase gene promoter in the norm and pathology. Acta Nat..

[83] Arkova O., Kuznetsov N., Fedorova O., Savinkova L. (2017). A real-time study of the interaction of TBP with a TATA box-containing duplex identical to an ancestral or minor allele of human gene LEP or TPI. J. Biomol. Struct. Dyn..

[84] Savinkova L., Drachkova I., Arshinova T., Ponomarenko P., Ponomarenko M., Kolchanov N. (2013). An experimental verification of the predicted effects of promoter TATA-box polymorphisms associated with human diseases on interactions between the TATA boxes and TATA-binding protein. PLoS ONE.

[85] Drachkova I., Savinkova L., Arshinova T., Ponomarenko M., Peltek S., Kolchanov N. (2014). The mechanism by which TATA-box polymorphisms associated with human hereditary diseases influence interactions with the TATA-binding protein. Hum. Mutat..

[86] Rasskazov D., Chadaeva I., Sharypova E., Zolotareva K., Khandaev B., Ponomarenko P., Podkolodnyy N., Tverdokhleb N., Vishnevsky O., Bogomolov A. (2022). Plant_SNP_TATA_Z-Tester: A Web service that unequivocally estimates the impact of proximal promoter mutations on plant gene expression. Int. J. Mol. Sci..

[87] Ponomarenko P., Chadaeva I., Rasskazov D.A., Sharypova E., Kashina E.V., Drachkova I., Zhechev D., Ponomarenko M.P., Savinkova L.K., Kolchanov N. (2017). Candidate SNP markers of familial and sporadic Alzheimer’s diseases are predicted by a significant change in the affinity of TATA-binding protein for human gene promoters. Front. Aging Neurosci..

[88] Ponomarenko M., Rasskazov D., Arkova O., Ponomarenko P., Suslov V., Savinkova L., Kolchanov N. (2015). How to use SNP_TATA_Comparator to find a significant change in gene expression caused by the regulatory SNP of this gene’s promoter via a change in affinity of the TATA-binding protein for this promoter. Biomed. Res. Int..

[89] Waardenberg A., Basset S., Bouveret R., Harvey R. (2015). CompGO: An R package for comparing and visualizing Gene Ontology enrichment differences between DNA binding experiments. BMC Bioinform..

[90] Ponomarenko M.P., Arkova O., Rasskazov D., Ponomarenko P., Savinkova L., Kolchanov N. (2016). Candidate SNP markers of gender-biased autoimmune complications of monogenic diseases are predicted by a significant change in the affinity of TATA-binding protein for human gene promoters. Front. Immunol..

[91] Chadaeva I.V., Ponomarenko M.P., Rasskazov D.A., Sharypova E.B., Kashina E.V., Matveeva M.Y., Arshinova T.V., Ponomarenko P.M., Arkova O.V., Bondar N.P. (2016). Candidate SNP markers of aggressiveness-related complications and comorbidities of genetic diseases are predicted by a significant change in the affinity of TATA-binding protein for human gene promoters. BMC Genom..

[92] Ponomarenko P., Rasskazov D., Suslov V., Sharypova E., Savinkova L., Podkolodnaya O., Podkolodny N.L., Tverdokhleb N.N., Chadaeva I., Ponomarenko M. (2016). Candidate SNP markers of chronopathologies are predicted by a significant change in the affinity of TATA-binding protein for human gene promoters. Biomed. Res. Int..

[93] Kimura M. (1980). A simple method for estimating evolutionary rates of base substitutions through comparative studies of nucleotide sequences. J. Mol. Evol..

[94] Hamilton M.B., Braverman J.M., Soria-Hernanz D.F. (2003). Patterns and relative rates of nucleotide and insertion/deletion evolution at six chloroplast intergenic regions in new world species of the Lecythidaceae. Mol. Biol. Evol..

[95] Li W.H., Wu C.I., Luo C.C. (1985). A new method for estimating synonymous and nonsynonymous rates of nucleotide substitution considering the relative likelihood of nucleotide and codon changes. Mol. Biol. Evol..

[96] Chadaeva I., Ponomarenko P., Rasskazov D., Sharypova E., Kashina E., Kleshchev M., Ponomarenko M., Naumenko V., Savinkova L., Kolchanov N. (2019). Natural selection equally supports the human tendencies in subordination and domination: A genome-wide study with in silico confirmation and in vivo validation in mice. Front. Genet..

[97] Oshchepkov D., Ponomarenko M., Klimova N., Chadaeva I., Bragin A., Sharypova E., Shikhevich S., Kozhemyakina R. (2019). A rat model of human behavior provides evidence of natural selection against underexpression of aggressiveness-related genes in humans. Front. Genet..

[98] Klimova N.V., Oshchepkova E., Chadaeva I., Sharypova E., Ponomarenko P., Drachkova I., Rasskazov D., Oshchepkov D., Ponomarenko M., Savinkova L. (2021). Disruptive selection of human immunostimulatory and immunosuppressive genes both provokes and prevents rheumatoid arthritis, respectively, as a self-domestication syndrome. Front. Genet..

[99] Oshchepkov D., Chadaeva I., Kozhemyakina R., Zolotareva K., Khandaev B., Sharypova E., Ponomarenko P., Bogomolov A., Klimova N.V., Shikhevich S. (2022). Stress reactivity, susceptibility to hypertension, and differential expression of genes in hypertensive compared to normotensive patients. Int. J. Mol. Sci..

[100] Ponomarenko M., Kleshchev M., Ponomarenko P., Chadaeva I., Sharypova E., Rasskazov D., Kolmykov S., Drachkova I., Vasiliev G., Gutorova N. (2020). Disruptive natural selection by male reproductive potential prevents underexpression of protein-coding genes on the human Y chromosome as a self-domestication syndrome. BMC Genet..

[101] Ponomarenko M., Rasskazov D., Chadaeva I., Sharypova E., Drachkova I., Ponomarenko P., Oshchepkova E., Savinkova L., Kolchanov N. (2019). Candidate SNP-markers of atherosclerosis, which may significantly change the affinity of the TATA-binding protein for the human gene promoters. Russ. J. Genet..

[102] Ponomarenko M., Rasskazov D., Chadaeva I., Sharypova E., Drachkova I., Oshchepkov D., Ponomarenko P., Savinkova L., Oshchepkova E., Nazarenko M. (2020). Candidate SNP markers of atherogenesis significantly shifting the affinity of TATA-binding protein for human gene promoters show stabilizing natural selection as a sum of neutral drift accelerating atherogenesis and directional natural selection slowing it. Int. J. Mol. Sci..

[103] Theofanopoulou C., Gastaldon S., O’Rourke T., Samuels B.D., Martins P.T., Delogu F., Alamri S., Boeckx C. (2017). Self-domestication in Homo sapiens: Insights from comparative genomics. PLoS ONE.

[104] Del Savio L., Mameli M. (2020). Human domestication and the roles of human agency in human evolution. Hist. Philos. Life Sci..

[105] Brown G.R., Hem V., Katz K.S., Ovetsky M., Wallin C., Ermolaeva O., Tolstoy I., Tatusova T., Pruitt K.D., Maglott D.R. (2015). Gene: A gene-centered information resource at NCBI. Nucleic Acids Res..

[106] Altschul S.F., Gish W., Miller W., Myers E.W., Lipman D.J. (1990). Basic local alignment search tool. J. Mol. Biol..

[107] Mustafin Z.S., Lashin S.A., Matushkin Y.G., Gunbin K.V., Afonnikov D.A. (2017). Orthoscape: A cytoscape application for grouping and visualization KEGG based gene networks by taxonomy and homology principles. BMC Bioinform..

[108] Mustafin Z.S., Zamyatin V.I., Konstantinov D.K., Doroshkov A.V., Lashin S.A., Afonnikov D.A. (2019). Phylostratigraphic analysis shows the earliest origination of the abiotic stress associated genes in A. thaliana. Genes.

[109] Shannon P., Markiel A., Ozier O., Baliga N.S., Wang J.T., Ramage D., Amin N., Schwikowski B., Ideker T. (2003). Cytoscape: A software environment for integrated models of biomolecular interaction networks. Genome Res..

[110] Kishimoto Y., Kondo K., Momiyama Y. (2021). The protective role of sestrin2 in atherosclerotic and cardiac diseases. Int. J. Mol. Sci..

[111] Klimenko A., Matushkin Y., Kolchanov N., Lashin S. (2021). Leave or stay: Simulating motility and fitness of microorganisms in dynamic aquatic ecosystems. Biology.

[112] Ivanisenko V.A., Demenkov P.S., Ivanisenko T.V., Mishchenko E.L., Saik O.V. (2019). A new version of the ANDSystem tool for automatic extraction of knowledge from scientific publications with expanded functionality for reconstruction of associative gene networks by considering tissue-specific gene expression. BMC Bioinform..

[113] Jiang X.C., Yu Y. (2021). The role of phospholipid transfer protein in the development of atherosclerosis. Curr. Atheroscler. Rep..

[114] Wu T., Peng Y., Yan S., Li N., Chen Y., Lan T. (2018). Andrographolide ameliorates atherosclerosis by suppressing pro-inflammation and ROS generation-mediated foam cell formation. Inflammation.

[115] Viola J., Soehnlein O. (2015). Atherosclerosis—A matter of unresolved inflammation. Semin. Immunol..

[116] Hewing B., Parathath S., Barrett T., Chung W.K., Astudillo Y.M., Hamada T., Ramkhelawon B., Tallant T.C., Yusufishaq M.S., Didonato J.A. (2014). Effects of native and myeloperoxidase-modified apolipoprotein a-I on reverse cholesterol transport and atherosclerosis in mice. Arterioscler. Thromb. Vasc. Biol..

[117] Trieu V.N., Uckun F.M. (1999). Apolipoprotein(a), a link between atherosclerosis and tumor angiogenesis. Biochem. Biophys. Res. Commun..

[118] Birchbauer A., Knipping G., Juritsch B., Aschauer H., Zechner R. (1993). Characterization of the apolipoprotein AI and CIII genes in the domestic pig. Genomics.

[119] Liu M., Li W., Wang H., Yin L., Ye B., Tang Y., Huang C. (2019). CTRP9 ameliorates atrial inflammation, fibrosis, and vulnerability to atrial fibrillation in post-myocardial infarction rats. J. Am. Heart Assoc..

[120] Niemann B., Li L., Siegler D., Siegler B.H., Knapp F., Hanna J., Aslam M., Kracht M., Schulz R., Rohrbach S. (2020). CTRP9 mediates protective effects in cardiomyocytes via ampk- and adiponectin receptor-mediated induction of anti-oxidant response. Cells.

[121] Jin Q., Su H., Yang R., Tan Y., Li B., Yi W., Dong Q., Zhang H., Xing W., Sun X. (2021). C1q/TNF-related protein-9 ameliorates hypoxia-induced pulmonary hypertension by regulating secretion of endothelin-1 and nitric oxide mediated by AMPK in rats. Sci. Rep..

[122] Zuo J., Yi C., Chen Z., Zhou B., Yang T., Lin J. (2022). A novel refined pyroptosis and inflammasome-related genes signature for predicting prognosis and immune microenvironment in pancreatic ductal adenocarcinoma. Sci. Rep..

[123] Chacko S.A., Sul J., Song Y., Li X., LeBlanc J., You Y., Butch A., Liu S. (2011). Magnesium supplementation, metabolic and inflammatory markers, and global genomic and proteomic profiling: A randomized, double-blind, controlled, crossover trial in overweight individuals. Am. J. Clin. Nutr..

[124] Wang W., Lau W.B., Wang Y., Ma X., Li R. (2016). Reduction of CTRP9, a novel anti-platelet adipokine, contributes to abnormal platelet activity in diabetic animals. Cardiovasc. Diabetol..

[125] Koh Y.W., Park C.S., Yoon D.H., Suh C., Huh J. (2014). CD163 expression was associated with angiogenesis and shortened survival in patients with uniformly treated classical Hodgkin lymphoma. PLoS ONE.

[126] Cepelova M., Kruseova J., Luks A., Capek V., Cepela P., Potockova J., Kraml P. (2019). Accelerated atherosclerosis, hyperlipoproteinemia and insulin resistance in long-term survivors of Hodgkin lymphoma during childhood and adolescence. Neoplasma.

[127] Bilora F., Pietrogrande F., Campagnolo E., Rossato A., Polato G., Pomerri F., Muzzio P.C. (2010). Are Hodgkin and non-Hodgkin patients at a greater risk of atherosclerosis? A follow-up of 3 years. Eur. J. Cancer Care.

[128] Gutierrez-Munoz C., Mendez-Barbero N., Svendsen P., Sastre C., Fernandez-Laso V., Quesada P., Egido J., Escola-Gil J.C., Martin-Ventura J.L., Moestrup S.K. (2020). CD163 deficiency increases foam cell formation and plaque progression in atherosclerotic mice. FASEB J..

[129] Akila P., Prashant V., Suma M.N., Prashant S.N., Chaitra T.R. (2012). CD163 and its expanding functional repertoire. Clin. Chim. Acta..

[130] Fu Y., Wu Y., Liu E. (2020). C-reactive protein and cardiovascular disease: From animal studies to the clinic (Review). Exp. Ther. Med..

[131] Gozal D., Kheirandish-Gozal L., Bhattacharjee R., Kim J. (2012). C-reactive protein and obstructive sleep apnea syndrome in children. Front. Biosci..

[132] Lin L.Y., Lee W.J., Shen H.N., Yang W.S., Pai N.H., Su T.C., Liau C.S. (2007). Nitric oxide production is paradoxically decreased after weight reduction surgery in morbid obesity patients. Atherosclerosis.

[133] Budassi S., Biccire F.G., Paoletti G., Marco V., Boi A., Romagnoli E., Fabbiocchi F., Fineschi M., Di Pietro R., Versaci F. (2022). The role of the association between serum C-reactive protein levels and coronary plaque macrophage accumulation in predicting clinical events—Results from the CLIMA registry. J. Cardiovasc. Transl. Res..

[134] Murad H.A.S., Rafeeq M.M., Alqurashi T.M.A. (2021). Role and implications of the CXCL12/CXCR4/CXCR7 axis in atherosclerosis: Still a debate. Ann. Med..

[135] LaRocca T.J., Altman P., Jarrah A.A., Gordon R., Wang E., Hadri L., Burke M.W., Haddad G.E., Hajjar R.J., Tarzami S.T. (2019). Cxcr4 cardiac specific knockout mice develop a progressive cardiomyopathy. Int. J. Mol. Sci..

[136] Livshits G., Kalinkovich A. (2019). Inflammaging as a common ground for the development and maintenance of sarcopenia, obesity, cardiomyopathy and dysbiosis. Ageing Res. Rev..

[137] Cai X., Chen Z., Pan X., Xia L., Chen P., Yang Y., Hu H., Zhang J., Li K., Ge J. (2014). Inhibition of angiogenesis, fibrosis and thrombosis by tetramethylpyrazine: Mechanisms contributing to the SDF-1/CXCR4 axis. PLoS ONE.

[138] Doring Y., Noels H., van der Vorst E.P.C., Neideck C., Egea V., Drechsler M., Mandl M., Pawig L., Jansen Y., Schroder K. (2017). Vascular CXCR4 limits atherosclerosis by maintaining arterial integrity: Evidence from mouse and human studies. Circulation..

[139] Meng Y., Zhang C., Liang L., Wei L., Wang H., Zhou F., Li R., Zou D., Huang X., Liu J. (2021). Identification of potential key genes involved in the carotid atherosclerosis. Clin. Interv. Aging..

[140] Kobayashi H., Takeno M., Saito T., Takeda Y., Kirino Y., Noyori K., Hayashi T., Ueda A., Ishigatsubo Y. (2006). Regulatory role of heme oxygenase 1 in inflammation of rheumatoid arthritis. Arthritis Rheum..

[141] Adawi M., Firas S., Blum A. (2019). Rheumatoid arthritis and atherosclerosis. Isr. Med. Assoc. J..

[142] Kishimoto Y., Kondo K., Momiyama Y. (2019). The protective role of heme oxygenase-1 in atherosclerotic diseases. Int. J. Mol. Sci..

[143] Grochot-Przeczek A., Lach R., Mis J., Skrzypek K., Gozdecka M., Sroczynska P., Dubiel M., Rutkowski A., Kozakowska M., Zagorska A. (2009). Heme oxygenase-1 accelerates cutaneous wound healing in mice. PLoS ONE.

[144] Wu D., Hu Q., Wang Y., Jin M., Tao Z., Wan J. (2022). Identification of HMOX1 as a critical ferroptosis-related gene in atherosclerosis. Front. Cardiovasc. Med..

[145] Bhattacharya R., Senbanerjee S., Lin Z., Mir S., Hamik A., Wang P., Mukherjee P., Mukhopadhyay D., Jain M.K. (2005). Inhibition of vascular permeability factor/vascular endothelial growth factor-mediated angiogenesis by the Kruppel-like factor KLF2. J. Biol. Chem..

[146] Turpaev K.T. (2020). Transcription factor KLF2 and its role in the regulation of inflammatory processes. Biochemistry.

[147] Zahlten J., Steinicke R., Opitz B., Eitel J., N’guessan P.D., Vinzing M., Witzenrath M., Schmeck B., Hammerschmidt S., Suttorp N. (2010). TLR2- and nucleotide-binding oligomerization domain 2-dependent Krüppel-like factor 2 expression downregulates NF-kappa B-related gene expression. J. Immunol..

[148] Manoharan P., Basford J.E., Pilcher-Roberts R., Neumann J., Hui D.Y., Lingrel J.B. (2014). Reduced levels of microRNAs miR-124a and miR-150 are associated with increased proinflammatory mediator expression in Kruppel-like factor 2 (KLF2)-deficient macrophages. J. Biol. Chem..

[149] Doddaballapur A., Michalik K.M., Manavski Y., Lucas T., Houtkooper R.H., You X., Chen W., Zeiher A.M., Potente M., Dimmeler S. (2015). Laminar shear stress inhibits endothelial cell metabolism via KLF2-mediated repression of PFKFB3. Arterioscler. Thromb. Vasc. Biol..

[150] Li L., Mou J., Han Y., Wang M., Lu S., Ma Q., Wang J., Ye J., Sun G. (2023). Calenduloside e modulates macrophage polarization via KLF2-regulated glycolysis, contributing to attenuates atherosclerosis. Int. Immunopharmacol..

[151] Petropoulou P.I., Berbee J.F., Theodoropoulos V., Hatziri A., Stamou P., Karavia E.A., Spyridonidis A., Karagiannides I., Kypreos K.E. (2015). Lack of LCAT reduces the LPS-neutralizing capacity of HDL and enhances LPS-induced inflammation in mice. Biochim. Biophys. Acta.

[152] Savel J., Lafitte M., Pucheu Y., Pradeau V., Tabarin A., Couffinhal T. (2012). Very low levels of HDL cholesterol and atherosclerosis, a variable relationship—A review of LCAT deficiency. Vasc. Health Risk Manag..

[153] Ozmen H.K., Askın S. (2013). Lecithin: Cholesterol acyltransferase and na(+)-k(+)-ATPase activity in patients with breast cancer. J. Breast Cancer.

[154] Yang K., Wang J., Xiang H., Ding P., Wu T., Ji G. (2022). LCAT-targeted therapies: Progress, failures and future. Biomed. Pharmacother..

[155] Sasaki M., Delawary M., Sakurai H., Kobayashi H., Nakao N., Tsuru H., Fukushima Y., Honzumi S., Moriyama S., Wada N. (2021). Novel LCAT (lecithin:cholesterol acyltransferase) activator DS-8190a prevents the progression of plaque accumulation in atherosclerosis models. Arterioscler. Thromb. Vasc. Biol..

[156] Berrougui H., Khalil A. (2009). Age-associated decrease of high-density lipoprotein-mediated reverse cholesterol transport activity. Rejuvenation Res..

[157] Valcarcel-Ares M.N., Gautam T., Warrington J.P., Bailey-Downs L., Sosnowska D., de Cabo R., Losonczy G., Sonntag W.E., Ungvari Z., Csiszar A. (2012). Disruption of Nrf2 signaling impairs angiogenic capacity of endothelial cells: Implications for microvascular aging. J. Gerontol. Biol. Sci. Med. Sci..

[158] Long M., Rojo de la Vega M., Wen Q., Bharara M., Jiang T., Zhang R., Zhou S., Wong P.K., Wondrak G.T., Zheng H. (2016). An essential role of NRF2 in diabetic wound healing. Diabetes.

[159] Gutierrez-Cuevas J., Galicia-Moreno M., Monroy-Ramirez H.C., Sandoval-Rodriguez A., Garcia-Banuelos J., Santos A., Armendariz-Borunda J. (2022). The role of NRF2 in obesity-associated cardiovascular risk factors. Antioxidants.

[160] Yu W., Liu W., Xie D., Wang Q., Xu C., Zhao H., Lv J., He F., Chen B., Yamamoto T. (2022). High level of uric acid promotes atherosclerosis by targeting NRF2-mediated autophagy dysfunction and ferroptosis. Oxid. Med. Cell. Longev..

[161] Kombairaju P., Kerr J.P., Roche J.A., Pratt S.J.P., Lovering R.M., Sussan T.E., Kim J.H., Shi G., Biswal S., Ward C.W. (2014). Genetic silencing of Nrf2 enhances X-ROS in dysferlin-deficient muscle. Front. Physiol..

[162] Batty M., Bennett M.R., Yu E. (2022). The role of oxidative stress in atherosclerosis. Cells.

[163] Zhao Z., Wang X., Zhang R., Ma B., Niu S., Di X., Ni L., Liu C. (2021). Melatonin attenuates smoking-induced atherosclerosis by activating the Nrf2 pathway via NLRP3 inflammasomes in endothelial cells. Aging.

[164] Shchelkunova T.A., Morozov I.A., Rubtsov P.M., Bobryshev Y.V., Sobenin I.A., Orekhov A.N., Andrianova I.V., Smirnov A.N. (2013). Lipid regulators during atherogenesis: Expression of LXR, PPAR, and SREBP mRNA in the human aorta. PLoS ONE.

[165] Zhao L., Lei W., Deng C., Wu Z., Sun M., Jin Z., Song Y., Yang Z., Jiang S., Shen M. (2021). The roles of liver X receptor α in inflammation and inflammation-associated diseases. J. Cell. Physiol..

[166] Bischoff E.D., Daige C.L., Petrowski M., Dedman H., Pattison J., Juliano J., Li A.C., Schulman I.G. (2010). Non-redundant roles for LXRalpha and LXRbeta in atherosclerosis susceptibility in low density lipoprotein receptor knockout mice. J. Lipid Res..

[167] Parikh M., Patel K., Soni S., Gandhi T. (2014). Liver X receptor: A cardinal target for atherosclerosis and beyond. J. Atheroscler. Thromb..

[168] Levin N., Bischoff E.D., Daige C.L., Thomas D., Vu C.T., Heyman R.A., Tangirala R.K., Schulman I.G. (2005). Macrophage liver X receptor is required for antiatherogenic activity of LXR agonists. Arterioscler. Thromb. Vasc. Biol..

[169] Aidoudi S., Bikfalvi A. (2010). Interaction of PF4 (CXCL4) with the vasculature: A role in atherosclerosis and angiogenesis. Thromb. Haemost..

[170] Sharpe R.J., Murphy G.F., Whitaker D., Galli S.J., Maione T.E. (1991). Induction of local inflammation by recombinant human platelet factor 4 in the mouse. Cell. Immunol..

[171] Meyer S.C., Steinmann E., Lehmann T., Muesser P., Passweg J.R., Skoda R.C., Tsakiris D.A. (2017). Anti-platelet factor 4/heparin antibody formation occurs endogenously and at unexpected high frequency in polycythemia vera. Biomed. Res. Int..

[172] Gleissner C.A., Shaked I., Erbel C., Böckler D., Katus H.A., Ley K. (2010). CXCL4 downregulates the atheroprotective hemoglobin receptor CD163 in human macrophages. Circ. Res..

[173] Brites F., Martin M., Guillas I., Kontush A. (2017). Antioxidative activity of high-density lipoprotein (HDL): Mechanistic insights into potential clinical benefit. BBA Clin..

[174] Mochly-Rosen D., Zakhari S. (2010). Focus on: The cardiovascular system: What did we learn from the French (Paradox)?. Alcohol. Res. Health.

[175] Ikhlef S., Berrougui H., Kamtchueng Simo O., Zerif E., Khalil A. (2017). Human paraoxonase 1 overexpression in mice stimulates HDL cholesterol efflux and reverse cholesterol transport. PLoS ONE.

[176] Aluganti Narasimhulu C., Mitra C., Bhardwaj D., Burge K.Y., Parthasarathy S. (2019). Alzheimer’s disease markers in aged ApoE-pon1 deficient mice. J. Alzheimers Dis..

[177] Varatharajalu R., Garige M., Leckey L.C., Gong M., Lakshman M.R. (2010). Betaine protects chronic alcohol and omega-3 PUFA-mediated down-regulations of PON1 gene, serum PON1 and homocysteine thiolactonase activities with restoration of liver GSH. Alcohol. Clin. Exp. Res..

[178] Ebert J., Wilgenbus P., Teiber J.F., Jurk K., Schwierczek K., Dohrmann M., Xia N., Li H., Spiecker L., Ruf W. (2018). Paraoxonase-2 regulates coagulation activation through endothelial tissue factor. Blood.

[179] Witte I., Altenhofer S., Wilgenbus P., Amort J., Clement A.M., Pautz A., Li H., Forstermann U., Horke S. (2011). Beyond reduction of atherosclerosis: PON2 provides apoptosis resistance and stabilizes tumor cells. Cell Death Dis..

[180] Gray S.P., Di Marco E., Kennedy K., Chew P., Okabe J., El-Osta A., Calkin A.C., Biessen E.A., Touyz R.M., Cooper M.E. (2016). Reactive oxygen species can provide atheroprotection via NOX4-dependent inhibition of inflammation and vascular remodeling. Arterioscler. Thromb. Vasc. Biol..

[181] Furlong C.E., Marsillach J., Jarvik G.P., Costa L.G. (2016). Paraoxonases-1, -2 and -3: What are their functions?. Chem. Biol. Interact..

[182] Shiner M., Fuhrman B., Aviram M. (2007). Macrophage paraoxonase 2 (PON2) expression is up-regulated by pomegranate juice phenolic anti-oxidants via PPAR gamma and AP-1 pathway activation. Atherosclerosis.

[183] Sagheer U., Gong J., Chung C. (2015). Pigment epithelium-derived factor (PEDF) is a determinant of stem cell fate: Lessons from an ultra-rare disease. J. Dev. Biol..

[184] Qi W., Yang C., Dai Z., Che D., Feng J., Mao Y., Cheng R., Wang Z., He X., Zhou T. (2015). High levels of pigment epithelium-derived factor in diabetes impair wound healing through suppression of Wnt signaling. Diabetes.

[185] Gluchowska A., Cysewski D., Baj-Krzyworzeka M., Szatanek R., Węglarczyk K., Podszywałow-Bartnicka P., Sunderland P., Kozłowska E., Sliwinska M.A., Dąbrowski M. (2022). Unbiased proteomic analysis of extracellular vesicles secreted by senescent human vascular smooth muscle cells reveals their ability to modulate immune cell functions. Geroscience.

[186] Ogata N., Tombran-Tink J., Nishikawa M., Nishimura T., Mitsuma Y., Sakamoto T., Matsumura M. (2001). Pigment epithelium-derived factor in the vitreous is low in diabetic retinopathy and high in rhegmatogenous retinal detachment. Am. J. Ophthalmol..

[187] He X., Cheng R., Benyajati S., Ma J.X. (2015). PEDF and its roles in physiological and pathological conditions: Implication in diabetic and hypoxia-induced angiogenic diseases. Clin. Sci..

[188] Wang H., Yang Y., Yang M., Li X., Tan J., Wu Y., Zhang Y., Li Y., Hu B., Deng S. (2019). Pigment epithelial-derived factor deficiency accelerates atherosclerosis development via promoting endothelial fatty acid uptake in mice with hyperlipidemia. J. Am. Heart Assoc..

[189] Cheng Q., Xia W., Yang S., Ye P., Mei M., Song Y., Luo M., Li Q. (2013). Association of serum pigment epithelium-derived factor with high-sensitivity C-reactive protein in women with polycystic ovary syndrome. J. Endocrinol. Invest..

[190] Principe D., DeCant B., Diaz A., Mangan R., Hwang R., Lowy A., Shetuni B., Sreekumar B., Chung C., Bentrem D. (2016). PEDF inhibits pancreatic tumorigenesis by attenuating the fibro-inflammatory reaction. Oncotarget.

[191] Wolf D., Ley K. (2019). Immunity and Inflammation in atherosclerosis. Circ. Res..

[192] Nogami H., Ono Y., Katoh R., Oohira A. (1993). Microvascular and cellular defects of the periosteum of osteogenesis imperfecta. Clin. Orthop. Relat. Res..

[193] Munns C.F., Rauch F., Zeitlin L., Fassier F., Glorieux F.H. (2004). Delayed osteotomy but not fracture healing in pediatric osteogenesis imperfecta patients receiving pamidronate. J. Bone Miner. Res..

[194] Salter L., Offiah A.C., Bishop N. (2018). Elevated platelet counts in a cohort of children with moderate-severe osteogenesis imperfecta suggest that inflammation is present. Arch. Dis. Child..

[195] Li Y., Shen S., Ding S., Wang L. (2018). Toll-like receptor 2 downregulates the cholesterol efflux by activating the nuclear factor-κB pathway in macrophages and may be a potential therapeutic target for the prevention of atherosclerosis. Exp. Ther. Med..

[196] Richard A.L., Siegel S.J., Erikson J., Weiser J.N. (2014). TLR2 signaling decreases transmission of Streptococcus pneumoniae by limiting bacterial shedding in an infant mouse Influenza A co-infection model. PLoS Pathog..

[197] Mullick A.E., Soldau K., Kiosses W.B., Bell T.A., Tobias P.S., Curtiss L.K. (2008). Increased endothelial expression of Toll-like receptor 2 at sites of disturbed blood flow exacerbates early atherogenic events. J. Exp. Med..

[198] Saber T., Veale D.J., Balogh E., McCormick J., NicAnUltaigh S., Connolly M., Fearon U. (2011). Toll-like receptor 2 induced angiogenesis and invasion is mediated through the Tie2 signalling pathway in rheumatoid arthritis. PLoS ONE.

[199] Huang B., Park D.W., Baek S.H. (2016). TRIF is a regulator of TLR2-induced foam cell formation. Mol. Med. Rep..

[200] Li M., Yan S., Dong H., Huang Z., Li D., Tang Y., Pan Y., Yang Z., Pan H., Chen G. (2022). Clinical assessment and molecular mechanism of the upregulation of Toll-like receptor 2 (TLR2) in myocardial infarction. BMC Cardiovasc. Disord..

[201] Simone V., Brunetti O., Lupo L., Testini M., Maiorano E., Simone M., Longo V., Rolfo C., Peeters M., Scarpa A. (2017). Targeting angiogenesis in biliary tract cancers: An open option. Int. J. Mol. Sci..

[202] Yuan R., Xin Q., Ma X., Yu M., Miao Y., Chen K., Cong W. (2023). Identification of a novel angiogenesis signalling circSCRG1/miR-1268b/NR4A1 pathway in atherosclerosis and the regulatory effects of TMP-PF in vitro. Molecules.

[203] Liu D., Lv H., Liu Q., Sun Y., Hou S., Zhang L., Yang M., Han B., Wang G., Wang X. (2019). Atheroprotective effects of methotrexate via the inhibition of YAP/TAZ under disturbed flow. J. Transl. Med..

[204] Coleman P.R., Lay A.J., Ting K.K., Zhao Y., Li J., Jarrah S., Vadas M.A., Gamble J.R. (2020). YAP and the RhoC regulator ARHGAP18, are required to mediate flow-dependent endothelial cell alignment. Cell Commun. Signal..

[205] Wu D.W., Lin P.L., Wang L., Huang C.C., Lee H. (2017). The YAP1/SIX2 axis is required for DDX3-mediated tumor aggressiveness and cetuximab resistance in KRAS-wild-type colorectal cancer. Theranostics.

[206] Fang S., Wan X., Zou X., Sun S., Hao X., Liang C., Zhang Z., Zhang F., Sun B., Li H. (2021). Arsenic trioxide induces macrophage autophagy and atheroprotection by regulating ROS-dependent TFEB nuclear translocation and AKT/mTOR pathway. Cell Death Dis..

[207] Yang Y., Ma Q., Li Z., Wang H., Zhang C., Liu Y., Li B., Wang Y., Cui Q., Xue F. (2021). Harmine alleviates atherogenesis by inhibiting disturbed flow-mediated endothelial activation via protein tyrosine phosphatase PTPN14 and YAP. Br. J. Pharmacol..

[208] Xie W., Xiao W., Tang K., Zhang L., Li Y. (2020). Yes-associated protein 1: Role and treatment prospects in orthopedic degenerative diseases. Front. Cell Dev. Biol..

[209] Shan R., Liu N., Yan Y., Liu B. (2021). Apoptosis, autophagy and atherosclerosis: Relationships and the role of Hsp27. Pharmacol. Res..

[210] Abecasis G., Auton A., Brooks L., DePristo M., Durbin R., Handsaker R., Kang H., Marth G., McVean G., 1000 Genomes Project Consortium (2012). An integrated map of genetic variation from 1.092 human genomes. Nature.

[211] Kasowski M., Grubert F., Heffelfinger C., Hariharan M., Asabere A., Waszak S., Habegger L., Rozowsky J., Shi M., Urban A. (2010). Variation in transcription factor binding among humans. Science.

[212] Chadaeva I., Ponomarenko P., Kozhemyakina R., Suslov V., Bogomolov A., Klimova N., Shikhevich S., Savinkova L., Oshchepkov D., Kolchanov N.A. (2021). Domestication explains two-thirds of differential-gene-expression variance between domestic and wild animals; the remaining one-third reflects intraspecific and interspecific variation. Animals.

[213] Oshchepkov D., Chadaeva I., Kozhemyakina R., Shikhevich S., Sharypova E., Savinkova L., Klimova N.V., Tsukanov A., Levitsky V.G., Markel A.L. (2022). Transcription factors as important regulators of changes in behavior through domestication of gray rats: Quantitative data from RNA sequencing. Int. J. Mol. Sci..

[214] Shikhevich S., Chadaeva I., Khandaev B., Kozhemyakina R., Zolotareva K., Kazachek A., Oshchepkov D., Bogomolov A., Klimova N.V., Ivanisenko V.A. (2023). Differentially expressed genes and molecular susceptibility to human age-related diseases. Int. J. Mol. Sci..

[215] Albert F.W., Somel M., Carneiro M., Aximu-Petri A., Halbwax M., Thalmann O., Blanco-Aguiar J.A., Plyusnina I.Z., Trut L., Villafuerte R. (2012). A comparison of brain gene expression levels in domesticated and wild animals. PLoS Genet..

[216] Sato D.X., Rafati N., Ring H., Younis S., Feng C., Blanco-Aguiar J.A., Rubin C.J., Villafuerte R., Hallbook F., Carneiro M. (2020). Brain transcriptomics of wild and domestic rabbits suggests that changes in dopamine signaling and ciliary function contributed to evolution of tameness. Genome Biol. Evol..

[217] Yang X., Zhang H., Shang J., Liu G., Xia T., Zhao C., Sun G., Dou H. (2018). Comparative analysis of the blood transcriptomes between wolves and dogs. Anim. Genet..

[218] Hekman J.P., Johnson J.L., Edwards W., Vladimirova A.V., Gulevich R.G., Ford A.L., Kharlamova A.V., Herbeck Y., Acland G.M., Raetzman L.T. (2018). Anterior pituitary transcriptome suggests differences in ACTH release in tame and aggressive foxes. Genes Genomes Genet..

[219] Long K., Mao K., Che T., Zhang J., Qiu W., Wang Y., Tang Q., Ma J., Li M., Li X. (2018). Transcriptome differences in frontal cortex between wild boar and domesticated pig. Anim. Sci. J..

[220] Yang Y., Adeola A.C., Xie H.B., Zhang Y.P. (2018). Genomic and transcriptomic analyses reveal selection of genes for puberty in Bama Xiang pigs. Zool. Res..

[221] Fallahshahroudi A., Lotvedt P., Belteky J., Altimiras J., Jensen P. (2019). Changes in pituitary gene expression may underlie multiple domesticated traits in chickens. Hered. Edinb..

[222] Belyaev D.K. (1979). The Wilhelmine E. Key 1978 invitational lecture. Destabilizing selection as a factor in domestication. J. Hered..

[223] Stajich J.E., Block D., Boulez K., Brenner S.E., Chervitz S.A., Dagdigian C., Fuellen G., Gilbert J.G., Korf I., Lapp H. (2002). The Bioperl toolkit: Perl modules for the life sciences. Genom. Res..

[224] Hahn S., Buratowski S., Sharp P., Guarente L. (1989). Yeast TATA-binding protein TFIID binds to TATA elements with both consensus and nonconsensus DNA sequences. Proc. Natl. Acad. Sci. USA.

[225] Karas H., Knuppel R., Schulz W., Sklenar H., Wingender E. (1996). Combining structural analysis of DNA with search routines for the detection of transcription regulatory elements. Comput. Appli. Biosci..

[226] Ponomarenko M., Ponomarenko J., Frolov A., Podkolodny N., Savinkova L., Kolchanov N., Overton G. (1999). Identification of sequence-dependent features correlating to activity of DNA sites interacting with proteins. Bioinformatics.

[227] Varzari A., Tudor E., Bodrug N., Corloteanu A., Axentii E., Deyneko I.V. (2018). Age-specific association of CCL5 gene polymorphism with pulmonary tuberculosis: A case-control study. Genet. Test. Mol. Biomark..

[228] Stelzer G., Rosen N., Plaschkes I., Zimmerman S., Twik M., Fishilevich S., Stein T.I., Nudel R., Lieder I., Mazor Y. (2016). The GeneCards suite: From gene data mining to disease genome sequence analyses. Curr. Protoc. Bioinform..

