# Supplementary material for: Candidate SNP Markers Significantly Altering the Affinity of TATA-Binding Protein for the Promoters of Human Hub Genes for Atherogenesis, Atherosclerosis and Atheroprotection
Source: Int J Mol Sci. 2023 May 19;24(10):9010. doi: 10.3390/ijms24109010 (PMC10219026; doi:10.3390/ijms24109010)
Supplement: Supplementary file 1 [file ijms-24-09010-s001.zip › 2)_IJMS-2369459_R1_Supplement_DL_12may23.pdf]

International Journal of
Molecular Sciences

Supplementary Materials

Candidate SNP Markers Significantly Altering the Affinity of TATA-Binding Protein for the Promoters of Human Hub Genes for Atherogenesis, Atherosclerosis and Atheroprotection

Anton Bogomolov[1], Sergey Filonov[1,2], Irina Chadaeva[1], Dmitry Rasskazov[1], Bato Khandaev[1,2], Karina Zolotareva[1,2], Anna Kazachek[1,2], Dmitry Oshchepkov[1], Vladimir A. Ivanisenko[1], Pavel Demenkov[1], Nikolay Podkolodnyy [1,3], Ekaterina Kondratyuk[1], Petr Ponomarenko[1], Olga Podkolodnaya[1], Zakhar Mustafin[1], Ludmila Savinkova[1], Nikolay Kolchanov[1,*], Natalya Tverdokhleb[1], Mikhail Ponomarenko[1]

[1]Institute of Cytology and Genetics, Novosibirsk 630090, Russia
[2] The Natural Sciences Department, Novosibirsk State University, Novosibirsk 630090, Russia
[3]Institute of Computational Mathematics and Mathematical Geophysics, 630090 Novosibirsk, Russia
[*]Correspondence: kol@bionet.nsc.ru. Tel.: +7 (383) 363-49-91 ext. 1234 (N.K.)

Abstract: Atherosclerosis is a systemic disease in which focal lesions in arteries promote the build-up of lipoproteins and cholesterol they are transporting. The development of atheromas (atherogenesis) narrows blood vessels, reduces the blood supply and leads to cardiovascular diseases. According to the World Health Organization, cardiovascular diseases are the leading cause of death, which has been especially boosted since the COVID-19 pandemic. There is a variety of contributors to atherosclerosis, including lifestyle factors and genetic predisposition. Antioxidant diets and recreational exercises act as atheroprotectors and slow down atherogenesis. The search for molecular markers associated with atherogenesis and atheroprotection for predictive, preventive and personalized medicine is the most promising direction for the study of atherosclerosis. We analyzed 1068 human genes associated with atherogenesis, atherosclerosis and atheroprotection. The hub genes regulating these processes were found to be the most ancient. *In silico* analysis of all 5112 SNPs in their promoters revealed 466 candidate SNP markers that significantly change the affinity of the TATA-binding protein (TBP) for these promoters. These molecular markers made us confident that natural selection acts against underexpression of the hub genes for atherogenesis, atherosclerosis and atheroprotection. At the same time, upregulation of the one for atheroprotection promotes human health.

Keywords: human; atherogenesis; atheroprotection; atherosclerosis; hub gene; promoter; TBP; TATA box; SNP; candidate SNP marker; gene expression change; natural selection; *in silico* verification

CONTENTS

Table S1. BLAST-based [106] phylostratigraphic age indices (PAIs) of 1068 human genes associated with atherogenesis, atheroprotection or atherosclerosis according to the NCBI Gene database [105].

Table S2. Effects of underexpression or overexpression of the hub genes for atherogenesis, atheroprotection and atherosclerosis to human health according to the current version of the PubMed database [81].

Section S1. Supplementary methods for DNA sequence analysis.

Table S3. Candidate SNP markers within the 90-bp proximal promoters of 16 human hub genes for atherogenesis, atheroprotection and atherosclerosis according to *in silico* analysis.

Table S4. Comparison of the effects of unidirectional changes (a) in the expression of the human hub genes for atherogenesis, atheroprotection and atherosclerosis on the activity of these processes and (b) in the expression of the corresponding animal homologous genes that are differentially expressed (DEG) during domestication on the microevolutionary events leading to domestic and wild animals.

Table S1. BLAST-based [106] phylostratigraphic age indices (PAIs) of 1068 human genes associated with atherogenesis, atheroprotection or atherosclerosis according to the NCBI Gene database.

**Human gene**		**The most recent common ancestor**		**Human gene**		**The most recent common ancestor**		**Human gene**		**The most recent common ancestor**	
#	*Symbol*	**Taxon**	**PAI**	#	*Symbol*	**Taxon**	**PAI**	#	*Symbol*	**Taxon**	**PAI**
(a) 16 human hub genes for atherogenesis, atheroprotection and atherosclerosis according to the NCBI Gene database [105]											
1	*APOA1*	Cellular organisms	0	7	*YAP1*	Opisthokonta	2	12	*NFE2L2*	Deuterostomia	6
2	*HMOX1*	Cellular organisms	0	8	*CD163*	Metazoa	3	13	*CXCR4*	Vertebrata	9
3	*PON1*	Cellular organisms	0	9	*CRP*	Eumetazoa	4	14	*TLR2*	Vertebrata	9
4	*PON2*	Cellular organisms	0	10	*KLF2*	Eumetazoa	4	15	*PF4*	Gnathostomata	10
5	*SERPINF1*	Cellular organisms	0	11	*NR1H3*	Bilateria	5	16	*C1QTNF9*	Euteleostomi	12
6	*LCAT*	Eukaryota	1								
the arithmetic mean (MEAN) ± standard error of the mean (SEM): 4.06 ± 1.02											
(b) 106 human hub genes for atherogenesis and atherosclerosis (not for atheroprotection) according to the NCBI Gene database [105]											
1	*APOB*	Cellular organisms	0	1. 37	*KLF5*	Eumetazoa	4	72	*CXCL1*	Gnathostomata	10
2	*ARG2*	Cellular organisms	0	2. 38	*RETN*	Eumetazoa	4	73	*F2R*	Gnathostomata	10
3	*GLO1*	Cellular organisms	0	3. 39	*SLC9A1*	Eumetazoa	4	74	*LCN2*	Gnathostomata	10
4	*HSPD1*	Cellular organisms	0	4. 40	*TNFSF11*	Eumetazoa	4	75	*TNF*	Gnathostomata	10
5	*MIF*	Cellular organisms	0	5. 41	*APOBR*	Bilateria	5	76	*ADIPOQ*	Euteleostomi	12
6	*NEIL3*	Cellular organisms	0	6. 42	*CD4*	Bilateria	5	77	*CCL2*	Euteleostomi	12
7	*NOS1*	Cellular organisms	0	7. 43	*F11R*	Bilateria	5	78	*CCL23*	Euteleostomi	12
8	*NOS3*	Cellular organisms	0	8. 44	*GSTA4*	Bilateria	5	79	*CCL5*	Euteleostomi	12
9	*NOX5*	Cellular organisms	0	9. 45	*HMGB1*	Bilateria	5	80	*CD59*	Euteleostomi	12
10	*PCSK9*	Cellular organisms	0	10. 46	*HSPB1*	Bilateria	5	81	*CDH5*	Euteleostomi	12
11	*PLA2G7*	Cellular organisms	0	11. 47	*ITGAV*	Bilateria	5	82	*CREB1*	Euteleostomi	12
12	*SAA1*	Cellular organisms	0	12. 48	*ITGB1*	Bilateria	5	83	*CX3CL1*	Euteleostomi	12
13	*SERPINE1*	Cellular organisms	0	13. 49	*KCNN4*	Bilateria	5	84	*CX3CR1*	Euteleostomi	12
14	*APOA4*	Eukaryota	1	14. 50	*LIPG*	Bilateria	5	85	*EBI3*	Euteleostomi	12
15	*CTSS*	Eukaryota	1	15. 51	*LPA*	Bilateria	5	86	*ELAVL1*	Euteleostomi	12
16	*LGMN*	Eukaryota	1	16. 52	*LPL*	Bilateria	5	87	*FABP4*	Euteleostomi	12
17	*MAPK1*	Eukaryota	1	17. 53	*MPO*	Bilateria	5	88	*FOXP3*	Euteleostomi	12
18	*MAPK3*	Eukaryota	1	18. 54	*NR4A1*	Bilateria	5	89	*GHRL*	Euteleostomi	12
19	*PRKAA1*	Eukaryota	1	19. 55	*NR4A*	Bilateria	5	90	*GJA4*	Euteleostomi	12
20	*RIPK1*	Eukaryota	1	20. 56	*NR4A3*	Bilateria	5	91	*IL18*	Euteleostomi	12
21	*UCP2*	Eukaryota	1	21. 57	*PALLD*	Bilateria	5	92	*IL6*	Euteleostomi	12
22	*APOH*	Opisthokonta	2	22. 58	*S100A8*	Bilateria	5	93	*LEP*	Euteleostomi	12
23	*NOX4*	Opisthokonta	2	23. 59	*S100A9*	Bilateria	5	94	*LEPR*	Euteleostomi	12
24	*PPIA*	Opisthokonta	2	24. 60	*TLR4*	Bilateria	5	95	*NTRK2*	Euteleostomi	12
25	*ABCA1*	Metazoa	3	25. 61	*INS*	Deuterostomia	6	96	*PAK1*	Euteleostomi	12
26	*AHR*	Metazoa	3	26. 62	*NR1I2*	Deuterostomia	6	97	*PLA2G2A*	Euteleostomi	12
27	*CAV1*	Metazoa	3	1. 63	*CD40*	Chordata	7	98	*PPARA*	Euteleostomi	12
28	*CD36*	Metazoa	3	1. 64	*MMP2*	Chordata	7	99	*PPARG*	Euteleostomi	12
29	*GPBP1*	Metazoa	3	2. 65	*CXCR2*	Vertebrata	9	100	*SORBS3*	Euteleostomi	12
30	*SCARB1*	Metazoa	3	3. 66	*EDN1*	Vertebrata	9	101	*CCR2*	Sarcopterygii	13
31	*SELE*	Metazoa	3	4. 67	*IGF2*	Vertebrata	9	102	*NLRP3*	Sarcopterygii	13
32	*SELP*	Metazoa	3	5. 68	*NPY*	Vertebrata	9	103	*CXCL16*	Tetrapoda	15
33	*TOR2A*	Metazoa	3	6. 69	*APOE*	Gnathostomata	10	104	*CD47*	Amniota	16
34	*BMP4*	Eumetazoa	4	7. 70	*BDNF*	Gnathostomata	10	105	*IL2RA*	Amniota	16
35	*C5*	Eumetazoa	4	8 . 71	*CD40LG*	Gnathostomata		106	*OLR1*	Amniota	16
36	*COMP*	Eumetazoa	4	10 9.							
the arithmetic mean (MEAN) ± standard error of the mean (SEM): 6.51 ± 0.16											
The arithmetic mean of the PAI index for the subset (a) of 16 hub genes for atherogenesis, atheroprotection, and atherosclerosis is statistically significantly less than that for the subset (b) of 106 hub genes for atherogenesis and atherosclerosis only (not for atheroprotection)							parametric Fisher's Z-test *Z*-score (significance):			2.08 ($p < 0.05$)	
							nonparametric Mann–Whitney test *U* value (significance):			575.50 ($p < 0.05$)	

Table S1 (continued).

Human gene		The most recent common ancestor		Human gene		The most recent common ancestor		Human gene		The most recent common ancestor	
#	*Symbol*	Taxon	PAI	#	*Symbol*	Taxon	PAI	#	*Symbol*	Taxon	PAI
(c) 21 human hub genes for atheroprotection and atherosclerosis (not for atherogenesis) according to the NCBI Gene database [105]											
1	*PON3*	Cellular organisms	0	8	*TIMP3*	Metazoa	3	15	*LIPC*	Bilateria	5
2	*HSPA5*	Eukaryota	1	9	*TNFAIP3*	Metazoa	3	16	*GPER1*	Deuterostomia	6
3	*SIRT1*	Eukaryota	1	10	*PTX3*	Eumetazoa	4	17	*APOA2*	Euteleostomi	12
4	*SOD1*	Eukaryota	1	11	*ESR1*	Bilateria	5	18	*APOA5*	Euteleostomi	12
5	*ANGPTL4*	Metazoa	3	12	*ESR2*	Bilateria	5	19	*CCR5*	Tetrapoda	15
6	*PLPP3*	Metazoa	3	13	*FASLG*	Bilateria	5	20	*IFNG*	Tetrapoda	15
7	*PNPLA3*	Metazoa	3	14	*HSPA1A*	Bilateria	5	21	*IL32*	Eutheria	19
the arithmetic mean (MEAN) ± standard error of the mean (SEM): 6.00 ± 1.16											
(d) 10 human genes specific for atheroprotection (not for atherogenesis or atherosclerosis) according to the NCBI Gene database [105]											
1	*RECK*	Cellular organisms	0	5	*CTTN*	Opisthokonta	2	8	*ID3*	Eumetazoa	4
2	*CAMKK2*	Eukaryota	1	6	*TM6SF2*	Opisthokonta	2	9	*HCAR1*	Gnathostomata	10
3	*PDCD4*	Eukaryota	1	7	*FSTL1*	Eumetazoa	4	10	*IFNB1*	Amniota	16
4	*PRKAA2*	Eukaryota	1								
the arithmetic mean (MEAN) ± standard error of the mean (SEM): 4.10 ± 1.06											
(e) 58 human genes specific for atherogenesis (not for atheroprotection or atherosclerosis) according to the NCBI Gene database [105]											
1	*CTH*	Cellular organisms	0	21	*LMCD1*	Metazoa	3	40	*CCL19*	Gnathostomata	10
2	*NOD1*	Cellular organisms	0	22	*MAPK14*	Metazoa	3	41	*F2RL1*	Gnathostomata	10
3	*RIGI*	Cellular organisms	0	23	*PRKCB*	Metazoa	3	42	*F2RL2*	Gnathostomata	10
4	*AIF1*	Eukaryota	1	24	*SGK1*	Metazoa	3	43	*ABI1*	Euteleostomi	12
5	*ATG16L1*	Eukaryota	1	25	*TRAF6*	Metazoa	3	44	*CCL7*	Euteleostomi	12
6	*CFAP91*	Eukaryota	1	26	*AKT1S1*	Eumetazoa	4	45	*COL12A1*	Euteleostomi	12
7	*CYLD*	Eukaryota	1	27	*CDKN1A*	Eumetazoa	4	46	*ENTPD1*	Euteleostomi	12
8	*GSK3B*	Eukaryota	1	28	*GPS2*	Eumetazoa	4	47	*HNRNPD*	Euteleostomi	12
9	*HDAC1*	Eukaryota	1	29	*ITGA5*	Eumetazoa	4	48	*HYAL1*	Euteleostomi	12
10	*HDAC2*	Eukaryota	1	30	*LPP*	Eumetazoa	4	49	*ITGAM*	Euteleostomi	12
11	*PFN1*	Eukaryota	1	31	*VTN*	Eumetazoa	4	50	*MYH9*	Euteleostomi	12
12	*PIK3CG*	Eukaryota	1	32	*GATA6*	Bilateria	5	51	*OSMR*	Euteleostomi	12
13	*PSME3*	Eukaryota	1	33	*PTGER4*	Bilateria	5	52	*PRLR*	Euteleostomi	12
14	*RAB7A*	Eukaryota	1	34	*SCARF1*	Bilateria	5	53	*RGCC*	Euteleostomi	12
15	*STARD5*	Eukaryota	1	35	*SLC7A5*	Bilateria	5	54	*IL7*	Sarcopterygii	13
16	*ABL1*	Opisthokonta	2	36	*CCN2*	Chordata	7	55	*CTSG*	Amniota	16
17	*SORL1*	Opisthokonta	2	37	*TLR9*	Chordata	7	56	*IL4*	Amniota	16
18	*TNFAIP8L1*	Opisthokonta	2	38	*TNFRSF11A*	Chordata	7	57	*OSCAR*	Amniota	16
19	*ETS1*	Metazoa	3	39	*NR1I3*	Vertebrata	9	58	*TSLP*	Eutheria	19
20	*IRF1*	Metazoa	3								
the arithmetic mean (MEAN) ± standard error of the mean (SEM): 6.12 ± 0.68											
(f) 856 human genes specific for atherosclerosis (not for atherogenesis or atheroprotection) according to the NCBI Gene database [105]											
1	*ACE*	Cellular organisms	0	15	*CAT*	Cellular organisms	0	29	*DDAH1*	Cellular organisms	0
2	*ACE2*	Cellular organisms	0	16	*CDKN1C*	Cellular organisms	0	30	*DDAH2*	Cellular organisms	0
3	*ACP5*	Cellular organisms	0	17	*CDKN2A*	Cellular organisms	0	31	*DHCR7*	Cellular organisms	0
4	*AGXT2*	Cellular organisms	0	18	*CDKN2B*	Cellular organisms	0	32	*DNMT1*	Cellular organisms	0
5	*ALDH2*	Cellular organisms	0	19	*CDKN3*	Cellular organisms	0	33	*EPHX2*	Cellular organisms	0
6	*ALDH4A1*	Cellular organisms	0	20	*CEMIP*	Cellular organisms	0	34	*F2*	Cellular organisms	0
7	*ALOX5AP*	Cellular organisms	0	21	*CILP2*	Cellular organisms	0	35	*FBXO3*	Cellular organisms	0
8	*ANKRD6*	Cellular organisms	0	22	*CMPK2*	Cellular organisms	0	36	*GCKR*	Cellular organisms	0
9	*APCDD1*	Cellular organisms	0	23	*COMT*	Cellular organisms	0	37	*GLS2*	Cellular organisms	0
10	*ARG1*	Cellular organisms	0	24	*CPS1*	Cellular organisms	0	38	*GNMT*	Cellular organisms	0
11	*ARSB*	Cellular organisms	0	25	*CRY1*	Cellular organisms	0	39	*GPT*	Cellular organisms	0
12	*BCO1*	Cellular organisms	0	26	*CTSC*	Cellular organisms	0	40	*GPX3*	Cellular organisms	0
13	*C4A*	Cellular organisms	0	27	*CYP19A1*	Cellular organisms	0	41	*HAS2*	Cellular organisms	0
14	*CARHSP1*	Cellular organisms	0	28	*CYP26B1*	Cellular organisms	0	42	*HPSE*	Cellular organisms	0

Table S1 (continued).

Human gene		The most recent common ancestor		Human gene		The most recent common ancestor		Human gene		The most recent common ancestor	
#	*Symbol*	Taxon	PAI	#	*Symbol*	Taxon	PAI	#	*Symbol*	Taxon	PAI
43	*IDO1*	Cellular organisms	0	96	*AOC3*	Eukaryota	1	149	*H6PD*	Eukaryota	1
44	*ISM1*	Cellular organisms	0	97	*APH1B*	Eukaryota	1	150	*HACD4*	Eukaryota	1
45	*ITLN1*	Cellular organisms	0	98	*AQP9*	Eukaryota	1	151	*HERPUD1*	Eukaryota	1
46	*LECT2*	Cellular organisms	0	99	*ARID5B*	Eukaryota	1	152	*HMGCR*	Eukaryota	1
47	*LRRC18*	Cellular organisms	0	100	*ATG7*	Eukaryota	1	153	*HSPA12B*	Eukaryota	1
48	*LRRIQ3*	Cellular organisms	0	101	*ATP6V1C2*	Eukaryota	1	154	*HSPA4*	Eukaryota	1
49	*LTA4H*	Cellular organisms	0	102	*AURKA*	Eukaryota	1	155	*IGFALS*	Eukaryota	1
50	*LTC4S*	Cellular organisms	0	103	*BPIFB4*	Eukaryota	1	156	*IKBKB*	Eukaryota	1
51	*MARCO*	Cellular organisms	0	104	*BRAP*	Eukaryota	1	157	*ILK*	Eukaryota	1
52	*MGMT*	Cellular organisms	0	105	*BRCA1*	Eukaryota	1	158	*KIF13B*	Eukaryota	1
53	*MTHFD2*	Cellular organisms	0	106	*CANX*	Eukaryota	1	159	*KL*	Eukaryota	1
54	*NAMPT*	Cellular organisms	0	107	*CAP1*	Eukaryota	1	160	*LIAS*	Eukaryota	1
55	*NAT2*	Cellular organisms	0	108	*CCNB2*	Eukaryota	1	161	*MAN2B1*	Eukaryota	1
56	*NAT8*	Cellular organisms	0	109	*CD248*	Eukaryota	1	162	*MAPK7*	Eukaryota	1
57	*NEU1*	Cellular organisms	0	110	*CD5L*	Eukaryota	1	163	*METTL14*	Eukaryota	1
58	*NGB*	Cellular organisms	0	111	*CDC20B*	Eukaryota	1	164	*METTL3*	Eukaryota	1
59	*NOS2*	Cellular organisms	0	112	*CDK5*	Eukaryota	1	165	*MLKL*	Eukaryota	1
60	*PAFAH1B2*	Cellular organisms	0	113	*CDK5RAP3*	Eukaryota	1	166	*MRAS*	Eukaryota	1
61	*PAFAH1B3*	Cellular organisms	0	114	*CDK9*	Eukaryota	1	167	*MTHFR*	Eukaryota	1
62	*PAPPA*	Cellular organisms	0	115	*CETP*	Eukaryota	1	168	*MTOR*	Eukaryota	1
63	*PARK7*	Cellular organisms	0	116	*CLTCL1*	Eukaryota	1	169	*MYO1E*	Eukaryota	1
64	*PDSS2*	Cellular organisms	0	117	*CNOT3*	Eukaryota	1	170	*MYO7B*	Eukaryota	1
65	*PEPD*	Cellular organisms	0	118	*COMMD10*	Eukaryota	1	171	*NAXE*	Eukaryota	1
66	*PRTFDC1*	Cellular organisms	0	119	*CP*	Eukaryota	1	172	*NKAPL*	Eukaryota	1
67	*PTGES*	Cellular organisms	0	120	*CPT1A*	Eukaryota	1	173	*NOX1*	Eukaryota	1
68	*PTGS2*	Cellular organisms	0	121	*CSRP1*	Eukaryota	1	174	*NPC1*	Eukaryota	1
69	*RELN*	Cellular organisms	0	122	*CTSB*	Eukaryota	1	175	*NPC1L1*	Eukaryota	1
70	*RSAD2*	Cellular organisms	0	123	*CTSD*	Eukaryota	1	176	*NUGGC*	Eukaryota	1
71	*SAA2*	Cellular organisms	0	124	*CTSK*	Eukaryota	1	177	*NUP98*	Eukaryota	1
72	*SAMD9*	Cellular organisms	0	125	*CTSL*	Eukaryota	1	178	*ORMDL3*	Eukaryota	1
73	*SDHB*	Cellular organisms	0	126	*CYBB*	Eukaryota	1	179	*OSBP2*	Eukaryota	1
74	*SERPIND1*	Cellular organisms	0	127	*CYP27A1*	Eukaryota	1	180	*OSBPL8*	Eukaryota	1
75	*SFTPD*	Cellular organisms	0	128	*CYP7B1*	Eukaryota	1	181	*PARP1*	Eukaryota	1
76	*SIRT6*	Cellular organisms	0	129	*DHX15*	Eukaryota	1	182	*PCMT1*	Eukaryota	1
77	*SLC5A7*	Cellular organisms	0	130	*DHX38*	Eukaryota	1	183	*PCYOX1*	Eukaryota	1
78	*SMPD3*	Cellular organisms	0	131	*DICER1*	Eukaryota	1	184	*PIEZO1*	Eukaryota	1
79	*SOD2*	Cellular organisms	0	132	*DNAH5*	Eukaryota	1	185	*PIK3CB*	Eukaryota	1
80	*SUB1*	Cellular organisms	0	133	*DOT1L*	Eukaryota	1	186	*PIWIL1*	Eukaryota	1
81	*TNKS*	Cellular organisms	0	134	*EEF2K*	Eukaryota	1	187	*PLA2G6*	Eukaryota	1
82	*TSPO*	Cellular organisms	0	135	*ERCC1*	Eukaryota	1	188	*PLEKHO1*	Eukaryota	1
83	*TTR*	Cellular organisms	0	136	*ERN1*	Eukaryota	1	189	*PLK1*	Eukaryota	1
84	*ZMPSTE24*	Cellular organisms	0	137	*FADS1*	Eukaryota	1	190	*PLTP*	Eukaryota	1
85	*AAK1*	Eukaryota	1	138	*FADS2*	Eukaryota	1	191	*PPM1K*	Eukaryota	1
86	*ABCB1*	Eukaryota	1	139	*FADS3*	Eukaryota	1	192	*PRDX1*	Eukaryota	1
87	*ABCD1*	Eukaryota	1	140	*FOLR2*	Eukaryota	1	193	*PRMT2*	Eukaryota	1
88	*ABCG5*	Eukaryota	1	141	*G6PC2*	Eukaryota	1	194	*PROS1*	Eukaryota	1
89	*ABCG8*	Eukaryota	1	142	*GAS6*	Eukaryota	1	195	*PSMA6*	Eukaryota	1
90	*ADIPOR1*	Eukaryota	1	143	*GCLC*	Eukaryota	1	196	*PSMD6*	Eukaryota	1
91	*ADIPOR2*	Eukaryota	1	144	*GGT1*	Eukaryota	1	197	*PTEN*	Eukaryota	1
92	*ADTRP*	Eukaryota	1	145	*GLTPD2*	Eukaryota	1	198	*PTGES3*	Eukaryota	1
93	*AIRE*	Eukaryota	1	146	*GRN*	Eukaryota	1	199	*PTGIS*	Eukaryota	1
94	*AKR1B1*	Eukaryota	1	147	*GSTP1*	Eukaryota	1	200	*QSOX1*	Eukaryota	1
95	*AKR1B10*	Eukaryota	1	148	*GTF2E2*	Eukaryota	1	201	*RAP1A*	Eukaryota	1

Table S1 (continued).

Human gene		The most recent common ancestor		Human gene		The most recent common ancestor		Human gene		The most recent common ancestor	
#	*Symbol*	Taxon	PAI	#	*Symbol*	Taxon	PAI	#	*Symbol*	Taxon	PAI
202	*RAP1B*	Eukaryota	1	255	*DPP4*	Opisthokonta	2	308	*CPE*	Metazoa	3
203	*RHBDF2*	Eukaryota	1	256	*ELOVL2*	Opisthokonta	2	309	*CREB3*	Metazoa	3
204	*RIPK2*	Eukaryota	1	257	*F12*	Opisthokonta	2	310	*CREB3L3*	Metazoa	3
205	*RNF111*	Eukaryota	1	258	*FRS2*	Opisthokonta	2	311	*CTNNB1*	Metazoa	3
206	*RNF213*	Eukaryota	1	259	*GALNT2*	Opisthokonta	2	312	*CYP11B2*	Metazoa	3
207	*RREB1*	Eukaryota	1	260	*JAK2*	Opisthokonta	2	313	*CYP27C1*	Metazoa	3
208	*RSPO2*	Eukaryota	1	261	*JUN*	Opisthokonta	2	314	*DAP*	Metazoa	3
209	*SELPLG*	Eukaryota	1	262	*MGAT5B*	Opisthokonta	2	315	*DGKH*	Metazoa	3
210	*SIRT3*	Eukaryota	1	263	*MYO5A*	Opisthokonta	2	316	*DLL4*	Metazoa	3
211	*SLC25A1*	Eukaryota	1	264	*NCF1*	Opisthokonta	2	317	*DOCK7*	Metazoa	3
212	*SLC44A3*	Eukaryota	1	265	*NF2*	Opisthokonta	2	318	*EGF*	Metazoa	3
213	*SOAT1*	Eukaryota	1	266	*OSBPL1A*	Opisthokonta	2	319	*ENO1*	Metazoa	3
214	*SPPL2A*	Eukaryota	1	267	*PLAU*	Opisthokonta	2	320	*ETS2*	Metazoa	3
215	*SPPL2B*	Eukaryota	1	268	*PLCG1*	Opisthokonta	2	321	*EYS*	Metazoa	3
216	*SQSTM1*	Eukaryota	1	269	*PPP1R3B*	Opisthokonta	2	322	*FOXA3*	Metazoa	3
217	*ST6GAL1*	Eukaryota	1	270	*PRKCE*	Opisthokonta	2	323	*FOXC2*	Metazoa	3
218	*STARD7*	Eukaryota	1	271	*PRKCZ*	Opisthokonta	2	324	*FOXO1*	Metazoa	3
219	*STK11*	Eukaryota	1	272	*PROM1*	Opisthokonta	2	325	*FOXO3*	Metazoa	3
220	*SYVN1*	Eukaryota	1	273	*PTPN2*	Opisthokonta	2	326	*FOXO4*	Metazoa	3
221	*TAT*	Eukaryota	1	274	*PTPN22*	Opisthokonta	2	327	*GDF15*	Metazoa	3
222	*TERT*	Eukaryota	1	275	*PTPN6*	Opisthokonta	2	328	*GNAI2*	Metazoa	3
223	*THBD*	Eukaryota	1	276	*ROCK1*	Opisthokonta	2	329	*GNB3*	Metazoa	3
224	*THOC5*	Eukaryota	1	277	*ROCK2*	Opisthokonta	2	330	*GPR26*	Metazoa	3
225	*TLN1*	Eukaryota	1	278	*SELENOS*	Opisthokonta	2	331	*GRK6*	Metazoa	3
226	*TLN2*	Eukaryota	1	279	*SELL*	Opisthokonta	2	332	*GSC2*	Metazoa	3
227	*TMCO1*	Eukaryota	1	280	*SHB*	Opisthokonta	2	333	*GSTO1*	Metazoa	3
228	*TMEM258*	Eukaryota	1	281	*SNX10*	Opisthokonta	2	334	*HGF*	Metazoa	3
229	*TNFRSF25*	Eukaryota	1	282	*SORCS1*	Opisthokonta	2	335	*HNF1A*	Metazoa	3
230	*TRAM1*	Eukaryota	1	283	*SORT1*	Opisthokonta	2	336	*HSPG2*	Metazoa	3
231	*TRIB3*	Eukaryota	1	284	*SREBF1*	Opisthokonta	2	337	*ICA1*	Metazoa	3
232	*TXN*	Eukaryota	1	285	*SREBF2*	Opisthokonta	2	338	*IGF1R*	Metazoa	3
233	*TXNL4B*	Eukaryota	1	286	*SYK*	Opisthokonta	2	339	*IKBKE*	Metazoa	3
234	*TXNRD1*	Eukaryota	1	287	*TERF2IP*	Opisthokonta	2	340	*IRS1*	Metazoa	3
235	*TXNRD2*	Eukaryota	1	288	*TGFB1I1*	Opisthokonta	2	341	*IRS2*	Metazoa	3
236	*UBE2I*	Eukaryota	1	289	*TNFRSF9*	Opisthokonta	2	342	*ITGA4*	Metazoa	3
237	*UCP1*	Eukaryota	1	290	*TRHDE*	Opisthokonta	2	343	*JAG1*	Metazoa	3
238	*USP24*	Eukaryota	1	291	*TUBB4A*	Opisthokonta	2	344	*KLF3*	Metazoa	3
239	*UTP20*	Eukaryota	1	292	*USF1*	Opisthokonta	2	345	*LAMC1*	Metazoa	3
240	*VKORC1*	Eukaryota	1	293	*VAMP3*	Opisthokonta	2	346	*LGALS3BP*	Metazoa	3
241	*WDFY4*	Eukaryota	1	294	*VCL*	Opisthokonta	2	347	*LRP6*	Metazoa	3
242	*XBP1*	Eukaryota	1	295	*AGT*	Metazoa	3	348	*MCAM*	Metazoa	3
243	*XDH*	Eukaryota	1	296	*ALMS1*	Metazoa	3	349	*MERTK*	Metazoa	3
244	*YWHAZ*	Eukaryota	1	297	*ANGPTL3*	Metazoa	3	350	*MSR1*	Metazoa	3
245	*ZBTB20*	Eukaryota	1	298	*ASXL2*	Metazoa	3	351	*MTNR1B*	Metazoa	3
246	*ZC3HC1*	Eukaryota	1	299	*AXL*	Metazoa	3	352	*MYLIP*	Metazoa	3
247	*ZPR1*	Eukaryota	1	300	*C1QTNF12*	Metazoa	3	353	*MYLK*	Metazoa	3
248	*ABCC6*	Opisthokonta	2	301	*CAPN10*	Metazoa	3	354	*NFKB1*	Metazoa	3
249	*ADAM10*	Opisthokonta	2	302	*CASP3*	Metazoa	3	355	*NOS1AP*	Metazoa	3
250	*ADAM17*	Opisthokonta	2	303	*CDKN1B*	Metazoa	3	356	*NOTCH1*	Metazoa	3
251	*BNC2*	Opisthokonta	2	304	*CELSR1*	Metazoa	3	357	*NOTCH2*	Metazoa	3
252	*CDC42*	Opisthokonta	2	305	*CELSR2*	Metazoa	3	358	*NOTCH3*	Metazoa	3
253	*CYBA*	Opisthokonta	2	306	*CFH*	Metazoa	3	359	*NOTCH4*	Metazoa	3
254	*CYP4V2*	Opisthokonta	2	307	*COL4A2*	Metazoa	3	360	*NPM1*	Metazoa	3

Table S1 (continued).

Human gene		The most recent common ancestor		Human gene		The most recent common ancestor		Human gene		The most recent common ancestor	
#	*Symbol*	Taxon	PAI	#	*Symbol*	Taxon	PAI	#	*Symbol*	Taxon	PAI
361	*NRG4*	Metazoa	3	414	*FNDC5*	Eumetazoa	4	467	*CCDC178*	Bilateria	5
362	*PCOLCE2*	Metazoa	3	415	*GATA2*	Eumetazoa	4	468	*CCL28*	Bilateria	5
363	*PCSK6*	Metazoa	3	416	*GDF11*	Eumetazoa	4	469	*CD86*	Bilateria	5
364	*PDGFC*	Metazoa	3	417	*GDF2*	Eumetazoa	4	470	*CEL*	Bilateria	5
365	*PXDN*	Metazoa	3	418	*GREM1*	Eumetazoa	4	471	*CERS5*	Bilateria	5
366	*RCN2*	Metazoa	3	419	*HERC6*	Eumetazoa	4	472	*COL18A1*	Bilateria	5
367	*RHOA*	Metazoa	3	420	*HIF1A*	Eumetazoa	4	473	*COL5A1*	Bilateria	5
368	*RYR3*	Metazoa	3	421	*HOMER1*	Eumetazoa	4	474	*CORIN*	Bilateria	5
369	*SCARB2*	Metazoa	3	422	*HOMER2*	Eumetazoa	4	475	*CRISP2*	Bilateria	5
370	*SCD*	Metazoa	3	423	*HOXA1*	Eumetazoa	4	476	*DCN*	Bilateria	5
371	*SLCO5A1*	Metazoa	3	424	*KLF4*	Eumetazoa	4	477	*EDNRA*	Bilateria	5
372	*SMAD7*	Metazoa	3	425	*LMNA*	Eumetazoa	4	478	*EDNRB*	Bilateria	5
373	*SNAI1*	Metazoa	3	426	*METRNL*	Eumetazoa	4	479	*EFEMP1*	Bilateria	5
374	*SOX18*	Metazoa	3	427	*MSTN*	Eumetazoa	4	480	*ELN*	Bilateria	5
375	*SOX4*	Metazoa	3	428	*MTUS1*	Eumetazoa	4	481	*ENG*	Bilateria	5
376	*SP1*	Metazoa	3	429	*NINJ2*	Eumetazoa	4	482	*EZH2*	Bilateria	1
377	*SRY*	Metazoa	3	430	*NTN1*	Eumetazoa	4	483	*FABP2*	Bilateria	1
378	*STRN3*	Metazoa	3	431	*NTNG1*	Eumetazoa	4	484	*FBLIM1*	Bilateria	1
379	*SVEP1*	Metazoa	3	432	*PDGFD*	Eumetazoa	4	485	*FLT1*	Bilateria	1
380	*TBX21*	Metazoa	3	433	*PLA2G3*	Eumetazoa	4	486	*FURIN*	Bilateria	1
381	*TBXAS1*	Metazoa	3	434	*PPP1R12A*	Eumetazoa	4	487	*GHSR*	Bilateria	1
382	*TET2*	Metazoa	3	435	*PTPRC*	Eumetazoa	4	488	*GPR146*	Bilateria	1
383	*TFPI*	Metazoa	3	436	*RELA*	Eumetazoa	4	489	*HDAC5*	Bilateria	1
384	*TFPI2*	Metazoa	3	437	*RETNLB*	Eumetazoa	4	490	*HOXA5*	Bilateria	1
385	*TNFAIP6*	Metazoa	3	438	*SELENOP*	Eumetazoa	4	491	*HOXC6*	Bilateria	1
386	*TRAF2*	Metazoa	3	439	*SESN2*	Eumetazoa	4	492	*HSPA8*	Bilateria	1
387	*TXNIP*	Metazoa	3	440	*SH2B3*	Eumetazoa	4	493	*ICAM1*	Bilateria	1
388	*TYRO3*	Metazoa	3	441	*SOCS3*	Eumetazoa	4	494	*IRF7*	Bilateria	1
389	*VWF*	Metazoa	3	442	*SOST*	Eumetazoa	4	495	*ITGA2B*	Bilateria	1
390	*ABCG1*	Eumetazoa	4	443	*TIMP1*	Eumetazoa	4	496	*ITGB2*	Bilateria	1
391	*ADAMTS13*	Eumetazoa	4	444	*TNFSF10*	Eumetazoa	4	497	*ITGB4*	Bilateria	1
392	*ADAMTS7*	Eumetazoa	4	445	*TNFSF12*	Eumetazoa	4	498	*KDR*	Bilateria	1
393	*AFF3*	Eumetazoa	4	446	*TNFSF13B*	Eumetazoa	4	499	*KISS1R*	Bilateria	1
394	*AHRR*	Eumetazoa	4	447	*TSPAN2*	Eumetazoa	4	500	*KLKB1*	Bilateria	1
395	*AMH*	Eumetazoa	4	448	*TSPAN4*	Eumetazoa	4	501	*LDB2*	Bilateria	1
396	*ANXA1*	Eumetazoa	4	449	*WNT4*	Eumetazoa	4	502	*LDLR*	Bilateria	1
397	*APCS*	Eumetazoa	4	450	*WNT5A*	Eumetazoa	4	503	*LGALS1*	Bilateria	1
398	*ATF3*	Eumetazoa	4	451	*ACTA2*	Bilateria	5	504	*LGALS12*	Bilateria	1
399	*BCL2*	Eumetazoa	4	452	*ADAMTS3*	Bilateria	5	505	*LGALS2*	Bilateria	
400	*BMP2*	Eumetazoa	4	453	*ADRB2*	Bilateria	5	506	*LGALS3*	Bilateria	
401	*C2*	Eumetazoa	4	454	*ADRB3*	Bilateria	5	507	*LRP1*	Bilateria	
402	*C3*	Eumetazoa	4	455	*AGER*	Bilateria	5	508	*LRP2*	Bilateria	
403	*CA12*	Eumetazoa	4	456	*ANGPTL2*	Bilateria	5	509	*MAFF*	Bilateria	
404	*CD9*	Eumetazoa	4	457	*AP1B1*	Bilateria	5	510	*MATR3*	Bilateria	
405	*CHI3L1*	Eumetazoa	4	458	*AQP1*	Bilateria	5	511	*MIA3*	Bilateria	5
406	*CHIT1*	Eumetazoa	4	459	*AVP*	Bilateria	5	512	*MMP14*	Bilateria	5
407	*CSGALNACT2*	Eumetazoa	4	460	*B2M*	Bilateria	5	513	*MTTP*	Bilateria	5
408	*CTNNA3*	Eumetazoa	4	461	*BAMBI*	Bilateria	5	514	*NALF1*	Bilateria	5
409	*DKK3*	Eumetazoa	4	462	*BGN*	Bilateria	5	515	*NCEH1*	Bilateria	5
410	*DNMT3A*	Eumetazoa	4	463	*BSG*	Bilateria	5	516	*NEXN*	Bilateria	5
411	*DOCK4*	Eumetazoa	4	464	*C1QTNF3*	Bilateria	5	517	*NFAT5*	Bilateria	5
412	*ECE1*	Eumetazoa	4	465	*CACNA2D1*	Bilateria	5	518	*NR1D1*	Bilateria	5
413	*ELANE*	Eumetazoa	4	466	*CADPS*	Bilateria	5	519	*NR1H4*	Bilateria	5

Table S1 (continued).

Human gene		The most recent common ancestor		Human gene		The most recent common ancestor		Human gene		The most recent common ancestor	
#	*Symbol*	Taxon	PAI	#	*Symbol*	Taxon	PAI	#	*Symbol*	Taxon	PAI
520	*NUMB*	Bilateria	5	573	*VDR*	Deuterostomia	6	626	*IGF1*	Vertebrata	9
521	*OGN*	Bilateria	5	574	*VEGFC*	Deuterostomia	6	627	*MBL2*	Vertebrata	9
522	*P2RX7*	Bilateria	5	575	*ADAMTS1*	Chordata	7	628	*NR3C1*	Vertebrata	9
523	*PECAM1*	Bilateria	5	576	*ADAMTS5*	Chordata	7	629	*NR3C2*	Vertebrata	9
524	*PGLYRP1*	Bilateria	5	577	*ANGPT2*	Chordata	7	630	*PROC*	Vertebrata	9
525	*PIGR*	Bilateria	5	578	*APLNR*	Chordata	7	631	*PROZ*	Vertebrata	9
526	*PLA2G10*	Bilateria	5	579	*BRINP3*	Chordata	7	632	*RARB*	Vertebrata	9
527	*PLG*	Bilateria	5	580	*CARD8*	Chordata	7	633	*RHO*	Vertebrata	9
528	*PLIN2*	Bilateria	5	581	*CNR1*	Chordata	7	634	*TSHB*	Vertebrata	9
529	*PPARGC1A*	Bilateria	5	582	*F5*	Chordata	7	635	*VIM*	Vertebrata	9
530	*PTGDS*	Bilateria	5	583	*F8*	Chordata	7	636	*ABO*	Gnathostomata	10
531	*RBP4*	Bilateria	5	584	*FGF19*	Chordata	7	637	*AGTR1*	Gnathostomata	10
532	*ROBO4*	Bilateria	5	585	*FGF21*	Chordata	7	638	*AGTR2*	Gnathostomata	10
533	*RORA*	Bilateria	5	586	*FGF23*	Chordata	7	639	*AHSG*	Gnathostomata	10
534	*RTN3*	Bilateria	5	587	*FN1*	Chordata	7	640	*ANXA5*	Gnathostomata	10
535	*S100A12*	Bilateria	5	588	*GSTM1*	Chordata	7	641	*BDKRB1*	Gnathostomata	10
536	*SDC1*	Bilateria	5	589	*HABP2*	Chordata	7	642	*CD28*	Gnathostomata	10
537	*SGMS2*	Bilateria	5	590	*HPR*	Chordata	7	643	*CELA2A*	Gnathostomata	10
538	*SHBG*	Bilateria	5	591	*IGFBP5*	Chordata	7	644	*CXCL12*	Gnathostomata	10
539	*SLC1A4*	Bilateria	5	592	*IL17A*	Chordata	7	645	*CXCL13*	Gnathostomata	10
540	*SLC5A10*	Bilateria	5	593	*ITGB3*	Chordata	7	646	*CXCL8*	Gnathostomata	10
541	*SLC6A4*	Bilateria	5	594	*ITGB5*	Chordata	7	647	*CYSLTR1*	Gnathostomata	10
542	*SMAD1*	Bilateria	5	595	*JAK1*	Chordata	7	648	*EVA1A*	Gnathostomata	10
543	*SMAD5*	Bilateria	5	596	*JAM3*	Chordata	7	649	*FABP3*	Gnathostomata	10
544	*SOCS1*	Bilateria	5	597	*KCNJ5*	Chordata	7	650	*FABP5*	Gnathostomata	10
545	*TGFB1*	Bilateria	5	598	*KCNMB1*	Chordata	7	651	*FETUB*	Gnathostomata	10
546	*THBS2*	Bilateria	5	599	*LTA*	Chordata	7	652	*GCG*	Gnathostomata	10
547	*TNFRSF11B*	Bilateria	5	600	*LTBR*	Chordata	7	653	*GPR18*	Gnathostomata	10
548	*TNFSF13*	Bilateria	5	601	*MAZ*	Chordata	7	654	*GPR55*	Gnathostomata	10
549	*TUSC1*	Bilateria	5	602	*MDK*	Chordata	7	655	*HCAR2*	Gnathostomata	10
550	*UNC5B*	Bilateria	5	603	*MMP9*	Chordata	7	656	*HGFAC*	Gnathostomata	10
551	*UTS2R*	Bilateria	5	604	*PGF*	Chordata	7	657	*IL1B*	Gnathostomata	10
552	*VCAM1*	Bilateria	5	605	*SLC12A1*	Chordata	7	658	*MEF2C*	Gnathostomata	10
553	*VLDLR*	Bilateria	5	606	*SPRYD4*	Chordata	7	659	*NGF*	Gnathostomata	10
554	*ZEB2*	Bilateria	5	607	*STC2*	Chordata	7	660	*P2RY2*	Gnathostomata	10
555	*ZFHX3*	Bilateria	5	608	*TGFBI*	Chordata	7	661	*PLAT*	Gnathostomata	10
556	*ADAM15*	Deuterostomia	6	609	*TGM2*	Chordata	7	662	*PPBP*	Gnathostomata	10
557	*ADAM33*	Deuterostomia	6	610	*TNC*	Chordata	7	663	*PTH*	Gnathostomata	10
558	*ADAMTS4*	Deuterostomia	6	611	*TNFRSF14*	Chordata	7	664	*RGS5*	Gnathostomata	10
559	*AKT3*	Deuterostomia	6	612	*TNFSF15*	Chordata	7	665	*SUCNR1*	Gnathostomata	10
560	*C1QTNF1*	Deuterostomia	6	613	*VEGFA*	Chordata	7	666	*TM4SF5*	Gnathostomata	10
561	*CASR*	Deuterostomia	6	614	*ACKR3*	Vertebrata	9	667	*TP53*	Gnathostomata	10
562	*CD44*	Deuterostomia	6	615	*ADM*	Vertebrata	9	668	*TPD52*	Gnathostomata	10
563	*CPB2*	Deuterostomia	6	616	*ALB*	Vertebrata	9	669	*ABI2*	Euteleostomi	12
564	*CST3*	Deuterostomia	6	617	*AR*	Vertebrata	9	670	*ACSL1*	Euteleostomi	12
565	*DKK1*	Deuterostomia	6	618	*C1QC*	Vertebrata	9	671	*ADAM8*	Euteleostomi	12
566	*HBEGF*	Deuterostomia	6	619	*CA2*	Vertebrata	9	672	*ADAM9*	Euteleostomi	12
567	*LTB4R*	Deuterostomia	6	620	*CXCR3*	Vertebrata	9	673	*ADGRL2*	Euteleostomi	12
568	*RUNX3*	Deuterostomia	6	621	*CXCR5*	Vertebrata	9	674	*AKT1*	Euteleostomi	12
569	*ST8SIA1*	Deuterostomia	6	622	*EDN3*	Vertebrata	9	675	*ALOX12*	Euteleostomi	12
570	*TLR7*	Deuterostomia	6	623	*F7*	Vertebrata	9	676	*ALOX15*	Euteleostomi	12
571	*TNFRSF18*	Deuterostomia	6	624	*GC*	Vertebrata	9	677	*ALOX5*	Euteleostomi	12
572	*TNFRSF1A*	Deuterostomia	6	625	*HP*	Vertebrata	9	678	*ANG*	Euteleostomi	12

Table S1 (continued).

Human gene		The most recent common ancestor		Human gene		The most recent common ancestor		Human gene		The most recent common ancestor	
#	*Symbol*	Taxon	PAI	#	*Symbol*	Taxon	PAI	#	*Symbol*	Taxon	PAI
679	*ANGPT1*	Euteleostomi	12	732	*HDAC9*	Euteleostomi	12	785	*PHACTR1*	Euteleostomi	12
680	*ANGPTL8*	Euteleostomi	12	733	*HNRNPC*	Euteleostomi	12	786	*PLAUR*	Euteleostomi	12
681	*APLN*	Euteleostomi	12	734	*IGFBP1*	Euteleostomi	12	787	*PPARD*	Euteleostomi	12
682	*APOC1*	Euteleostomi	12	735	*IGFBP3*	Euteleostomi	12	788	*RARRES2*	Euteleostomi	12
683	*APOC2*	Euteleostomi	12	736	*IL10*	Euteleostomi	12	789	*RFPL1*	Euteleostomi	12
684	*APP*	Euteleostomi	12	737	*IL12A*	Euteleostomi	12	790	*RTN4*	Euteleostomi	12
685	*BGLAP*	Euteleostomi	12	738	*IL1A*	Euteleostomi	12	791	*SAMD1*	Euteleostomi	12
686	*CACNA1C*	Euteleostomi	12	739	*IL1RN*	Euteleostomi	12	792	*SEMA3E*	Euteleostomi	12
687	*CALCA*	Euteleostomi	12	740	*IL20*	Euteleostomi	12	793	*SEMA7A*	Euteleostomi	12
688	*CAMK2D*	Euteleostomi	12	741	*IL21*	Euteleostomi	12	794	*SERPINA1*	Euteleostomi	12
689	*CAMP*	Euteleostomi	12	742	*IL22*	Euteleostomi	12	795	*SLAMF7*	Euteleostomi	12
690	*CAPG*	Euteleostomi	12	743	*IL23R*	Euteleostomi	12	796	*SOX6*	Euteleostomi	12
691	*CCL11*	Euteleostomi	12	744	*IL37*	Euteleostomi	12	797	*SPRY1*	Euteleostomi	12
692	*CCL17*	Euteleostomi	12	745	*IL6R*	Euteleostomi	12	798	*SRC*	Euteleostomi	12
693	*CCL22*	Euteleostomi	12	746	*IL6ST*	Euteleostomi	12	799	*STAT1*	Euteleostomi	12
694	*CCR6*	Euteleostomi	12	747	*IL7R*	Euteleostomi	12	800	*STAT3*	Euteleostomi	12
695	*CD14*	Euteleostomi	12	748	*IRGM*	Euteleostomi	12	801	*TAFA5*	Euteleostomi	12
696	*CD209*	Euteleostomi	12	749	*ITGA2*	Euteleostomi	12	802	*TFEB*	Euteleostomi	12
697	*CD34*	Euteleostomi	12	750	*ITGAX*	Euteleostomi	12	803	*TLR1*	Euteleostomi	12
698	*CD68*	Euteleostomi	12	751	*JCAD*	Euteleostomi	12	804	*TLR6*	Euteleostomi	12
699	*CDH1*	Euteleostomi	12	752	*KERA*	Euteleostomi	12	805	*TNFRSF12A*	Euteleostomi	12
700	*CDH13*	Euteleostomi	12	753	*KIT*	Euteleostomi	12	806	*TNNT2*	Euteleostomi	12
701	*CDH4*	Euteleostomi	12	754	*KITLG*	Euteleostomi	12	807	*TOX*	Euteleostomi	12
702	*CDH7*	Euteleostomi	12	755	*LUM*	Euteleostomi	12	808	*TPM2*	Euteleostomi	12
703	*CHGA*	Euteleostomi	12	756	*LYN*	Euteleostomi	12	809	*TPSAB1*	Euteleostomi	12
704	*CLEC4E*	Euteleostomi	12	757	*MARK4*	Euteleostomi	12	810	*TREML4*	Euteleostomi	12
705	*CLEC6A*	Euteleostomi	12	758	*MAST4*	Euteleostomi	12	811	*UCN*	Euteleostomi	12
706	*CLEC7A*	Euteleostomi	12	759	*MCF2L*	Euteleostomi	12	812	*UCN2*	Euteleostomi	12
707	*CLIC4*	Euteleostomi	12	760	*MFGE8*	Euteleostomi	12	813	*UTS2B*	Euteleostomi	12
708	*CMKLR1*	Euteleostomi	12	761	*MGP*	Euteleostomi	12	814	*APOL1*	Sarcopterygii	13
709	*CNTN1*	Euteleostomi	12	762	*MMP1*	Euteleostomi	12	815	*CCR1*	Sarcopterygii	13
710	*CSF1*	Euteleostomi	12	763	*MMP10*	Euteleostomi	12	816	*CYP2A6*	Sarcopterygii	13
711	*CXCL5*	Euteleostomi	12	764	*MMP12*	Euteleostomi	12	817	*IL15*	Sarcopterygii	13
712	*CXCR6*	Euteleostomi	12	765	*MMP3*	Euteleostomi	12	818	*KISS1*	Sarcopterygii	13
713	*DAB2IP*	Euteleostomi	12	766	*MMP7*	Euteleostomi	12	819	*MNDA*	Sarcopterygii	13
714	*DDIT3*	Euteleostomi	12	767	*MMP8*	Euteleostomi	12	820	*PDPN*	Sarcopterygii	13
715	*DLGAP1*	Euteleostomi	12	768	*MRTFA*	Euteleostomi	12	821	*SERPINA12*	Sarcopterygii	13
716	*EPHA2*	Euteleostomi	12	769	*MYOCD*	Euteleostomi	12	822	*SIRPA*	Sarcopterygii	13
717	*EREG*	Euteleostomi	12	770	*NCOA2*	Euteleostomi	12	823	*CD69*	Tetrapoda	15
718	*ESAM*	Euteleostomi	12	771	*NFATC2*	Euteleostomi	12	824	*ICOS*	Tetrapoda	15
719	*ESM1*	Euteleostomi	12	772	*NFATC3*	Euteleostomi	12	825	*OR6A2*	Tetrapoda	15
720	*F3*	Euteleostomi	12	773	*NOD2*	Euteleostomi	12	826	*SCRG1*	Tetrapoda	15
721	*FCAMR*	Euteleostomi	12	774	*NPPA*	Euteleostomi	12	827	*TRIM7*	Tetrapoda	15
722	*FMO3*	Euteleostomi	12	775	*NPPB*	Euteleostomi	12	828	*UTS2*	Tetrapoda	15
723	*FPR1*	Euteleostomi	12	776	*NRG1*	Euteleostomi	12	829	*APOC3*	Amniota	16
724	*FPR2*	Euteleostomi	12	777	*NRP1*	Euteleostomi	12	830	*CD160*	Amniota	16
725	*GJA1*	Euteleostomi	12	778	*NRP2*	Euteleostomi	12	831	*CYP2C19*	Amniota	16
726	*GPR65*	Euteleostomi	12	779	*NTM*	Euteleostomi	12	832	*CYP2C9*	Amniota	16
727	*GRM8*	Euteleostomi	12	780	*P2RY12*	Euteleostomi	12	833	*FCGR2A*	Amniota	16
728	*HAMP*	Euteleostomi	12	781	*PDE1A*	Euteleostomi	12	834	*FCGR3A*	Amniota	16
729	*HAVCR2*	Euteleostomi	12	782	*PDE4D*	Euteleostomi	12	835	*FCGR3B*	Amniota	16
730	*HBA1*	Euteleostomi	12	783	*PDGFRB*	Euteleostomi	12	836	*HFE*	Amniota	16
731	*HDAC4*	Euteleostomi	12	784	*PFN3*	Euteleostomi	12	837	*HLA-DRB1*	Amniota	16

Table S1 (continued).

Human gene		The most recent common ancestor		Human gene		The most recent common ancestor		Human gene		The most recent common ancestor	
#	*Symbol*	Taxon	PAI	#	*Symbol*	Taxon	PAI	#	*Symbol*	Taxon	PAI
838	*IL23A*	Amniota	16	845	*ZNF202*	Amniota	16	851	*IL5*	Theria	18
839	*IL9*	Amniota	16	846	*IL27*	Mammalia	17	852	*ZNF213*	Theria	18
840	*KLRK1*	Amniota	16	847	*RNASE3*	Mammalia	17	853	*IL33*	Eutheria	19
841	*PROCR*	Amniota	16	848	*C9orf50*	Theria	18	854	*MS4A13*	Eutheria	19
842	*SPP1*	Amniota	16	849	*CD70*	Theria	18	855	*OSM*	Eutheria	19
843	*TNFSF4*	Amniota	16	850	*GP6*	Theria	18	856	*TUG1*	ND	ND
844	*TREM1*	Amniota	16								
the arithmetic mean (MEAN) ± standard error of the mean (SEM): 5.41 ± 0.16											
The arithmetic mean of the PAI indices for the subset (a) of 16 hub genes for atherogenesis, atheroprotection and atherosclerosis is statistically significantly less than that for each of the five remaining subsets (b, c, d, e, and f) of the human genes associated with atherogenesis, atheroprotection or atherosclerosis.								the exact test of the binomial distribution, *p*-value (significance):		0.03 ($p < 0.05$)	

N*otes*. Hereinafter, ND: not detected; underlined, PAI, a gene's phylostratigraphic age index evaluated against the BLAST-based scale [106] using the freely available web service Orthoscape [107]; BLAST-based PAI scale: 0, Cellular organisms; 1, Eukaryota; 2, Opisthokonta; 3, Metazoa; 4, Eumetazoa; 5, Bilateria; 6, Deuterostomia; 7, Chordata; 8, Craniata; 9, Vertebrata; 10, Gnathostomata; 11, Teleostomi; 12, Euteleostomi; 13, Sarcopterygii; 14, Dipnotetrapodomorpha; 15, Tetrapoda; 16, Amniota; 17, Mammalia; 18, Theria; 19, Eutheria; 20, Euarchontoglires; 21, Primates; 22, Haplorrhini; 23, Simiiformes; 24, Catarrhini; 25, Hominoidea; 26, Hominidae; 27, Homininae; 28, Homo.; Genes: *AAK1*, AP2 associated kinase 1; *ABCA1*, ATP binding cassette subfamily A member 1; *ABCB1*, ATP binding cassette subfamily B member 1; *ABCC6*, ATP binding cassette subfamily C member 6; *ABCD1*, ATP binding cassette subfamily D member 1; *ABCG1*, *ABCG5*, and *ABCG8*, ATP binding cassette subfamily G members 1, 5 and 8, respectively; *ABI1* and *ABI2*, ABL interactors 1 and 2, respectively; *ABL1*, ABL proto-oncogene 1; *ABO*, α1-3-N-acetylgalactosaminyltransferase and α1-3-galactosyltransferase; *ACE*, angiotensin I converting enzyme; *ACE2*, angiotensin converting enzyme 2; *ACKR3*, atypical chemokine receptor 3; *ACP5*, acid phosphatase 5; *ACSL1*, acyl-CoA synthetase long chain family member 1; *ACTA2*, actin α2; *ADAM8*, *ADAM9*, *ADAM10*, *ADAM15*, *ADAM17*, and *ADAM33*, ADAM metallopeptidase domains 8, 9, 10, 15, 17, and 33, respectively; *ADAMTS1*, *ADAMTS3*, *ADAMTS4*, *ADAMTS5*, *ADAMTS7*, and *ADAMTS13*, ADAM metallopeptidase with thrombospondin type 1 motifs 1, 3, 4, 5, 7, and 13, respectively; *ADGRL2*, adhesion G protein-coupled receptor L2; *ADIPOQ*, adiponectin, C1Q and collagen domain containing; *ADIPOR1* and *ADIPOR2*, adiponectin receptor 1 and 2, respectively; *ADM*, adrenomedullin; *ADRB2* and *ADRB3*, adrenoceptors β2 and β3, respectively; *ADTRP*, androgen dependent TFPI regulating protein; *AFF3*, ALF transcription elongation factor 3; *AGER*, advanced glycosylation end-product specific receptor; *AGT*, angiotensinogen; *AGTR1* and *AGTR2*, angiotensin II receptors type 1 and 2, respectively; *AGXT2*, alanine--glyoxylate aminotransferase 2; *AHR*, aryl hydrocarbon receptor; *AHRR*, aryl hydrocarbon receptor repressor; *AHSG*, α2-HS glycoprotein; *AIF1*, allograft inflammatory factor 1; *AIRE*, autoimmune regulator; *AKR1B1* and *AKR1B10*, aldo-keto reductase family 1 members B and B10, respectively; *AKT1* and *AKT3*, AKT serine/threonine kinases 1 and 3, respectively; *AKT1S1*, AKT1 substrate 1; *ALB*, albumin; *ALDH2*, aldehyde dehydrogenase 2 family member; *ALDH4A1*, aldehyde dehydrogenase 4 family member A1; *ALMS1*, ALMS1 centrosome and basal body associated protein; *ALOX5*, *ALOX12*, and *ALOX15*, arachidonate 5-, 12- (12S type), and 15-lipoxygenases, respectively; *ALOX5AP*, arachidonate 5-lipoxygenase activating protein; *AMH*, anti-Mullerian hormone; *ANG*, angiogenin; *ANGPT1*, *ANGPT2*, *ANGPTL2*, *ANGPTL3*, *ANGPTL4*, and *ANGPTL8*, angiopoietins 1, 2, like 2; like 3, like 4, and like 8, respectively; *ANKRD6*, ankyrin repeat domain 6; *ANXA1* and *ANXA5*, annexin A1 and A5, respectively; *AOC3*, amine oxidase copper containing 3; *AP1B1*, adaptor related protein complex 1 subunit β1; *APCDD1*, APC down-regulated 1; *APCS*, amyloid P component; *APH1B*, aph-1 homolog B; *APLN*, apelin; *APLNR*, apelin receptor; *APOA1*, *APOA2*, *APOA4*, *APOA5*, *APOB*, *APOC1*, *APOC2*, *APOC3*, *APOE*, *APOH*, and *APOL1*, apolipoproteins A1, A2, A4, A5, B, C1, C2, C3, E, H, and L1, respectively; *APOBR*, apolipoprotein B receptor; *APP*, amyloid β precursor protein; *AQP1* and *AQP9*, aquaporin 1 (Colton blood group) and 9, respectively; *AR*, androgen receptor; *ARG1* and *ARG2*, arginases 1 and 2, respectively; *ARID5B*, AT-rich interaction domain 5B; *ARSB*, arylsulfatase B; *ASXL2*, ASXL transcriptional regulator 2; *ATF3*, activating transcription factor 3; *ATG16L1*, autophagy related 16 like 1; *ATG7*, autophagy related 7; *ATP6V1C2*, ATPase H+ transporting V1 subunit C2; *AURKA*, aurora kinase A; *AVP*, arginine vasopressin; *AXL*, AXL receptor tyrosine kinase; *B2M*, β2-microglobulin; *BAMBI*, BMP and activin membrane bound inhibitor; *BCL2*, BCL2 apoptosis regulator; *BCO1*, β-carotene oxygenase 1; *BDKRB1*, bradykinin receptor B1; *BDNF*, brain derived neurotrophic factor; *BGLAP*, bone γ-carboxyglutamate protein; *BGN*, biglycan; *BMP2* and *BMP4*, bone morphogenetic proteins 2 and 4, respectively; *BNC2*, basonuclin 2; *BPIFB4*, BPI fold containing family B member 4; *BRAP*, BRCA1 associated protein; *BRCA1*, BRCA1 DNA repair associated; *BRINP3*, BMP/retinoic acid inducible neural specific 3; *BSG*, basigin (Ok blood group); *C1QC*, complement C1q C chain; *C1QTNF1*, *C1QTNF3*, *C1QTNF9*, and *C1QTNF12*, C1q and TNF related proteins 1, 3, 9, and 12, respectively; *C2*, *C3*, *C4A*, and *C5*, complement C2, C3, C4A (Rodgers blood group), and C5, respectively; *C9orf50*, chromosome 9 open reading frame 50 (uncharacterized protein); *CA2* and *CA12*, carbonic anhydrases 2 and 12, respectively; *CACNA1C* and *CACNA2D1*, calcium voltage-gated channel subunits α1C and α2δ1, respectively; *CADPS*, calcium dependent secretion activator; *CALCA*, calcitonin related polypeptide α; *CAMK2D*, calcium/calmodulin dependent protein kinase IIδ; *CAMKK2*, calcium/calmodulin dependent protein kinase kinase 2; *CAMP*, cathelicidin antimicrobial peptide; *CANX*, calnexin; *CAP1*, cyclase associated actin cytoskeleton regulatory protein 1; *CAPG*, capping actin protein, gelsolin like; *CAPN10*, calpain 10; *CARD8*, caspase recruitment domain family member 8; *CARHSP1*, calcium regulated heat stable protein 1; *CASP3*, caspase 3; *CASR*, calcium sensing receptor; *CAT*, catalase; *CAV1*, caveolin 1; *CCDC178*, coiled-coil domain containing 178; *CCL2*, *CCL5*, *CCL7*, *CCL11*, *CCL17*, *CCL19*, *CCL22*, *CCL23*, and *CCL28*, C-C motif chemokine ligands 2, 5, 7, 11, 17, 19, 22, 23, and 28, respectively; *CCN2*, cellular communication network factor 2; *CCNB2*, cyclin B2; *CCR1*, *CCR2*, *CCR5*, *CCR6*, C-C motif chemokine receptors 1, 2, 5, and 6, respectively; *CD4*, *CD5L*, *CD9*, *CD14*, *CD28*, *CD34*, *CD36*, *CD40*, *CD44*, *CD47*, *CD59*, *CD68*, *CD69*, *CD70*, *CD86*, *CD160*, *CD163*, *CD209*, and *CD248*, molecules CD4, CD5 like, CD9,CD14, CD28, CD34, CD36, CD40, CD44 (Indian blood group), CD47, CD59 (CD59 blood group), CD68, CD69, CD70, CD86, CD160, CD163, CD209, and CD248, respectively; *CD40LG*, CD40 ligand; *CDC20B* and *CDC42*, cell division cycle proteins 20B and 42, respectively; *CDH1*, *CDH4*, *CDH5*, *CDH7*, and *CDH13*, cadherins 1, 4, 5, 7,

and13, respectively; *CDK5* and *CDK9*, cyclin dependent kinases 5 and 9, respectively; *CDK5RAP3*, CDK5 regulatory subunit associated protein 3; *CDKN1A*, *CDKN1B*, *CDKN1C*, *CDKN2A*, *CDKN2B*, and *CDKN3*, cyclin dependent kinase inhibitors 1A, 1B, 1C, 2A, 2B, and 3, respectively; *CEL*, carboxyl ester lipase; *CELA2A*, chymotrypsin like elastase 2A; *CELSR1* and *CELSR2*, cadherin EGF LAG seven-pass G-type receptors 1 and 2, respectively; *CEMIP*, cell migration inducing hyaluronidase 1; *CERS5*, ceramide synthase 5; *CETP*, cholesteryl ester transfer protein; *CFAP91*, cilia and flagella associated protein 91; *CFH*, complement factor H; *CHGA*, chromogranin A; *CHI3L1*, chitinase 3 like 1; *CHIT1*, chitinase 1; *CILP2*, cartilage intermediate layer protein 2; *CLEC4E*, C-type lectin domain family 4 member E; *CLEC6A* and *CLEC7A*, C-type lectin domain containing proteins 6Aand 7A, respectively; *CLIC4*, chloride intracellular channel 4; *CLTCL1*, clathrin heavy chain like 1; *CMKLR1*, chemerin chemokine-like receptor 1; *CMPK2*, cytidine/uridine monophosphate kinase 2; *CNOT3*, CCR4-NOT transcription complex subunit 3; *CNR1*, cannabinoid receptor 1; *CNTN1*, contactin 1; *COL4A2*, *COL5A1*, *COL12A1*, and *COL18A1*, collagen chains type IVα2, Vα1, XIIα1, and XVIIIα1, respectively; *COMMD10*, COMM domain containing 10; *COMP*, cartilage oligomeric matrix protein; *COMT*, catechol-O-methyltransferase; *CORIN*, corin, serine peptidase; *CP*, ceruloplasmin; *CPB2* and *CPE*, carboxypeptidases B2 and E, respectively; *CPS1*, carbamoyl-phosphate synthase 1; *CPT1A*, carnitine palmitoyltransferase 1A; *CREB1*, *CREB3*, and *CREB3L3*, cAMP responsive element binding proteins 1, 3, and 3 like 3, respectively; *CRISP2*, cysteine rich secretory protein 2; *CRP*, C-reactive protein; *CRY1*, cryptochrome circadian regulator 1; *CSF1*, colony stimulating factor 1; *CSGALNACT2*, chondroitin sulfate N-acetylgalactosaminyltransferase 2; *CSRP1*, cysteine and glycine rich protein 1; *CST3*, cystatin C; *CTH*, cystathionine γ-lyase; *CTNNA3* and *CTNNB1*, catenins α3 and β1, respectively; *CTSB*, *CTSC*, *CTSD*, *CTSG*, *CTSK*, *CTSL*, and *CTSS*, cathepsins B, C, D, G, K, L, and S, respectively; *CTTN*, cortactin; *CX3CL1*, C-X3-C motif chemokine ligand 1; *CX3CR1*, C-X3-C motif chemokine receptor 1; *CXCL1*, *CXCL5*, *CXCL8*, *CXCL12*, *CXCL13*, and *CXCL16*, C-X-C motif chemokine ligands 1, 5, 8, 12, 13, and 16, respectively; *CXCR2*, *CXCR3*, *CXCR4*, *CXCR5*, and *CXCR6*, C-X-C motif chemokine receptors 2, 3, 4, 5, and 6, respectively; *CYBA* and *CYBB*, cytochrome b-245 chains α and β, respectively; *CYLD*, CYLD lysine 63 deubiquitinase; *CYP11B2*, cytochrome P450 family 11 subfamily B member 2; *CYP19A1*, cytochrome P450 family 19 subfamily A member 1; *CYP26B1*, cytochrome P450 family 26 subfamily B member 1; *CYP27A1*, cytochrome P450 family 27 subfamily A member 1; *CYP27C1*, cytochrome P450 family 27 subfamily C member 1; *CYP2A6*, cytochrome P450 family 2 subfamily A member 6; *CYP2C9 and CYP2C19*, cytochrome P450 family 2 subfamily C members 9 and 19, respectively; *CYP4V2*, cytochrome P450 family 4 subfamily V member 2; *CYP7B1*, cytochrome P450 family 7 subfamily B member 1; *CYSLTR1*, cysteinyl leukotriene receptor 1; *DAB2IP*, DAB2 interacting protein; *DAP*, death associated protein; *DCN*, decorin; *DDAH1* and *DDAH2*, dimethylarginine dimethylaminohydrolases 1 and 2, respectively; *DDIT3*, DNA damage inducible transcript 3; *DGKH*, diacylglycerol kinase η; *DHCR7*, 7-dehydrocholesterol reductase; *DHX15* and *DHX38*, DEAH-box helicases 15 and 38, respectively; *DICER1*, dicer 1, ribonuclease III; *DKK1* and *DKK3*, dickkopf WNT signaling pathway inhibitors 1 and 3, respectively; *DLGAP1*, DLG associated protein 1; *DLL4*, δ like canonical Notch ligand 4; *DNAH5*, dynein axonemal heavy chain 5; *DNMT1* and *DNMT3A*, DNA methyltransferases 1 and 3α, respectively; *DOCK4* and *DOCK7*, dedicators of cytokinesis 4 and 7, respectively; *DOT1L*, DOT1 like histone lysine methyltransferase; *DPP4*, dipeptidyl peptidase 4; *EBI3*, Epstein-Barr virus induced 3; *ECE1*, endothelin converting enzyme 1; *EDN1* and *EDN3*, endothelins 1 and 3, respectively; *EDNRA* and *EDNRB*,endothelin receptors type A and B, respectively; *EEF2K*, eukaryotic elongation factor 2 kinase; *EFEMP1*, EGF containing fibulin extracellular matrix protein 1; *EGF*, epidermal growth factor; *ELANE*, elastase, neutrophil expressed; *ELAVL1*, ELAV like RNA binding protein 1; *ELN*, elastin; *ELOVL2*, ELOVL fatty acid elongase 2; *ENG*, endoglin; *ENO1*, enolase 1; *ENTPD1*, ectonucleoside triphosphate diphosphohydrolase 1; *EPHA2*, EPH receptor A2; *EPHX2*, epoxide hydrolase 2; *ERCC1*, ERCC excision repair 1, endonuclease non-catalytic subunit; *EREG*, epiregulin; *ERN1*, endoplasmic reticulum to nucleus signaling 1; *ESAM*, endothelial cell adhesion molecule; *ESM1*, endothelial cell specific molecule 1; *ESR1* and *ESR2*, estrogen receptors 1 and 2, respectively; *ETS1* and *ETS2*, ETS proto-oncogenes 1 and 2, respectively; *EVA1A*, eva-1 homolog A, regulator of programmed cell death; *EYS*, eyes shut homolog; *EZH2*, enhancer of zeste 2 polycomb repressive complex 2 subunit; *F11R*, F11 receptor; *F2*, *F3*, *F5*, *F7*, *F8*,*F12*, coagulation factors II (thrombin), III, V, VII, VIII, XII, respectively; *F2R*, coagulation factor II thrombin receptor; *F2RL1*, F2R like trypsin receptor 1; *F2RL2*, coagulation factor II thrombin receptor like 2; *FABP2*, *FABP3*, *FABP4*, and *FABP5*, fatty acid binding proteins 2, 3, 4 and 5, respectively; *FADS1*, *FADS2*, and *FADS3*, fatty acid desaturases 1, 2, and 3, respectively; *FASLG*, Fas ligand; *FBLIM1*, filamin binding LIM protein 1; *FBXO3*, F-box protein 3; *FCAMR*, Fc α and μ receptor; *FCGR2A*, *FCGR3A*, and *FCGR3B*, Fc γ receptors IIa, IIIa, and IIIb, respectively; *FETUB*, fetuin B; *FGF19*, *FGF21*, and *FGF23*, fibroblast growth factors 19, 21, and 23, respectively; *FLT1*, fms related receptor tyrosine kinase 1; *FMO3*, flavin containing dimethylaniline monoxygenase 3; *FN1*, fibronectin 1; *FNDC5*, fibronectin type III domain containing 5; *FOLR2*, folate receptor β; *FOXA3*, *FOXC2*, *FOXO1*, *FOXO3*, *FOXO4*, and *FOXP3*, forkhead boxes A3, C2, O1, O3, O4, and P3, respectively; *FPR1*, and *FPR2*, formyl peptide receptors 1 and 2, respectively; *FRS2*, fibroblast growth factor receptor substrate 2; *FSTL1*, follistatin like 1; *FURIN*, furin; *G6PC2*, glucose-6-phosphatase catalytic subunit 2; *GALNT2*, polypeptide N-acetylgalactosaminyltransferase 2; *GAS6*, growth arrest specific 6;*GATA2* and *GATA6*, GATA binding proteins 2 and 6, respectively; *GC*, GC vitamin D binding protein; *GCG*, glucagon; *GCKR*, glucokinase regulator; *GCLC*, glutamate-cysteine ligase catalytic subunit; *GDF2*, *GDF11*, and *GDF15*, growth differentiation factors 2, 11, and 15, respectively; *GGT1*, γ-glutamyltransferase 1; *GHRL*, ghrelin and obestatin prepropeptide; *GHSR*, growth hormone secretagogue receptor; *GJA1* nad *GJA4*, gap junction proteins α1 and α4, respectively; *GLO1*, glyoxalase I; *GLS2*, glutaminase 2; *GLTPD2*, glycolipid transfer protein domain containing 2; *GNAI2* and *GNB3*, G protein subunits αi2 and β3, respectively; *GNMT*, glycine N-methyltransferase; *GP6*, glycoprotein VI platelet; *GPBP1*, GC-rich promoter binding protein 1; *GPER1*, G protein-coupled estrogen receptor 1; *GPR18*, *GPR26*, *GPR55*, *GPR65*, *GPR146*, G protein-coupled receptors 18, 26, 55, 65, and 146, respectively; *GPS2*, G protein pathway suppressor 2; *GPT*, glutamic--pyruvic transaminase; *GPX3*, glutathione peroxidase 3; *GREM1*, gremlin 1, DAN family BMP antagonist; *GRK6*, G protein-coupled receptor kinase 6; *GRM8*, glutamate metabotropic receptor 8; *GRN*, granulin precursor; *GSC2*, goosecoid homeobox 2; *GSK3B*, glycogen synthase kinase 3β; *GSTA4*, *GSTM1*, *GSTO1*, and *GSTP1*, glutathione S-transferases α4, μ1, ω1, and π1, respectively; *GTF2E2*, general transcription factor IIE subunit 2; *H6PD*, hexose-6-phosphate dehydrogenase/glucose 1-dehydrogenase; *HABP2*, hyaluronan binding protein 2; *HACD4*, 3-hydroxyacyl-CoA dehydratase 4; *HAMP*, hepcidin antimicrobial peptide; *HAS2*, hyaluronan synthase 2; *HAVCR2*, hepatitis A virus cellular receptor 2; *HBA1*, hemoglobin subunit α1; *HBEGF*, heparin binding EGF like growth factor; *HCAR1* nad *HCAR2*, hydroxycarboxylic acid receptors 1 and 2, respectively; *HDAC1*, *HDAC2*, *HDAC4*, *HDAC5*, and *HDAC9*, histone deacetylases 1, 2, 4, 5, and 9, respectively; *HERC6*, HECT and RLD domain containing E3 ubiquitin protein ligase family member 6; *HERPUD1*, homocysteine inducible ER protein with ubiquitin like domain 1; *HFE*, homeostatic iron regulator; *HGF*, hepatocyte growth factor; *HGFAC*, HGF activator; *HIF1A*, hypoxia inducible factor 1 subunit α; *HLA-DRB1*, major histocompatibility complex, class II, DR β1; *HMGB1*, high mobility group box 1; *HMGCR*, 3-hydroxy-3-methylglutaryl-CoA reductase; *HMOX1*, heme oxygenase 1; *HNF1A*, HNF1 homeobox A; *HNRNPC*, heterogeneous nuclear ribonucleoprotein C; *HNRNPD*, heterogeneous nuclear ribonucleoprotein D; *HOMER1* and *HOMER2*, homer scaffold proteins 1 and 2, respectively; *HOXA1*, *HOXA5*, and *HOXC6*, homeoboxs A1, A5, and C6, respectively; *HP*, haptoglobin; *HPR*, haptoglobin-related protein; *HPSE*, heparanase; *HSPA1A*, *HSPA4*, *HSPA5*, *HSPA8*, and *HSPA12B*, heat shock protein family A (Hsp70) members 1A, 4, 5, 8, and 12B, respectively; *HSPB1*, heat shock protein family B (small) member 1; *HSPD1*, heat shock protein family D (Hsp60) member 1; *HSPG2*, heparan sulfate proteoglycan 2; *HYAL1*, hyaluronidase 1; *ICA1*, islet cell autoantigen 1; *ICAM1*, intercellular adhesion molecule 1; *ICOS*, inducible T cell costimulator; *ID3*, inhibitor of DNA binding 3; *IDO1*, indoleamine 2,3-dioxygenase 1; *IFNB1* and *IFNG*, interferons β1 and γ, respectively; *IGF1* and *IGF2*, insulin like growth factors 1 and 2, respectively; *IGF1R*, insulin like growth factor 1 receptor; *IGFALS*, insulin like growth factor binding protein acid labile subunit; *IGFBP1*, *IGFBP3* and *IGFBP5*, insulin like growth factor binding proteins 1, 3, and 5, respectively; *IKBKB* and *IKBKE*, inhibitor of nuclear factor κB kinase subunits β and ε, respectively;

IL1A, *IL1B*, *IL4*, *IL5*, *IL6*, *IL7*, *IL9*, *IL10*, *IL12A*, *IL15*, *IL17A*, *IL18*, *IL20*, *IL21*, *IL22*, *IL23A*, *IL27*, *IL32*, *IL33*, and *IL37*, interleukins 1α, 1β, 4, 5, 6, 7, 9, 10, 12A, 15, 17A, 18, 20, 21, 22, 23 subunit α, 27, 32, 33, and 37, respectively; *IL1RN*, interleukin 1 receptor antagonist; *IL2RA*, interleukin 2 receptor subunit α; *IL6R*, interleukin 6 receptor; *IL6ST*, interleukin 6 cytokine family signal transducer; *IL7R*, interleukin 7 receptor; *IL23R*, interleukin 23 receptor; *ILK*, integrin linked kinase; *INS*, insulin; *IRF1* and *IRF7*, interferon regulatory factors 1 and 7, respectively; *IRGM*, immunity related GTPase M; *IRS1* and *IRS2*, insulin receptor substrates 1 and 2, respectively; *ISM1*, isthmin 1; *ITGA2*, *ITGA2B*, *ITGA4*, *ITGA5*, *ITGAM*, *ITGAV*, *ITGAX*, *ITGB1*, *ITGB2*, *ITGB3*, *ITGB4*, and *ITGB5*, integrin subunits α2, α2b, α4, α5, αM, αV, αX, β1, β2, β3, β4, and β5, respectively; *ITLN1*, intelectin 1; *JAG1*, jagged canonical Notch ligand 1; *JAK1* and *JAK2*, Janus kinases 1 and 2, respectively; *JAM3*, junctional adhesion molecule 3; *JCAD*, junctional cadherin 5 associated; *JUN*, Jun proto-oncogene; *KCNJ5*, potassium inwardly rectifying channel subfamily J member 5; *KCNMB1*, potassium calcium-activated channel subfamily M regulatory β subunit 1; *KCNN4*, potassium calcium-activated channel subfamily N member 4; *KDR*, kinase insert domain receptor; *KERA*, keratocan; *KIF13B*, kinesin family member 13B; *KISS1*, KiSS-1 metastasis suppressor; *KISS1R*, KISS1 receptor; *KIT*, KIT proto-oncogene; *KITLG*, KIT ligand; *KL*, klotho; *KLF2*, *KLF3*, *KLF4*, and *KLF5*, KLF transcription factors 2, 3, 4, and 5, respectively; *KLKB1*, kallikrein B1; *KLRK1*, killer cell lectin like receptor K1; *LAMC1*, laminin subunit γ1; *LCAT*, lecithin-cholesterol acyltransferase; *LCN2*, lipocalin 2; *LDB2*, LIM domain binding 2; *LDLR*, low density lipoprotein receptor; *LECT2*, leukocyte cell derived chemotaxin 2; *LEP*, leptin; *LEPR*, leptin receptor; *LGALS1*, *LGALS2*, *LGALS3*, *LGALS12*, galectins 1, 2, 3, and 12, respectively; *LGALS3BP*, galectin 3 binding protein; *LGMN*, legumain; *LIAS*, lipoic acid synthetase; *LIPC* and *LIPG*, lipases C and G, respectively; *LMCD1*, LIM and cysteine rich domains 1; *LMNA*, lamin A/C; *LPA*, lipoprotein(a); *LPL*, lipoprotein lipase; *LPP*, LIM domain containing preferred translocation partner in lipoma; *LRP1*, *LRP2*, and *LRP6*, LDL receptor related proteins 1, 2, and 6, respectively; *LRRC18*, leucine rich repeat containing 18; *LRRIQ3*, leucine rich repeats and IQ motif containing 3; *LTA*, lymphotoxin α; *LTA4H*, leukotriene A4 hydrolase; *LTB4R*, leukotriene B4 receptor; *LTBR*, lymphotoxin β receptor; *LTC4S*, leukotriene C4 synthase; *LUM*, lumican; *LYN*, LYN proto-oncogene; *MAFF*, MAF bZIP transcription factor F; *MAN2B1*, mannosidase α class 2B member 1; *MAPK1*, *MAPK3*, *MAPK7*, and *MAPK14*, mitogen-activated protein kinases 1, 3, 7, and 14, respectively; *MARCO*, macrophage receptor with collagenous structure; *MARK4*, microtubule affinity regulating kinase 4; *MAST4*, microtubule associated serine/threonine kinase family member 4; *MATR3*, matrin 3; *MAZ*, MYC associated zinc finger protein; *MBL2*, mannose binding lectin 2; *MCAM*, melanoma cell adhesion molecule; *MCF2L*, MCF.2 cell line derived transforming sequence like; *MDK*, midkine; *MEF2C*, myocyte enhancer factor 2C; *MERTK*, MER proto-oncogene; *METRNL*, glial cell differentiation regulator meteorin like; *METTL3* and *METTL14*, methyltransferases 3 and 14, respectively; *MFGE8*, milk fat globule EGF and factor V/VIII domain containing; *MGAT5B*, α-1,6-mannosylglycoprotein 6-β-N-acetylglucosaminyltransferase B; *MGMT*, O-6-methylguanine-DNA methyltransferase; *MGP*, matrix Gla protein; *MIA3*, MIA SH3 domain ER export factor 3; *MIF*, macrophage migration inhibitory factor; *MLKL*, mixed lineage kinase domain like pseudokinase; *MMP1*, *MMP2*, *MMP3*, *MMP7*, *MMP8*, *MMP9*, *MMP10*, *MMP12*, and *MMP14*, matrix metallopeptidases 1, 2, 3, 7, 8, 9, 10, 12, and 14, respectively; *MNDA*, myeloid cell nuclear differentiation antigen; *MPO*, myeloperoxidase; *MRAS*, muscle RAS oncogene homolog; *MRTFA*, myocardin related transcription factor A; *MS4A13*, membrane spanning 4-domains A13; *MSR1*, macrophage scavenger receptor 1; *MSTN*, myostatin; *MTHFD2*, NADP+ dependent methylenetetrahydrofolate dehydrogenase 2, methenyltetrahydrofolate cyclohydrolase; *MTHFR*, methylenetetrahydrofolate reductase; *MTNR1B*, melatonin receptor 1B; *MTOR*, mechanistic target of rapamycin kinase; *MTTP*, microsomal triglyceride transfer protein; *MTUS1*, microtubule associated scaffold protein 1; *MYH9*, myosin heavy chain 9; *MYLIP*, myosin regulatory light chain interacting protein; *MYLK*, myosin light chain kinase; *MYO1E*, *MYO5A*, and *MYO7B*, myosins IE, VA, and VIIB, respectively; *MYOCD*, myocardin; *NALF1*, NALCN channel auxiliary factor 1; *NAMPT*, nicotinamide phosphoribosyltransferase; *NAT2* and *NAT8*, N-acetyltransferases 2 and 8 (putative), respectively; *NAXE*, NAD(P)HX epimerase; *NCEH1*, neutral cholesterol ester hydrolase 1; *NCF1*, neutrophil cytosolic factor 1; *NCOA2*, nuclear receptor coactivator 2; *NEIL3*, nei like DNA glycosylase 3; *NEU1*, neuraminidase 1; *NEXN*, nexilin F-actin binding protein; *NF2*, NF2, moesin-ezrin-radixin like (MERLIN) tumor suppressor; *NFATC2*, *NFATC3*, and *NFAT5*, nuclear factors of activated T cells 2, 3,and 5, respectively; *NFE2L2*, NFE2 like bZIP transcription factor 2; *NFKB1*, nuclear factor κB subunit 1; *NGB*, neuroglobin; *NGF*, nerve growth factor; *NINJ2*, ninjurin 2; *NKAPL*, NFKB activating protein like; *NLRP3*, NLR family pyrin domain containing 3; *NOD1* and *NOD2*, nucleotide binding oligomerization domain containing proteins 1and 2, respectively; *NOS1*, *NOS2*, and *NOS3*, nitric oxide synthases 1, 2, and 3, respectively; *NOS1AP*, nitric oxide synthase 1 adaptor protein; *NOTCH1*, *NOTCH2*, *NOTCH3*, and *NOTCH4*, notch receptors 1, 2, 3, and 4, respectively; *NOX1*, *NOX4*, and *NOX5*, NADPH oxidases 1, 4, and 5, respectively; *NPC1*, NPC intracellular cholesterol transporter 1; *NPC1L1*, NPC1 like intracellular cholesterol transporter 1; *NPM1*, nucleophosmin 1; *NPPA* and *NPPB*,natriuretic peptides A and B, respectively; *NPY*, neuropeptide Y; *NR1D1*, nuclear receptor subfamily 1 group D member 1; *NR1H3* and *NR1H4*, nuclear receptor subfamily 1 group H members 3 and 4, respectively; *NR1I2* and *NR1I3*, nuclear receptor subfamily 1 group I members 2 and 3, respectively; *NR3C1* and *NR3C2*, nuclear receptor subfamily 3 group C members 1 and 2, respectively; *NR4A1*, *NR4A2*, and *NR4A3*, nuclear receptor subfamily 4 group A members 1, 2, and 3, respectively; *NRG1* and *NRG4*, neuregulins 1 and 4, respectively; *NRP1* and *NRP2*, neuropilins 1 and 2, respectively; *NTM*, neurotrimin; *NTN1* and *NTNG1*, netrins 1 and G1, respectively; *NTRK2*, neurotrophic receptor tyrosine kinase 2; *NUGGC*, nuclear GTPase, germinal center associated; *NUMB*, NUMB endocytic adaptor protein; *NUP98*, nucleoporin 98 and 96 precursor; *OGN*, osteoglycin; *OLR1*, oxidized low density lipoprotein receptor 1; *OR6A2*, olfactory receptor family 6 subfamily A member 2; *ORMDL3*, ORMDL sphingolipid biosynthesis regulator 3; *OSBP2* and *OSBPL1A*, oxysterol binding proteins 2and like 1A, respectively; *OSBPL8*, oxysterol binding protein like 8; *OSCAR*, osteoclast associated Ig-like receptor; *OSM*, oncostatin M; *OSMR*, oncostatin M receptor; *P2RX7*, *P2RY2*, and *P2RY12*, purinergic receptors P2X7, P2Y2, and P2Y12, respectively; *PAFAH1B2* and *PAFAH1B3*, platelet activating factor acetylhydrolase 1b catalytic subunits 2 and 3, respectively; *PAK1*, p21 (RAC1) activated kinase 1; *PALLD*, palladin; *PAPPA*, pappalysin 1; *PARK7*, Parkinsonism associated deglycase; *PARP1*, poly(ADP-ribose) polymerase 1; *PCMT1*, protein-L-isoaspartate (D-aspartate) O-methyltransferase; *PCOLCE2*, procollagen C-endopeptidase enhancer 2; *PCSK6* and *PCSK9*, proprotein convertases subtilisin/kexin type 6 and 9, respectively; *PCYOX1*, prenylcysteine oxidase 1; *PDCD4*, programmed cell death 4; *PDE1A* and *PDE4D*, phosphodiesterases 1A and 4D, respectively; *PDGFC* and *PDGFD*, platelet derived growth factors C and D, respectively; *PDGFRB*, platelet derived growth factor receptor β; *PDPN*, podoplanin; *PDSS2*, decaprenyl diphosphate synthase subunit 2; *PECAM1*, platelet and endothelial cell adhesion molecule 1; *PEPD*, peptidase D; *PF4*, platelet factor 4; *PFN1* and *PFN3*, profilins 1 and 3, respectively; *PGF*, placental growth factor; *PGLYRP1*, peptidoglycan recognition protein 1; *PHACTR1*, phosphatase and actin regulator 1; *PIEZO1*, piezo type mechanosensitive ion channel component 1; *PIGR*, polymeric immunoglobulin receptor; *PIK3CB* and *PIK3CG*, phosphatidylinositol-4,5-bisphosphate 3-kinase catalytic subunits β and γ; *PIWIL1*, piwi like RNA-mediated gene silencing 1; *PLA2G2A*, *PLA2G3*, *PLA2G6*, *PLA2G7*, and *PLA2G10*, phospholipases A2 group IIA, III, VI, VII, and X, respectively; *PLAT*, *PLAU*, and *PLAUR*, plasminogen activators, tissue type, urokinase, and urokinase receptor, respectively; *PLCG1*, phospholipase C γ1; *PLEKHO1*, pleckstrin homology domain containing O1; *PLG*, plasminogen; *PLIN2*, perilipin 2; *PLK1*, polo like kinase 1; *PLPP3*, phospholipid phosphatase 3; *PLTP*, phospholipid transfer protein; *PNPLA3*, patatin like phospholipase domain containing 3; *PON1*, *PON2*,and *PON3*, paraoxonases 1, 2, and 3, respectively; *PPARA*, *PPARD*, and *PPARG*, peroxisome proliferator activated receptors α, δ, and γ, respectively; *PPARGC1A*, PPARG coactivator 1α; *PPBP*, pro-platelet basic protein; *PPIA*, peptidylprolyl isomerase A; *PPM1K*, Mg2+/Mn2+ dependent protein phosphatase 1K; *PPP1R3B* and *PPP1R12A*, protein phosphatase 1 regulatory subunits 3B and 12A, respectively; *PRDX1*, peroxiredoxin 1; *PRKAA1* and *PRKAA2*, protein kinase AMP-activated catalytic subunit α1 and α2; *PRKCB*, *PRKCE*, and *PRKCZ*, protein kinases Cβ, Cε, and Cζ, respectively; *PRLR*, prolactin receptor; *PRMT2*, protein arginine methyltransferase 2; *PROC*, protein C; *PROCR*, protein C receptor; *PROM1*, prominin 1; *PROS1* and *PROZ*, proteins S and Z, respectively; *PRTFDC1*, phosphoribosyl transferase domain

containing 1; *PSMA6*, proteasome 20S subunit α6; *PSMD6*, proteasome 26S subunit, non-ATPase 6; *PSME3*, proteasome activator subunit 3; *PTEN*, phosphatase and tensin homolog; *PTGDS*, prostaglandin D2 synthase; *PTGER4*, prostaglandin E receptor 4; *PTGES*, prostaglandin E synthase; *PTGES3*, prostaglandin E synthase 3; *PTGIS*, prostaglandin I2 synthase; *PTGS2*, prostaglandin-endoperoxide synthase 2; *PTH*, parathyroid hormone; *PTPN2*, *PTPN6*, and *PTPN22*, protein tyrosine phosphatase non-receptors type 2, 6, and 22, respectively; *PTPRC*, protein tyrosine phosphatase receptor type C; *PTX3*, pentraxin 3; *PXDN*, peroxidasin; *QSOX1*, quiescin sulfhydryl oxidase 1; *RAB7A*, RAB7A, member RAS oncogene family; *RAP1A* and *RAP1B*, RAS oncogene family members RAP1A and RAP1B, respectively; *RARB*, retinoic acid receptor β; *RARRES2*, retinoic acid receptor responder 2; *RBP4*, retinol binding protein 4; *RCN2*, reticulocalbin 2; *RECK*, reversion inducing cysteine rich protein with kazal motifs; *RELA*, RELA proto-oncogene, NF-kB subunit; *RELN*, reelin; *RETN*, resistin; *RETNLB*, resistin like β; *RFPL1*, ret finger protein like 1; *RGCC*, regulator of cell cycle; *RGS5*, regulator of G protein signaling 5; *RHBDF2*, rhomboid 5 homolog 2; *RHO*, rhodopsin; *RHOA*, ras homolog family member A; *RIGI*, RNA sensor RIG-I; *RIPK1* and *RIPK2*, receptor interacting serine/threonine kinases 1 and 2, respectively; *RNASE3*, ribonuclease A family member 3; *RNF111* and *RNF213*, ring finger proteins 111 and 213, respectively; *ROBO4*, roundabout guidance receptor 4; *ROCK1* and *ROCK2*, Rho associated coiled-coil containing protein kinases 1 and 2, respectively; *RORA*, RAR related orphan receptor A; *RREB1*, ras responsive element binding protein 1; *RSAD2*, radical S-adenosyl methionine domain containing 2; *RSPO2*, R-spondin 2; *RTN3* and *RTN4*, reticulons 3 and 4, respectively; *RUNX3*, RUNX family transcription factor 3; *RYR3*, ryanodine receptor 3; *S100A8*, *S100A9*, and *S100A12*, S100 calcium binding proteins A8, A9, and A12, respectively; *SAA1* and *SAA2*, serum amyloids A1 and A2, respectively; *SAMD1* and *SAMD9*, sterile α motif domain containing proteins 1 and 9, respectively; *SCARB1* and *SCARB2*, scavenger receptor class B members 1 and 2, respectively; *SCARF1*, scavenger receptor class F member 1; *SCD*, stearoyl-CoA desaturase; *SCRG1*, stimulator of chondrogenesis 1; *SDC1*, syndecan 1; *SDHB*, succinate dehydrogenase complex iron sulfur subunit B; *SELE*, selectin E; *SELENOP* and *SELENOS*, selenoproteins P and S, respectively; *SELL* and *SELP*, selectins L and P, respectively; *SELPLG*, selectin P ligand; *SEMA3E* and *SEMA7A*, semaphorins 3E and 7A (John Milton Hagen blood group); *SERPINA1* and *SERPINA12*, serpin family A members 1 and 12, respectively; *SERPIND1*, serpin family D member 1; *SERPINE1*, serpin family E member 1; *SERPINF1*, serpin family F member 1; *SESN2*, sestrin 2; *SFTPD*, surfactant protein D; *SGK1*, serum/glucocorticoid regulated kinase 1; *SGMS2*, sphingomyelin synthase 2; *SH2B3*, SH2B adaptor protein 3; *SHB*, SH2 domain containing adaptor protein B; *SHBG*, sex hormone binding globulin; *SIRPA*, signal regulatory protein α; *SIRT1*, *SIRT3*, and *SIRT6*, sirtuins 1, 3, and 6, respectively; *SLAMF7*, SLAM family member 7; *SLC12A1*, solute carrier family 12 member 1; *SLC1A4*, solute carrier family 1 member 4; *SLC25A1*, solute carrier family 25 member 1; *SLC44A3*, solute carrier family 44 member 3; *SLC5A7* and *SLC5A10*, solute carrier family 5 members 7 and 10, respectively; *SLC6A4*, solute carrier family 6 member 4; *SLC7A5*, solute carrier family 7 member 5; *SLC9A1*, solute carrier family 9 member A1; *SLCO5A1*, solute carrier organic anion transporter family member 5A1; *SMAD1*, *SMAD5*, and *SMAD7*, SMAD family members 1, 5, and 7, respectively; *SMPD3*, sphingomyelin phosphodiesterase 3; *SNAI1*, snail family transcriptional repressor 1; *SNX10*, sorting nexin 10; *SOAT1*, sterol O-acyltransferase 1; *SOCS1* and *SOCS3*, suppressors of cytokine signaling 1 and 3, respectively; *SOD1* and *SOD2*, superoxide dismutases 1 and 2, respectively; *SORBS3*, sorbin and SH3 domain containing 3; *SORCS1*, sortilin related VPS10 domain containing receptor 1; *SORL1*, sortilin related receptor 1; *SORT1*, sortilin 1; *SOST*, sclerostin; *SOX4*, *SOX6*, *SOX18*, SRY-box transcription factors 4, 6, and 18, respectively; *SP1*, Sp1 transcription factor; *SPP1*, secreted phosphoprotein 1; *SPPL2A* and *SPPL2B*, signal peptide peptidases like 2A and 2B, respectively; *SPRY1*, sprouty RTK signaling antagonist 1; *SPRYD4*, SPRY domain containing 4; *SQSTM1*, sequestosome 1; *SRC*, SRC proto-oncogene; *SREBF1* and *SREBF2*, sterol regulatory element binding transcription factors 1 and 2, respectively; *SRY*, sex determining region Y; *ST6GAL1*, ST6 β-galactoside α-2,6-sialyltransferase 1; *ST8SIA1*, ST8 α-N-acetyl-neuraminide α-2,8-sialyltransferase 1; *STARD5* and *STARD7*, StAR related lipid transfer domain containing proteins 5 and 7 *ST6GAL1*; *STAT1* and *STAT3*, signal transducer and activator of transcription proteins 1 and 3, respectively; *STC2*, stanniocalcin 2; *STK11*, serine/threonine kinase 11; *STRN3*, striatin 3; *SUB1*, SUB1 regulator of transcription; *SUCNR1*, succinate receptor 1; *SVEP1*, sushi, von Willebrand factor type A, EGF and pentraxin domain containing 1; *SYK*, spleen associated tyrosine kinase; *SYVN1*, synoviolin 1; *TAFA5*, TAFA chemokine like family member 5; *TAT*, tyrosine aminotransferase; *TBX21*, T-box transcription factor 21; *TBXAS1*, thromboxane A synthase 1; *TERF2IP*, TERF2 interacting protein; *TERT*, telomerase reverse transcriptase; *TET2*, tet methylcytosine dioxygenase 2; *TFEB*, transcription factor EB; *TFPI* and *TFPI2*, tissue factor pathway inhibitor and inhibitor 2, respectively; *TGFB1*, transforming growth factor β1; *TGFB1I1*, transforming growth factor β1 induced transcript 1; *TGFBI*, transforming growth factor β induced protein; *TGM2*, transglutaminase 2; *THBD*, thrombomodulin; *THBS2*, thrombospondin 2; *THOC5*, THO complex subunit 5; *TIMP1* and *TIMP3*, TIMP metallopeptidase inhibitors 1 and 3, respectively; *TLN1* and *TLN2*, talins 1 and 2, respectively; *TLR1*, *TLR2*, *TLR4*, *TLR6*, *TLR7*, and *TLR9*, toll like receptors 1, 2, 4, 6, 7, and 9, respectively; *TM4SF5*, transmembrane 4 L six family member 5; *TM6SF2*, transmembrane 6 superfamily member 2; *TMCO1*, transmembrane and coiled-coil domains 1; *TMEM258*, transmembrane protein 258; *TNC*, tenascin C; *TNF*, tumor necrosis factor; *TNFAIP3*, *TNFAIP6*, and *TNFAIP8L1*, TNFα induced proteins 3, 6, and 8 like 1, respectively; *TNFRSF1A*, *TNFSF4*, *TNFRSF9*, *TNFSF10*, *TNFSF11*, *TNFRSF11A*, *TNFRSF11B*, *TNFSF12*, *TNFRSF12A*, *TNFSF13*, *TNFSF13B*,*TNFRSF14*, *TNFSF15*, *TNFRSF18*, and *TNFRSF25*, TNF receptor superfamily members 1A, 4, 9, 10, 11, 11A, 11B, 12, 12A, 13, 13B, 14, 15, 18, and 25, respectively; *TNKS*, tankyrase; *TNNT2*, troponin T2; *TOR2A*, torsin family 2 member A; *TOX*, thymocyte selection associated high mobility group box; *TP53* and *TPD52*, tumor proteins p53 and D52, respectively; *TPM2*, tropomyosin 2; *TPSAB1*, tryptase α/β 1; *TRAF2* and *TRAF6*, TNF receptor associated factors 2 and 6, respectively; *TRAM1*, translocation associated membrane protein 1; *TREM1* and *TREML4*, triggering receptors expressed on myeloid cells 1 and like 4, respectively; *TRHDE*, thyrotropin releasing hormone degrading enzyme; *TRIB3*, tribbles pseudokinase 3; *TRIM7*, tripartite motif containing 7; *TSHB*, thyroid stimulating hormone subunit β; *TSLP*, thymic stromal lymphopoietin; *TSPAN2* and*TSPAN4*, tetraspanin 2 and 4, respectively; *TSPO*, translocator protein; *TTR*, transthyretin; *TUBB4A*, tubulin β 4A class IVa; *TUG1*, taurine up-regulated 1; *TUSC1*, tumor suppressor candidate 1; *TXN*, thioredoxin; *TXNIP*, thioredoxin interacting protein; *TXNL4B*, thioredoxin like 4B; *TXNRD1* and *TXNRD2*, thioredoxin reductases 1 and 2, respectively; *TYRO3*, TYRO3 protein tyrosine kinase; *UBE2I*, ubiquitin conjugating enzyme E2 I; *UCN* and *UCN2*, urocortin and urocortin 2, respectively; *UCP1* and *UCP2*, uncoupling proteins 1 and 2, respectively; *UNC5B*, unc-5 netrin receptor B; *USF1*, upstream transcription factor 1; *USP24*, ubiquitin specific peptidase 24; *UTP20*, UTP20 small subunit processome component; *UTS2* and *UTS2B*, urotensin 2 and 2B, respectively; *UTS2R*, urotensin 2 receptor; *VAMP3*, vesicle associated membrane protein 3; *VCAM1*, vascular cell adhesion molecule 1; *VCL*, vinculin; *VDR*, vitamin D receptor; *VEGFA* and *VEGFC*, vascular endothelial growth factor A and C, respectively; *VIM*, vimentin; *VKORC1*, vitamin K epoxide reductase complex subunit 1; *VLDLR*, very low density lipoprotein receptor; *VTN*, vitronectin; *VWF*, von Willebrand factor; *WDFY4*, WDFY family member 4; *WNT4* and *WNT5A*, Wnt family members 4 and 5A, respectively; *XBP1*, X-box binding protein 1; *XDH*, xanthine dehydrogenase; *YAP1*, Yes1 associated transcriptional regulator; *YWHAZ*, tyrosine 3-monooxygenase/tryptophan 5-monooxygenase activation protein zeta; *ZBTB20*, zinc finger and BTB domain containing 20; *ZC3HC1*, zinc finger C3HC-type containing 1; *ZEB2*, zinc finger E-box binding homeobox 2; *ZFHX3*, zinc finger homeobox 3; *ZMPSTE24*, zinc metallopeptidase STE24; *ZNF202* and *ZNF213*, zinc finger proteins 202 and 213 *YAP1*; *ZPR1*, ZPR1 zinc finger.

International Journal of Molecular Sciences

Table S2. Effects of underexpression or overexpression of the hub genes for atherogenesis, atheroprotection and atherosclerosis to human health according to the current version of the PubMed database [81].

#	Gene (NCBI Entrez ID)	Effect of changes in human gene expression on human health in atherosclerosis, atherogenesis and atheroprotection [Reference]											
		Atherogenesis (ASg)				Atheroprotection (ASp)				Atherosclerosis (AS)			
		underexpression (↓)	ASg	overexpression (↑)	ASg	underexpression (↓)	ASp	overexpression (↑)	ASp	underexpression (↓)	AS	overexpression (↑)	AS
1	*APOA1* (335)	in a human atherosclerosis model using ApoA1-KO mice: reverse cholesterol transport is significantly impaired [116]	→	in a human atherosclerosis model using ApoA1-transgenic mice: ApoA1 excess suppresses angiogenesis, which is necessary for reendothelialization following vascular injury, which may contributes to atherogenicity [117]	→	in a human atherosclerosis model using ApoA1-KO mice: ApoAI dysfunction cannot mediate beneficial changes in atherosclerotic plaques [116]	→	in a human atherosclerosis model using pigs: ApoA1 excess is believed to play an important protective role in the pathogenesis of arteriosclerosis [118]	←	in a human atherosclerosis model using ApoA1-KO mice: the presence of advanced atherosclerotic plaques of the aortic root [116]	→	in a human atherosclerosis model using ApoA1-KO mice: human APOA1 injection pacifies the inflammatory status of plaque macrophages [116]	←
2	*C1QTNF9* (338872)	in human disease models using mice: high-fat diet caused C1QTNF9 downregulation as well as platelet hyper-reactiviation accelerating atherogenesis [124]	→	in human disease models using rats transfected with C1QTNF9: improved vasodilation retarding atherogenesis [121]	←	a cohort-based clinical study [123]: magnesium-based diet is simultaneously reducing C1QTNF9 levels and overweight as atheroprotection	←	in human disease models using rats: exogenous C1QTNF9 upregulates genes with anti-oxidative activities as atheroprotection [120]	←	according to the retrospective analysis of Reactome and GeneCard databases [122]: low C1QTNF9 is one of top-six predictors of high risks of inflammations, which may aggravate atherosclerosis	→	in human disease models using rats with artificial myocardial infarction: injection of adenovirus carrying C1QTNF9 relieved this often complication of atherosclerosis [119]	←
3	*CD163* (9332)	in human disease models using atherosclerotic mice: CD163 deficiency increases foam cell formation and plaque progression [128]	→	cohort-based study: CD163 excess reduced survival of patients with Hodgkin lymphoma [125], which accelerates atherogenesis [126]	→	according to exhaustive review [129]: pro-inflammatory stimuli suppress CD163 in monocytes and macrophages, while low CD163 levels characterize monocyte-derived dendritic cells as well as dendritic cells isolated from the blood	→	according to exhaustive review [129]: anti-inflammatory stimuli induce rapid upregulation of CD163 in monocytes and macrophages, when CD163 displays the strongest upregulation	←	human atherosclerosis model using double ApoE\Cd163-knockout mice: larger plaques with higher lipid and macrophage content as well as pro-inflammatory cytokine excess [128]	→	cohort-based study: CD163 excess reduced survival of patients with Hodgkin lymphoma [125], therapy adainst which may predispose patients to early atherosclerosis [127]	→

.

Table S2 (continued).

#	Gene (NCBI Entrez ID)	Effect of changes in human gene expression on human health in atherosclerosis, atherogenesis and atheroprotection [Reference]											
		Atherogenesis (ASg)				Atheroprotection (ASp)				Atherosclerosis (AS)			
		underexpression (↓)	ASg	overexpression (↑)	ASg	underexpression (↓)	ASp	overexpression (↑)	ASp	underexpression (↓)	AS	overexpression (↑)	AS
4	*CRP* (1401)	according to exhaustive review: lowering plasma CRP levels may reduce atherogenesis [130]	←	according to a retrospective review of clinical data on childhood obesity: excess CRP contributes to atherogenesis [131]	→	cohort-based study of severely obese patients: after gastric partition surgery for weight reduction, CRP level reduced without progress in endothelial cell dysfunction [132]	←	cohort-based study of severely obese patients: before gastric partition surgery for weight reduction, CRP excess may inhibit angiogenesis by inducing endothelial cell dysfunction [132]	→	cohort-based study of patients with atherosclerosis: large/superficial macrophage accumulation and low CRP levels reduced risk of adverse events [133]	←	according to exhaustive review [130]: CRP excess is a biomarker for the atherosclerosis acute-phase as a predictor for future cardiovascular events	→
5	*CXCR4* (7852)	in human disease models using hyperlipidemic mice carrying the CXCR4 deletion in arterial endothelial cells: higher inflammatory leukocyte recruitment in atherogenesis [138]	→	CXCR4 performs proatherogenic functions in various cell types [134]	→	in human disease models using the human umbilical vein endothelial cell line ECV304: suppressed tubule formation, angiogenesis and wound healing [137]	→	CXCR4 performs atheroprotective functions in various cell types [134]	←	knockout *CXCR4* in mice may lead to heart failure [135], which is associated with atherosclerosis [136]	→	according to Human carotid atherosclerosis-related RNA-Seq data meta-analysis: CXCR4 excess is one among 16 hub genes for this disease [139]	→
6	*HMOX1* (3162)	according to exhaustive review: in animal models, a lack of HMOX1 accelerates atherogenesis [142]	→	according to the RNA-Seq data meta-analysis: HMOX1 excess as a biomarker for ferroptosis in atherosclerosis progression [144]	→	in human disease models using mice: HMOX1 inhibition retarded wound healing and impaired angiogenesis [143]	→	in human disease models using Hmox1-transgenic mice: improved wound healing and angiogenesis [143]	←	according to exhaustive review: in animal models, a lack of HMOX1 accelerates atherosclerosis [142]	→	synovial tissue inflammation in rheumatoid arthritis [140] as a model of accelerated atherosclerosis [141]	→
7	*KLF2* (10365)	in human atherosclerosis models using the myeloid-specific Klf2-knockout mice: accelerates atherogenesis [148]	→	according to an exhaustive review: the suppression of blood coagulation and aggregation of macrophages with the vascular endothelium as an atherogenesis prevention [146]	←	in human atherosclerosis models using the endothelial-specific Klf2-knockout mice: higher glucose uptake in endothelial cells of perfused hearts as atheroprotection [149]	←	in a human atheroprotection model using human umbilical vein endothelial cells (HUVECs): inhibited angiogenesis as atheroprotection [145]	←	in a human atherosclerosis model using human bronchial epithelial cells (BEAS-2B): KLF2 silencing provokes an enhanced inflammatory response [147]	→	in human disease models using the atherosclerotic mice: calenduloside E (CE) alleviated atherosclerosis through upregulation of Klf2 expression [150]	←

Table S2 (continued).

#	Gene (NCBI Entrez ID)	Effect of changes in human gene expression on human health in atherosclerosis, atherogenesis and atheroprotection [Reference]											
		Atherogenesis (ASg)				Atheroprotection (ASp)				Atherosclerosis (AS)			
		underexpression (↓)	ASg	overexpression (↑)	ASg	underexpression (↓)	ASp	overexpression (↑)	ASp	underexpression (↓)	AS	overexpression (↑)	AS
8	*LCAT* (3931)	according to exhaustive review: low LCAT expression can often accelerate atherogenesis [152]	→	in human disease models using mice: a novel Lcat-activator DS-8190a prevents atherogenesis [155]	←	according to exhaustive review: age-related decrease in atheroprotective abilities may be associated with inactivation of HDL-associated enzymes, particularly LCAT [156]	→	in human disease models using Lcat-knockout mice: enhanced inflammation, which were reversed by injection of adenovirus carrying *LCAT* gene as atheroprotection [151]	←	a cohort-based clinical study [153]: radiotherapy decreases LCAT reactivity, which contributes to the atherosclerotic complications in breast cancer	→	according to exhaustive review: recombinant human LCAT injections entered clinical phase II trials with good prospects for the treatment of atherosclerosis-related vascular diseases [154]	←
9	*NFE2L2* (4780)	in human disease models using Nrf2-deficient A/J mice: higher oxidative stress [161] as a risk-factor for an accelerated atherogenesis [162]	→	in human smoking-induced atherosclerosis models using rats: Nrf2 upregulation improves endothelial cell viability treated with cigarette smoke extract [163]	←	in human disease models with human coronary arterial endothelial cells with: impaired angiogenesis and ability to form capillary-like structures [157]	→	in human disease models using the human immortalized keratinocyte cells (HaCat): improved wound healing due to alleviated oxidative stress, and decreased apoptosis [158]	←	according to an exhaustive review: NRF2 deficiency in macrophages increases atherosclerosis [159]	→	in human disease models using the atherosclerotic mice: alleviated atherosclerosis due to enhanced autophagy and inhibited ferroptosis [160]	←
10	*NR1H3* (10062)	according to exhaustive review [165]: Nr1h3-knockout mice demonstrated accelerated atherosclerosis	→	according to exhaustive review [167]: NR1H3-agonists suppressed immunity that can slow down atherogenesis	←	in human disease models using Nr1h3-deficient mice: reduced atheroprotection [168]	→	a cohort-based clinical study [164]: NR1H1 level increased as a response to the atherogenesis progress as atheroprotection	←	in human disease models using Nr1h3-knockout mice: more aortic atherosclerotic lesions [166]	→	according to exhaustive review [167]: NR1H3-agonists have anti-inflammatory effects that can alleviate atherosclerosis	←
11	*PF4* (5196)	in a human disease model using PF4-knockout mice: slowed atherogenesis down [172]	←	according to exhaustive review: pro-atherogenic effects [169]	→	a post mortem study of human coronary arteries with atherosclerotic plaques: PF4 and atheroprotector CD163 expression levels correlated negatively with one another [172]	←	according to exhaustive review: anti-angiogenic effects, which can reduce the formation of atherosclerotic plaques [169]	←	a cohort-based clinical study [171]: anti-PF4/heparin IgG excess can provoke thrombosis, which is a complication of atherosclerosis	→	in human disease models using mice injected with recombinant human PF4: induced inflammation [170], which can aggravate atherosclerosis [191]	→

.

Table S2 (continued).

#	Gene (NCBI Entrez ID)	Effect of changes in human gene expression on human health in atherosclerosis, atherogenesis and atheroprotection [Reference]											
		Atherogenesis (ASg)				Atheroprotection (ASp)				Atherosclerosis (AS)			
		underexpression (↓)	ASg	overexpression (↑)	ASg	underexpression (↓)	ASp	overexpression (↑)	ASp	underexpression (↓)	AS	overexpression (↑)	AS
12	*PON1* (5444)	according to an exhaustive review: Pon1-knockout mice as a human disease model showed accelerated atherogenesis [173]	→	according to an exhaustive review: transgenic mice with additional human PON1 gene as a human disease model had an excess of foam cells as a risk factor for atherogenesis [173]	→	in human disease models using rats subjected to high either omega3-fatty acids or alcohol diets: Pon1 deficiency lowering atheroprotective abilities, which may be restored using a drug betaine [177]	→	in human disease models using transgenic mice carrying human PON1 gene: improved efflux, reverse transport and homeostasis of cholesterol as atheroprotection [175]	←	in human disease models using double ApoE/Pon1 knockout mice: severe age-dependent atherosclerosis aggravating upto Alzheimer's disease [176]	→	according to an exhaustive review: an increase in PON1 activity would be expected to decrease inflammation and atherosclerosis [174]	←
13	*PON2* (5445)	in human disease models using Pon2-knockout mice: accelerated atherogenesis [178]	→	in human disease models using mouse macrophages J774A.1 cell line: pomegranate juice slows atherogenesis due to stimulating an additional PON2 expression in macrophage [182]	←	in human disease models using human endothelial EAhy926 cells under RNAi-mediated Pon2-knockdown: vast reactive oxygen species formation [179] as atheroprotection [180, 206]	←	according to an exhaustive review: PON2 expression level appear to be higher in female mice compared with male ones that leads an increased atheroprotection in female mice [181]	←	in human disease models using Pon2-knockout mice: vascular inflammation, blood coagulation abnormalities, increased oxidative stress and endothelial dysfunction that worsen atherosclerosis [178]	→	according to an exhaustive review: PON2 overexpression decreases atherosclerotic lesions [181]	←
14	*SERPINF1* (5176)	according to exhaustive review [187]: SERPINF1 deficiency can lead to increased microvessel density and more thicker walled blood vessels that can slow atherogenesis down	←	in human atherogenesis models using aged vascular smooth muscle cells: SERPINF1 excess is a biomarker for atherosclerotic plaque [185]	→	a cohort-based clinical study: SERPINF1 deficiency is a biomarker for angiogenesis [186], which contributes atheroprotection	←	according to exhaustive review [183]: blocked regeneration in human wound-healing models using mice [184]	→	in human disease models using the SERPINF1-deficient mice: aggravated atherosclerosis [188]	→	in polycystic ovary syndrome: controlled inflammation [189] via inhibition [190], when atherosclerosis is a chronic inflammation [191]	←
15	*TLR2* (7097)	in a human disease model using Tlr2-knockout mice: too few foam cells, the formation of which is a key event in early atherogenesis [199]	←	in human atherosclerosis models using macrophage cell lines: exogenous TLR2 reduces as a potential therapeutic preventer of atherogenesis [195]	←	in a human atherogenesis model using Tlr2-knockout mice: a reduced accumulation of lipids, foam cells, and endothelial cell injuries [197]	←	in a human disease model using microvascular endothelial cells: TLR2 excess promotes angiogenesis and cell adhesion [198]	←	in human disease models using Tlr2-deficient mice: heightened inflammation [196]	→	according to a microarray data meta-analysis: TLR2 excess may have a crucial role in progressing from coronary atherosclerosis to myocardial infarction [200]	→

.

Table S2 (continued).

#	Gene (NCBI Entrez ID)	Effect of changes in human gene expression on human health in atherosclerosis, atherogenesis and atheroprotection [Reference]											
		Atherogenesis (ASg)				Atheroprotection (ASp)				Atherosclerosis (AS)			
		underexpression (↓)	ASg	overexpression (↑)	ASg	underexpression (↓)	ASp	overexpression (↑)	ASp	underexpression (↓)	AS	overexpression (↑)	AS
16	YAP1 (10413)	in human disease models using human umbilical vein endothelial cells (HUVECs): methotrexate exerts atheroprotective effects due to YAP1 downregulation [203]	←	according to an exhaustive review: inhibited apoptosis [208] that can retard atherogenesis [209]	←	in human disease models using Yap1-deficient mice: loss of atheroprotective alignment of vascular endothelial cells in the direction of blood flow [204]	→	in human disease models using human colorectal cancer SW48 cell line: YAP1 overexpression is accompanied by an increase in reactive oxygen species generation [205] as atheroprotection [180, 206]	←	in human disease models using atherosclerotic mice with endothelial-cell-specific Yap1-excess: harmin (a natural extract from *Peganum harmala*) reduces Yap1 activity that relieves atherosclerosis [207]	←	according to an exhaustive review: promoted angiogenesis [201] can contribute to atherosclerotic plaque instability, which can increase cardio-cerebrovascular disease risks [202]	→

***Note*:** see the footnote of Table S1. Changes in gene expression: underexpression (↓) or overexpression (↑); *ASg*, AS, and ASp as effects on human health in atherogenesis, atheroprotection and atherosclerosis, respectively: aggravating (→) or alleviating (←).

International Journal of
Molecular Sciences

Section S1. Supplementary methods for DNA sequence analysis

For each pair of ancestral (WT) and minor (MIN) alleles of each human SNP considered, two DNA sequences $S_{WT}=\{s_{WT;-90}\ldots s_{WT;i}\ldots s_{WT;-1}\}$ and $S_{MIN}=\{s_{MIN;-90}\ldots s_{MIN;i}\ldots s_{MIN;-1}\}$, each 90 bp in length, which textually represent these alleles of the corresponding promoter located upstream of the human gene transcription start site (TSS, $s_{WT;0}=s_{MIN;0}$; $s_i \in \{a, c, g, t\}$) were used as input data to our previously developed web service SNP_TATA_Comparator in automatic mode [88].

First, for each of these sequences $S\in\{S_{WT}, S_{MIN}\}$, the value "-ln(K_D(S))" of the TATA-binding protein (TBP) binding affinity for the corresponding variant of this promoter was calculated using our three-step model, confirmed experimentally [80] and used independently in a clinical study of pulmonary tuberculosis [227]:

$$-\ln(K_D) = 10.9 - 0.2\ [\ln(K_{SLIDE}\ K_{STOP}\ K_{BEND}), \qquad \textbf{(1)}$$

where 10.9 (ln units) corresponds to the estimates of non-specific TBP-DNA affinity (10 mM [224]); 0.2 is the stoichiometric coefficient [72]; $-\ln(K_{STOP})$ is the empirical estimate of the affinity of the TBP for the best TBP-binding site [76] by use of Bucher's position-weight matrix [75] among all possible 15-bp fragments of both DNA chains of the promoter being analyzed, as:

$$\boldsymbol{ln(K_{STOP})} = MAX\left\{\sum_{j=-1}^{13} w_{j;s_{i+j}}\right\}, \qquad (2)$$

where w_{js} as an element of Bucher's matrix [75], which corresponds to the case of the nucleotide s located at the *j*-th position of the DNA sequence in question; MAX(ζ) is the highest ζ-value found.

In Eq. (1), $-\ln(K_{SLIDE})$ is the empirical estimate of TBP affinity for this promoter during TBP sliding along DNA in the ±5-bp local environment around the best TBP-binding site in question [73], which is heuristically estimated as

$$-\ln(K_{SLIDE}) = \textbf{MEAN}\ \{0.8[TA] + 3.4\mu + 35.1\}, \qquad \textbf{(3)}$$

where [TA] is the weighted number of dinucleotide TA; μ is the arithmetic mean of the minor groove width of the DNA helix [225] of the best TBP-binding site considered; 0.8, 3.4, and 35.1 are the linear regression coefficients estimeted elsewhere [226]; MEAN(ζ) is the arithmetic mean of all observed ζ-values.

In Eq. (1), $-\ln(K_{BEND})$ is the *in silico* estimate of TBP affinity for the best TBP-binding site under study during allosteric rearrangement [77] of the B-helical DNA of this site by bending its axis at right angles to stabilize the TBP-promoter complex [78, 79]:

$$-\ln(K_{BEND}) = \textbf{MEAN}\{0.9[TA, AA, TG, AG] + 2.5[TA, TC, TG] +14.4\}, \qquad \textbf{(4)}$$

where 0.9, 2.5, and 14.4 are the linear regression coefficients as described elsewhere [226].

Next, the "-ln[K_D]" values (Eqs. 1-4) go together with their standard errors of the mean (SEM) calculated according to all possible nucleotide substitutions, $s_{\bullet;j}\rightarrow\xi$, at each *j*-th position within the same ±5-bp local environment around the most probable TBP-binding site, namely:

$$SEM(S_\bullet)=[\ (\Sigma_{1\le i\le 26}\Sigma_{\xi \circledcirc \{a,c,g,t\}}[\ln(K_D(\{s_{\bullet;i-13}\ldots\xi\ldots\bullet s_{\bullet;i+12}\})/K_D(\{s_{\bullet;i-13}\ldots s_{\bullet;i+j}\ldots s_{\bullet;i+12}\})^2)]/(3*26)^{1/2} \qquad \textbf{(5)}$$

As an intermediate result, there are two paired estimates, "$-\ln(K_D(S_{WT})) \pm SEM(S_{WT})$" and "$-\ln(K_D(S_{MIN}))\pm SEM(S_{MIN})$", calculated with the input sequences S_{WT} and S_{MIN} using Eqs. (1-5), which are statistically comparable according to Fisher's Z-test:

$$Z = \textbf{abs}\ [\ln(K_{WT;D} / K_{MIN;D})] / [SEM(S_{WT})^2 + SEM(S_{MIN})^2]^{1/2}, \qquad (6)$$

where Z is Fisher's Z-score as input for the corresponding procedure within the statistical package R [89], the output is the *p*-value of the probability rate of acceptance of the H_0-hypothesis "H_0: $K_D(S_{WT}) \neq K_D(S_{MIN})$".

Finally, the desired decision is made at its statistically significant level $\alpha < 0.05$ (where $\alpha = 1 - p$):

IF {*INEQUALITY* "$-\ln(K_{WT;D}) > -\ln(K_{MIN;D})$" is statistically significant},

THEN {*DECISION* is "S_{MIN} provides an underexpression of a given gene in comparison with S_{WT} as a norm"};

ELSE [**IF** {*INEQUALITY* "$-\ln(K_{WT;D}) < -\ln(K_{MIN;D})$" is statistically significant},

THEN {*DECISION* is "S_{MIN} provides an overexpression of a given gene in comparison with S_{WT} as a norm"},

OTHERWISE {*DECISION* is "change in the expression of this gene is insignificant"}.

The column "Δ" in Table S3 shows this DECISION as "↑" (overexpression) and "↓" (underexpression).

International Journal of *Molecular Sciences*

MDPI

Table S3. Candidate SNP markers within the 90-bp proximal promoters of 16 human hub genes for atherogenesis, atheroprotection and atherosclerosis according to *in silico* analysis.

Gene		Candidate SNP marker				K_D, nM, MEAN ± SEM, *in silico*					Effect on human health during atherosclerosis, atherogenesis and atheroprotection [Ref]					
#	Entrez ID	dbSNP ID:min [36]	5′ flank, 10 bp	wt<br>min	3 flank, 10 bp	wt<br>min	Δ	Z	p	ϱ	Atherogenesis	ASg	Atheroprotection	ASp	Atherosclerosis	AS
1	*APOA1* (335)	rs1428975217:T	acataaatag	G<br>T	ccctgcaaga	2.57±0.28<br>3.01±0.30	↓	2.26	0.05	D	in a human atherosclerosis model using ApoA1-KO mice: reverse cholesterol transport is significantly impaired [116]	→	in a human atherosclerosis model using ApoA1-KO mice: ApoAI dysfunction cannot mediate beneficial changes in atherosclerotic plaques [116]	→	in a human atherosclerosis model using ApoA1-KO mice: the presence of advanced atherosclerotic plaques of the aortic root [116]	→
		rs1941589135:G	agacataaat	A<br>G	ggccctgcaa	2.57±0.28<br>3.68±0.33	↓	5.15	10^{-6}	A						
		rs1017922094:C	cagacataaa	T<br>C	aggccctgca	2.57±0.28<br>5.55±0.72	↓	9.14	10^{-6}	A						
		rs1217664818:G	gctggctgct	T<br>G	agagactgcg	20.15±2.02<br>49.56±3.47	↓	14.85	10^{-6}	A						
		rs563005045:A	gctgcttaga	G<br>A	actgcgagaa	20.15±2.02<br>11.17±1.01	↑	8.65	10^{-6}	A	in a human atherosclerosis model using ApoA1-transgenic mice: ApoA1 excess suppresses angiogenesis, which is necessary for reendothelialization following vascular injury, which may contributes to atherogenicity [117]	→	in a human atherosclerosis model using pigs: ApoA1 excess is believed to play an important protective role in the pathogenesis of arteriosclerosis [118]	←	in a human atherosclerosis model using ApoA1-KO mice: human APOA1 injection pacifies the inflammatory status of plaque macrophages [116]	←
		rs1356180102:T	gagctggctg	C<br>T	ttagagactg	20.15±2.02<br>12.10±1.57	↑	6.14	10^{-6}	A						
2	*C1QTNF9* (338872)	rs1593529423:T	ggctgatttt	A<br>T	tttagctggc	4.59±0.41<br>9.91±0.79	↓	13.32	10^{-6}	A	in human disease models using mice: high-fat diet caused C1QTNF9 downregulation as well as platelet hyper-reactiviation accelerating atherogenesis [124]	→	a cohort-based clinical study [123]: magnesium-based diet is simultaneously reducing C1QTNF9 levels and overweight as atheroprotection	←	according to the retrospective analysis of Reactome and GeneCard databases [122]: low C1QTNF9 is one of top-six predictors of high risks of inflammations, which may aggravate atherosclerosis	→
		rs1247398555:A	gcgggggctt	C<br>A	tgggatttgt	6.98±0.70<br>8.44±0.84	↓	2.83	10^{-2}	C						
		rs1877629162:T	ggatttgtag	C<br>T	tcctctgctg	6.98±0.70<br>5.38±0.54	↑	3.85	10^{-3}	B	in human disease models using rats transfected with C1QTNF9: improved vasodilation retarding atherogenesis [121]	←	in human disease models using rats: exogenous C1QTNF9 upregulates genes with anti-oxidative activities as atheroprotection [120]	←	in human disease models using rats with artificial myocardial infarction: injection of adenovirus carrying C1QTNF9 relieved this often complication of atherosclerosis [119]	←

Table S3 (continued).

	Gene	Candidate SNP marker				K_D, nM, MEAN ± SEM, *in silico*					Effect on human health during atherosclerosis, atherogenesis and atheroprotection [Ref]					
#	Entrez ID	dbSNP ID:min [36]	5′ flank, 10 bp	wt min	3 flank, 10 bp	wt min	Δ	Z	p	ϱ	Atherogenesis	ASg	Atheroprotection	ASp	Atherosclerosis	AS
3	*CD163* (9332)	rs1410411790:G	ccgcctccat	C G	tgtagccttt	29.76±2.08 33.22±2.66	↓	1.97	0.05	D	in human disease models using atherosclerotic mice: CD163 deficiency increases foam cell formation and plaque progression [128]	→	according to exhaustive review [129]: pro-inflammatory stimuli suppress CD163 in monocytes and macrophages, while low CD163 levels characterize monocyte-derived dendritic cells as well as dendritic cells isolated from the blood	→	human atherosclerosis model using double ApoE\Cd163-knockout mice: larger plaques with higher lipid and macrophage content as well as pro-inflammatory cytokine excess [128]	→
		rs1260721214:G	ccgcctccat	A G	tgtagccttt	3.36±0.34 10.21±0.92	↓	16.19	10^{-6}	A						
		rs759789809:G	ccgcctccat	A G	tgtagccttt	3.36±0.34 5.95±0.65	↓	7.42	10^{-6}	A						
		rs1375903774:G	aggttgccta	T G	aaacatgaac	3.43±0.41 17.00±1.70	↓	19.70	10^{-6}	A						
		rs1949512139:A	ctaggttgcc	T A	ataaacatga	3.43±0.41 8.96±0.99	↓	11.62	10^{-6}	A						
		rs1442255159:G	cctgggggac	A G	gtgtgtgatg	23.65±2.13 32.24±2.58	↓	5.24	10^{-6}	A						
		rs1949529239:A	atttcctttg	G A	ttgtttcatg	29.76±2.08 20.97±1.47	↑	7.22	10^{-6}	A	cohort-based study: CD163 excess reduced survival of patients with Hodgkin lymphoma [125], which accelerates atherogenesis [126]	→	according to exhaustive review [129]: anti-inflammatory stimuli induce rapid upregulation of CD163 in monocytes and macrophages, when CD163 displays the strongest upregulation	←	cohort-based study: CD163 excess reduced survival of patients with Hodgkin lymphoma [125], therapy adainst which may predispose patients to early atherosclerosis [127]	→
		rs1949529809:A	cgaaatctgt	G A	ggaagtgttt	29.76±2.08 19.36±1.74	↑	7.74	10^{-6}	A						
		rs775317096:A	tggacgtgtg	G A	agatctggca	29.76±2.08 15.85±1.27	↑	11.70	10^{-6}	A						
		rs775317096:T	tggacgtgtg	G T	agatctggca	29.76±2.08 11.17±1.01	↑	17.25	10^{-6}	A						
		rs982015915:T	tctaattttt	C T	gaaatctgtg	29.76±2.08 25.62±1.79	↑	2.94	10^{-2}	C						
		rs1949526115:A	gcctccatat	G A	tagccttttc	3.36±0.34 1.23±0.15	↑	12.93	10^{-6}	A						
		rs1212635706:A	tccgcctcca	G A	atgtagcctt	3.36±0.34 7.79±0.78	↑	11.80	10^{-6}	A						
		rs1949193000:T	ttcaaagaag	C T	agagtttggt	25.11±1.76 12.85±1.29	↑	10.83	10^{-6}	A						
		rs763374425:A	attcaaagaa	G A	cagagtttgg	25.11±1.76 18.98±1.52	↑	5.24	10^{-6}	A						
		rs771413158:A	tgaaagcatt	C A	aaagaagcag	25.11±1.76 6.25±0.44	↑	28.31	10^{-6}	A						
		rs1424881467:C	tttgaaagca	T C	tcaaagaagc	25.11±1.76 22.27±1.56	↑	2.45	0.05	D						
		rs774603375:A	gtccagcttt	G A	aaagcattca	25.11±1.76 6.51±0.59	↑	24.19	10^{-6}	A						

Table S3 (continued).

Gene		Candidate SNP marker				K_D, nM, MEAN ± SEM, *in silico*					Effect on human health during atherosclerosis, atherogenesis and atheroprotection [Ref]					
#	Entrez ID	dbSNP ID:min [36]	5′ flank, 10 bp	wt min	3 flank, 10 bp	wt min	Δ	Z	p	ϱ	Atherogenesis	ASg	Atheroprotection	ASp	Atherosclerosis	AS
3	*CD163* (9332)	rs774318960:T	tgagactgac	C T	agtgaagcca	48.10±3.37 14.63±1.32	↑	21.44	10^{-6}	A	cohort-based study: CD163 excess reduced survival of patients with Hodgkin lymphoma [125], which accelerates atherogenesis [126]	→	according to exhaustive review [129]: anti-inflammatory stimuli induce rapid upregulation of CD163 in monocytes and macrophages, when CD163 displays the strongest upregulation	←	cohort-based study: CD163 excess reduced survival of patients with Hodgkin lymphoma [125], therapy adainst which may predispose patients to early atherosclerosis [127]	→
		rs1357215526:A	cctctaggtt	G A	cctataaaca	3.43±0.41 2.90±0.29	↑	2.20	0.05	D						
		rs1397676177:T	gttcctgggg	G T	acagtgtgtg	23.65±2.13 13.64±0.95	↑	9.61	10^{-6}	A						
		rs1949194990:A	gtgtggagat	C A	tggcatggag	23.65±2.13 16.83±1.35	↑	5.76	10^{-6}	A						
		rs1206049755:T	tggacgtgtg	G T	agatctggca	23.65±2.13 9.33±0.75	↑	15.30	10^{-6}	A						
		rs779801921:A	ttctggacgt	G A	tggagatctg	23.65±2.13 15.08±1.36	↑	7.25	10^{-6}	A						
4	*CRP* (1401)	rs1660782424:C	gctttggata	T C	aaatccaggc	2.26±0.23 6.19±0.62	↓	13.78	10^{-6}	A	according to exhaustive review: lowering plasma CRP levels may reduce atherogenesis [130]	←	cohort-based study of severely obese patients: after gastric partition surgery for weight reduction, CRP level reduced without progress in endothelial cell dysfunction [132]	←	cohort-based study of patients with atherosclerosis: large/superficial macrophage accumulation and low CRP levels reduced risk of adverse events [133]	←
		rs1660782480:G	tgctttggat	A G	taaatccagg	2.26±0.23 7.64±0.76	↓	16.76	10^{-6}	A						
5	*CXCR4* (7852)	rs575012225:G	gtgtttttat	A G	aaagtccggc	2.00±0.20 4.73±0.43	↓	12.99	10^{-6}	A	in human disease models using hyperlipidemic mice carrying the CXCR4 deletion in arterial endothelial cells: higher inflammatory leukocyte recruitment in atherogenesis [138]	→	in human disease models using the human umbilical vein endothelial cell line ECV304: suppressed tubule formation, angiogenesis and wound healing [137]	→	knockout *CXCR4* in mice may lead to heart failure [135], which is associated with atherosclerosis [136]	→
		rs1573617455:G	gcgtgttttt	A G	taaaagtccg	2.00±0.20 5.28±0.48	↓	14.46	10^{-6}	A						
		rs1257234332:T	ctttgtgtgt	A T	tttttttttt	7.49±0.60 11.63±0.81	↓	8.44	10^{-6}	A						
		rs1257234332:G	ctttgtgtgt	A G	tttttttttt	7.49±0.60 9.24±0.65	↓	4.00	10^{-3}	B						
		rs1233429125:G	cctctttgtg	T G	gtattttttt	7.49±0.60 14.63±1.17	↓	12.13	10^{-6}	A						
		rs1684886719:G	agtggcctct	T G	tgtgtgtatt	7.49±0.60 14.34±1.29	↓	10.94	10^{-6}	A						
		rs1184026217:T	ttgcaaacgt	A T	gtgcgggatt	28.03±2.24 31.60±2.53	↓	2.00	0.05	D						

Table S3 (continued).

Gene		Candidate SNP marker				K_D, nM, MEAN ± SEM, *in silico*					Effect on human health during atherosclerosis, atherogenesis and atheroprotection [Ref]					
#	Entrez ID	dbSNP ID:min [36]	5′ flank, 10 bp	wt<br>min	3 flank, 10 bp	wt<br>min	Δ	Z	p	ϱ	Atherogenesis	ASg	Atheroprotection	ASp	Atherosclerosis	AS
5	*CXCR4* (7852)	rs1684954628:C	tttgcaaacg	T<br>C	aagaacattc	28.03±2.24<br>35.99±2.52	↓	4.70	10^{3}	B	in human disease models using hyperlipidemic mice carrying the CXCR4 deletion in arterial endothelial cells: higher inflammatory leukocyte recruitment in atherogenesis [138]	→	in human disease models using the human umbilical vein endothelial cell line ECV304: suppressed tubule formation, angiogenesis and wound healing [137]	→	knockout *CXCR4* in mice may lead to heart failure [135], which is associated with atherosclerosis [136]	→
		rs576441901:T	tgtttgcaaa	C<br>T	gtaagaacat	28.03±2.24<br>33.22±2.66	↓	3.01	10^{2}	C						
		rs576441901:G	tgtttgcaaa	C<br>G	gtaagaacat	28.03±2.24<br>33.22±2.33	↓	3.10	10^{2}	C						
		rs1684955019:G	ctgtttgcaa	A<br>G	cgtaagaaca	28.03±2.24<br>35.99±2.52	↓	4.72	10^{3}	B						
		rs1684955088:C	gctgtttgca	A<br>C	acgtaagaac	28.03±2.24<br>35.63±2.85	↓	4.09	10^{3}	B						
		rs1277424593:C	cgctgtttgc	A<br>C	aacgtaagaa	28.03±2.24<br>57.59±4.03	↓	12.99	10^{6}	A						
		rs17848384:G	acgctgtttg	C<br>G	aaacgtaaga	28.03±2.24<br>37.09±2.60	↓	5.34	10^{6}	A						
		rs1684955887:C	tgcacgctgt	T<br>C	tgcaaacgta	28.03±2.24<br>36.35±2.91	↓	4.54	10^{3}	B						
		rs139877430:T	ttttctgaca	C<br>T	tcccgcccaa	25.87±1.81<br>28.60±2.00	↓	2.03	0.05	D						
		rs1283131178:C	gttcattttc	T<br>C	gacactcccg	25.87±1.81<br>28.60±2.00	↓	2.03	0.05	D						
		rs748060710:G	catgcctat	T<br>G	agtgcgggtg	6.84±1.09<br>14.49±1.30	↓	8.31	10^{6}	A						
		rs748060710:C	catgcctat	T<br>C	agtgcgggtg	6.84±1.09<br>14.49±1.30	↓	8.31	10^{6}	A						
		rs1684961935:C	accatgccta	T<br>C	atagtgcggg	6.84±1.09<br>13.11±1.57	↓	6.54	10^{6}	A						
		rs1684962035:G	tgaccatgcc	T<br>G	atatagtgcg	6.84±1.09<br>14.49±1.30	↓	8.31	10^{6}	A						
		rs1684998884:G	gcccagttct	T<br>G	caacctaatt	15.23±1.07<br>22.95±1.61	↓	8.32	10^{6}	A						

Table S3 (continued).

Gene		Candidate SNP marker				K_D, nM, MEAN ± SEM, *in silico*					Effect on human health during atherosclerosis, atherogenesis and atheroprotection [Ref]					
#	Entrez ID	dbSNP ID:min [36]	5′ flank, 10 bp	wt<br>min	3 flank, 10 bp	wt<br>min	Δ	Z	p	ϱ	Atherogenesis	ASg	Atheroprotection	ASp	Atherosclerosis	AS
5	*CXCR4* (7852)	rs1684974170:G	tgtttttata	A<br>G	aagtccggcc	2.00±0.20<br>1.70±0.17	↑	2.38	0.05	D	CXCR4 performs proatherogenic functions in various cell types [134]	→	CXCR4 performs atheroprotective functions in various cell types [134]	←	according to Human carotid atherosclerosis-related RNA-Seq data meta-analysis: CXCR4 excess is one among 16 hub genes for this disease [139]	→
		rs902195703:A	gagcgtgttt	T<br>A	tataaaagtc	2.00±0.20<br>1.13±0.12	↑	7.74	10^{-6}	A						
		rs988917830:A	gcctctttgt	G<br>A	cgggtgggtg	7.49±0.60<br>3.87±0.39	↑	10.36	10^{-6}	A						
		rs561969013:T	tggcctcttt	G<br>T	tgtgtatttt	7.49±0.60<br>6.01±0.48	↑	3.80	10^{-3}	B						
		rs1684886932:C	cgcctgggag	T<br>C	ggcctctttg	7.49±0.60<br>6.64±0.53	↑	2.02	0.05	D						
		rs1277424593:T	cgctgtttgc	A<br>T	aacgtaagaa	28.03±2.24<br>23.65±1.89	↑	3.22	10^{-2}	C						
		rs17848384:T	acgctgtttg	C<br>T	aaacgtaaga	28.03±2.24<br>5.02±0.50	↑	27.60	10^{-6}	A						
		rs559116070:T	ggcttgcacg	C<br>T	tgtttgcaaa	28.03±2.24<br>22.49±2.02	↑	3.83	10^{-3}	B						
		rs1406216428:A	cggcttgcac	G<br>A	ctgtttgcaa	28.03±2.24<br>23.41±1.87	↑	3.30	10^{-3}	B						
		rs572642853:T	cggcggcttg	C<br>T	acgctgtttg	28.03±2.24<br>17.00±1.70	↑	7.89	10^{-6}	A						
		rs1684956329:A	gcggcggctt	G<br>A	cacgctgttt	28.03±2.24<br>14.78±1.48	↑	10.32	10^{-6}	A						
		rs1349470636:A	tctcccctt	G<br>A	agtcccgccg	28.03±2.24<br>14.93±1.94	↑	8.59	10^{-6}	A						
		rs1341246266:A	ttcattttct	G<br>A	acactcccgc	25.87±1.81<br>8.88±0.71	↑	21.08	10^{-6}	A						
		rs1434964208:A	tgccgtttgt	T<br>A	cattttctga	25.87±1.81<br>8.61±0.86	↑	17.97	10^{-6}	A						
		rs1310437977:C	tatagtgcgg	G<br>C	tgggtggggg	6.84±1.09<br>1.88±0.36	↑	10.58	10^{-6}	A						
		rs1684961410:A	atatagtgcg	G<br>A	gtgggtgggg	6.84±1.09<br>1.88±0.34	↑	10.85	10^{-6}	A						
		rs1457619557:T	tatatagtgc	G<br>T	ggtgggtggg	6.84±1.09<br>1.67±0.27	↑	12.55	10^{-6}	A						
		rs1457619557:C	tatatagtgc	G<br>C	ggtgggtggg	6.84±1.09<br>1.88±0.36	↑	10.52	10^{-6}	A						

Table S3 (continued).

Gene		Candidate SNP marker				K_D, nM, MEAN ± SEM, *in silico*					Effect on human health during atherosclerosis, atherogenesis and atheroprotection [Ref]					
#	Entrez ID	dbSNP ID:min [36]	5′ flank, 10 bp	wt<br>min	3 flank, 10 bp	wt<br>min	Δ	Z	p	ϱ	Atherogenesis	ASg	Atheroprotection	ASp	Atherosclerosis	AS
5	*CXCR4* (7852)	rs1179591969:G	ctatatagtg	C<br>G	gggtgggtgg	6.84±1.09<br>1.88±0.36	↑	10.50	10^{6}	A	CXCR4 performs proatherogenic functions in various cell types [134]	→	CXCR4 performs atheroprotective functions in various cell types [134]	←	according to Human carotid atherosclerosis-related RNA-Seq data meta-analysis: CXCR4 excess is one among 16 hub genes for this disease [139]	→
		rs1179591969:A	ctatatagtg	C<br>A	gggtgggtgg	6.84±1.09<br>1.72±0.31	↑	11.59	10^{6}	A						
		rs1684961578:A	cctatatagt	G<br>A	cgggtgggtg	6.84±1.09<br>1.72±0.26	↑	12.47	10^{6}	A						
		rs1684961732:C	tgcctatata	G<br>C	tgcgggtggg	6.84±1.09<br>1.69±0.22	↑	13.55	10^{6}	A						
		rs1684961732:A	tgcctatata	G<br>A	tgcgggtggg	6.84±1.09<br>5.44±0.49	↑	2.50	0.05	D						
		rs1172538765:G	atgcctatat	A<br>G	gtgcgggtgg	6.84±1.09<br>4.82±0.63	↑	3.41	10^{3}	B						
		rs979610732:T	ttgaccatgc	C<br>T	tatatagtgc	6.84±1.09<br>1.18±0.14	↑	17.56	10^{6}	A						
		rs925113836:T	cttgaccatg	C<br>T	ctatatagtg	6.84±1.09<br>4.03±0.52	↑	5.20	10^{6}	A						
		rs1308844840:T	tcttgaccat	G<br>T	cctatatagt	6.84±1.09<br>5.17±0.52	↑	2.92	10^{2}	C						
		rs1308844840:A	tcttgaccat	G<br>A	cctatatagt	6.84±1.09<br>2.39±0.22	↑	11.51	10^{6}	A						
		rs1684962267:C	ttcttgacca	T<br>C	gcctatatag	6.84±1.09<br>1.76±0.26	↑	12.42	10^{6}	A						
		rs530036047:G	tttcttgacc	A<br>G	tgcctatata	6.84±1.09<br>1.87±0.26	↑	12.24	10^{6}	A						
		rs530036047:T	tttcttgacc	A<br>T	tgcctatata	6.84±1.09<br>1.67±0.28	↑	12.11	10^{6}	A						
6	*HMOX1* (3162)	rs912562278:A	ccgccgagca	T<br>A	aaatgtgacc	4.77±0.43<br>21.40±1.28	↓	28.15	10^{6}	A	according to exhaustive review: in animal models, a lack of HMOX1 accelerates atherogenesis [142]	→	in human disease models using mice: HMOX1 inhibition retarded wound healing and impaired angiogenesis [143]	→	according to exhaustive review: in animal models, a lack of HMOX1 accelerates atherosclerosis [142]	→
		rs1015512725:G	cccgccgagc	A<br>G	taaatgtgac	4.77±0.43<br>9.05±0.81	↓	10.35	10^{6}	A						
		rs1015512725:T	cccgccgagc	A<br>T	taaatgtgac	4.77±0.43<br>7.56±0.60	↓	7.75	10^{6}	A						
		rs1931345862:C	gcagtttgta	T<br>C	gacaggtgtc	10.63±1.06<br>16.50±1.48	↓	6.53	10^{6}	A						

Table S3 (continued).

#	Gene	Candidate SNP marker				K_D, nM, MEAN ± SEM, *in silico*					Effect on human health during atherosclerosis, atherogenesis and atheroprotection [Ref]					
#	Entrez ID	dbSNP ID:min [36]	5′ flank, 10 bp	wt<br>min	3 flank, 10 bp	wt<br>min	Δ	Z	p	ϱ	Atherogenesis	ASg	Atheroprotection	ASp	Atherosclerosis	AS
6	*HMOX1* (3162)	rs1931345778:G	agcagtttgt	A<br>G	tgacaggtgt	10.63±1.06<br>33.89±3.39	↓	16.83	10^{-6}	A	according to exhaustive review: in animal models, a lack of HMOX1 accelerates atherogenesis [142]	→	in human disease models using mice: HMOX1 inhibition retarded wound healing and impaired angiogenesis [143]	→	according to exhaustive review: in animal models, a lack of HMOX1 accelerates atherosclerosis [142]	→
		rs1931345778:T	agcagtttgt	A<br>T	tgacaggtgt	10.63±1.06<br>29.47±2.06	↓	16.59	10^{-6}	A						
		rs1931345708:C	aagcagtttg	T<br>C	atgacaggtg	10.63±1.06<br>33.89±3.39	↓	16.83	10^{-6}	A						
		rs1931345609:C	gaagcagttt	G<br>C	tatgacaggt	10.63±1.06<br>13.11±1.18	↓	3.15	10^{-2}	C						
		rs1428247265:C	cccaggattt	G<br>C	tcagaggccc	25.87±2.07<br>42.24±3.38	↓	8.90	10^{-6}	A						
		rs962648625:A	gagcataaat	G<br>A	tgaccggccg	4.77±0.43<br>3.68±0.29	↑	4.19	10^{-3}	B	according to the RNA-Seq data meta-analysis: HMOX1 excess as a biomarker for ferroptosis in atherosclerosis progression [144]	→	in human disease models using Hmox1-transgenic mice: improved wound healing and angiogenesis [143]	←	synovial tissue inflammation in rheumatoid arthritis [140] as a model of accelerated atherosclerosis [141]	→
		rs1201270836:A	cagtttgtat	G<br>A	acaggtgtcc	10.63±1.06<br>2.81±0.31	↑	18.04	10^{-6}	A						
		rs1601739465:T	aggatttgtc	A<br>T	gaggccctga	25.87±2.07<br>20.76±1.66	↑	4.02	10^{-3}	B						
		rs369762159:A	gccccaggat	T<br>A	tgtcagaggc	25.87±2.07<br>8.19±0.90	↑	17.38	10^{-6}	A						
7	*KLF2* (10365)	rs2091882204:G	cgccgcgcct	A<br>G	taaggcttgg	7.19±0.86<br>21.83±2.40	↓	13.28	10^{-6}	A	in human atherosclerosis models using the myeloid-specific Klf2-knockout mice: accelerates atherogenesis [148]	→	in human atherosclerosis models using the endothelial-specific Klf2-knockout mice: higher glucose uptake in endothelial cells of perfused hearts as atheroprotection [149]	←	in a human atherosclerosis model using human bronchial epithelial cells (BEAS-2B): KLF2 silencing provokes an enhanced inflammatory response [147]	→
		rs2091882204:C	cgccgcgcct	A<br>C	taaggcttgg	7.19±0.86<br>29.17±2.63	↓	18.38	10^{-6}	A						
		rs2091882227:A	cgcctataag	G<br>A	cttgggcggg	7.19±0.86<br>4.92±0.59	↑	4.36	10^{-3}	B	according to an exhaustive review: the suppression of blood coagulation and aggregation of macrophages with the vascular endothelium as an atherogenesis prevention [146]	←	in a human atheroprotection model using human umbilical vein endothelial cells (HUVECs): inhibited angiogenesis as atheroprotection [145]	←	in human disease models using the atherosclerotic mice: calenduloside E (CE) alleviated atherosclerosis through upregulation of Klf2 expression [150]	←
		rs1231237897:C	gcgcctataa	G<br>C	gcttgggcgg	7.19±0.86<br>5.77±0.58	↑	2.80	10^{-2}	C						
		rs1231237897:A	gcgcctataa	G<br>A	gcttgggcgg	7.19±0.86<br>4.23±0.55	↑	5.87	10^{-6}	A						

Table S3 (continued).

Gene		Candidate SNP marker				K_D, nM, MEAN ± SEM, *in silico*					Effect on human health during atherosclerosis, atherogenesis and atheroprotection [Ref]					
#	Entrez ID	dbSNP ID:min [36]	5′ flank, 10 bp	wt<br>min	3 flank, 10 bp	wt<br>min	Δ	Z	p	ϱ	Atherogenesis	ASg	Atheroprotection	ASp	Atherosclerosis	AS
8	*LCAT* (3931)	rs1486361482:G	acaccagata	A<br>G	ggacagccca	12.72±1.02<br>18.79±1.69	↓	6.71	10^{-6}	A	according to exhaustive review: low LCAT expression can often accelerate atherogenesis [152]	→	according to exhaustive review: age-related decrease in atheroprotective abilities may be associated with inactivation of HDL-associated enzymes, particularly LCAT [156]	→	a cohort-based clinical study [153]: radiotherapy decreases LCAT reactivity, which contributes to the atherosclerotic complications in breast cancer	→
		rs1188819587:G	ctcccacacc	A<br>G	gataaggaca	12.72±1.02<br>16.01±1.44	↓	3.88	10^{-3}	B						
		rs746342275:C	gtgctaccgc	A<br>C	agacagagga	44.85±3.59<br>55.88±4.47	↓	4.05	10^{-3}	B						
		rs749574144:G	tggatgtgct	A<br>G	ccgcaagaca	44.85±3.59<br>52.11±4.17	↓	2.69	10^{-2}	C						
		rs2058297744:G	gaccccttcc	A<br>G	cccgctgcag	140.23±12.62<br>185.54±14.84	↓	4.93	10^{-6}	A						
		rs1252783898:T	accagataag	G<br>T	acagcccagt	12.72±1.02<br>4.07±0.41	↑	17.72	10^{-6}	A	in human disease models using mice: a novel Lcat-activator DS-8190a prevents atherogenesis [155]	←	in human disease models using Lcat-knockout mice: enhanced inflammation, which were reversed by injection of adenovirus carrying *LCAT* gene as atheroprotection [151]	←	according to exhaustive review: recombinant human LCAT injections entered clinical phase II trials with good prospects for the treatment of atherosclerosis-related vascular diseases [154]	←
		rs1328187167:A	ttcaccatct	G<br>A	gctggatctc	44.85±3.59<br>19.95±1.80	↑	13.24	10^{-6}	A						
		rs751274627:T	aggacttctt	C<br>T	accatctggc	44.85±3.59<br>19.95±1.60	↑	14.11	10^{-6}	A						
		rs751274627:A	aggacttctt	C<br>A	accatctggc	44.85±3.59<br>13.37±1.07	↑	22.12	10^{-6}	A						
		rs780912694:T	taccgcaaga	C<br>T	agaggacttc	44.85±3.59<br>21.83±1.96	↑	12.27	10^{-6}	A						
		rs756625482:T	atgtgctacc	G<br>T	caagacagag	44.85±3.59<br>24.61±1.97	↑	10.78	10^{-6}	A						
		rs756625482:A	atgtgctacc	G<br>A	caagacagag	44.85±3.59<br>12.85±1.16	↑	21.24	10^{-6}	A						
		rs1055719644:T	gatgtgctac	C<br>T	gcaagacaga	44.85±3.59<br>22.95±1.84	↑	12.20	10^{-6}	A						
		rs1242606598:G	actggatgtg	C<br>G	taccgcaaga	44.85±3.59<br>38.22±3.82	↑	2.57	0.05	D						
		rs769067928:A	tgaactggat	G<br>A	tgctaccgca	44.85±3.59<br>20.97±1.68	↑	13.83	10^{-6}	A						
		rs760380606:C	agcctcggct	G<br>C	tctacacttg	14.63±1.46<br>11.86±1.19	↑	2.95	10^{-2}	C						
		rs2058307716:A	cacaagctgt	G<br>A	gtcagtcacag	35.99±2.16<br>17.87±1.25	↑	15.15	10^{-6}	A						
		rs2058307747:A	atcacaagct	G<br>A	tggtcagtca	35.99±2.16<br>26.66±1.60	↑	6.88	10^{-6}	A						

Table S3 (continued).

Gene		Candidate SNP marker				K_D, nM, MEAN ± SEM, *in silico*					Effect on human health during atherosclerosis, atherogenesis and atheroprotection [Ref]					
#	Entrez ID	dbSNP ID:min [36]	5′ flank, 10 bp	wt<br>min	3 flank, 10 bp	wt<br>min	Δ	Z	p	ϱ	Atherogenesis	ASg	Atheroprotection	ASp	Atherosclerosis	AS
8	*LCAT* (3931)	rs2058307786:T	gcatcacaag	C<br>T	tgtggtcagt	35.99±2.16<br>24.13±1.21	↑	9.97	10^{-6}	A	in human disease models using mice: a novel Lcat-activator DS-8190a prevents atherogenesis [155]	←	in human disease models using Lcat-knockout mice: enhanced inflammation, which were reversed by injection of adenovirus carrying *LCAT* gene as atheroprotection [151]	←	according to exhaustive review: recombinant human LCAT injections entered clinical phase II trials with good prospects for the treatment of atherosclerosis-related vascular diseases [154]	←
		rs115102600:A	ggcatcacaa	G<br>A	ctgtggtcag	35.99±2.16<br>16.50±1.16	↑	17.51	10^{-6}	A						
		rs2058297644:T	ccacccgctg	C<br>T	aggccagcag	140,23±12,62<br>55,33±4,98	↑	14.84	10^{-6}	A						
		rs764548177:A	ccttccaccc	G<br>A	ctgcaggcca	140,23±12,62<br>101,83±6,11	↑	6.08	10^{-6}	A						
		rs752061471:T	cccttccacc	C<br>T	gctgcaggcc	140,23±12,62<br>94,00±6,58	↑	7.39	10^{-6}	A						
		rs757555528:T	ccccttccac	C<br>T	cgctgcaggc	140,23±12,62<br>99,81±6,99	↑	6.16	10^{-6}	A						
		rs757555528:G	ccccttccac	C<br>G	cgctgcaggc	140,23±12,62<br>117,13±8,20	↑	3.30	10^{-3}	B						
		rs750602465:T	tgacccccttc	C<br>T	acccgctgca	140,23±12,62<br>26,93±2,69	↑	25.73	10^{-6}	A						
		rs750602465:G	tgacccccttc	C<br>G	acccgctgca	140,23±12,62<br>113,67±9,09	↑	3.75	10^{-3}	B						
		rs756397928:T	ctgaccccctt	C<br>T	cacccgctgc	140,23±12,62<br>53,16±3,72	↑	17.15	10^{-6}	A						
		rs756397928:G	ctgaccccctt	C<br>G	cacccgctgc	140,23±12,62<br>107,05±8,56	↑	4.66	10^{-3}	B						
		rs370589276:T	gccctgaccc	C<br>T	ttccacccgc	140,23±12,62<br>80,10±6,41	↑	9.50	10^{-6}	A						
		rs768783439:T	gctgccctga	C<br>T	cccttccacc	140,23±12,62<br>92,14±6,45	↑	7.48	10^{-6}	A						
		rs2058297840:T	accccagctg	C<br>T	cctgacccct	140.23±12.62<br>93.06±6.51	↑	7.41	10^{-6}	A						
		rs1288964186:T	tgccccagac	C<br>T	ccagctgccc	140.23±12.62<br>107.05±7.49	↑	5.03	10^{-6}	A						
		rs1378356884:T	gtgccccaga	C<br>T	cccagctgcc	140.23±12.62<br>102.85±7.20	↑	5.54	10^{-6}	A						
		rs113136822:T	gcaggtgccc	C<br>T	agaccccagc	140.23±12.62<br>58.75±5.88	↑	13.59	10^{-6}	A						
		rs2058297921:A	tggggcaggt	G<br>A	ccccagaccc	140.23±12.62<br>64.28±7.07	↑	11.33	10^{-6}	A						

Table S3 (continued).

Gene		Candidate SNP marker				K_D, nM, MEAN ± SEM, *in silico*					Effect on human health during atherosclerosis, atherogenesis and atheroprotection [Ref]					
#	Entrez ID	dbSNP ID:min [36]	5′ flank, 10 bp	wt<br>min	3 flank, 10 bp	wt<br>min	Δ	Z	p	ϱ	Atherogenesis	ASg	Atheroprotection	ASp	Atherosclerosis	AS
9	*NFE2L2* (4780)	rs1439704525:C	ccccgccctt	G<br>C	tggggcggga	52.63±4.21<br>86.77±6.07	↓	9.33	10^{-6}	A	in human disease models using Nrf2-deficient A/J mice: higher oxidative stress [161] as a risk-factor for an accelerated atherogenesis [162]	→	in human disease models with human coronary arterial endothelial cells with: impaired angiogenesis and ability to form capillary-like structures [157]	→	according to an exhaustive review: NRF2 deficiency in macrophages increases atherosclerosis [159]	→
		rs898346091:G	gccccgccct	T<br>G	gtggggcggg	52.63±4.21<br>161.30±12.90	↓	19.83	10^{-6}	A						
		rs1690854426:A	agtttgagcg	G<br>A	cccggtgggc	21.83±2.40<br>35.28±3.18	↓	6.86	10^{-6}	A						
		rs1558995250:A	aagtttgagc	G<br>A	gcccggtggg	21.83±2.40<br>18.42±2.39	↓	2.00	0.05	D						
		rs1690854559:C	ccctaagttt	G<br>C	agcggcccgg	21.83±2.40<br>40.18±3.62	↓	8.48	10^{-6}	A						
		rs1690854611:G	cccctaagtt	T<br>G	gagcggcccgg	21.83±2.40<br>58.16±4.07	↓	15.00	10^{-6}	A						
		rs1211937585:A	ccggagcccc	T<br>A	aagtttgagc	21.83±2.40<br>36.35±2.91	↓	7.58	10^{-6}	A						
		rs1158202397:C	agcgccttaa	G<br>C	tgcccagcgg	12.22±1.34<br>16.01±2.08	↓	3.17	10^{-2}	C						
		rs1690837301:G	ggagcgcctt	A<br>G	agtgcccagc	12.22±1.34<br>39.78±3.58	↓	16.20	10^{-6}	A						
		rs1690821772:G	cctgctttat	A<br>G	gcgtgcaaac	3.72±0.37<br>10.95±1.09	↓	15.08	10^{-6}	A						
		rs1283971818:G	ttcctgcttt	A<br>G	tagcgtgcaa	3.72±0.37<br>10.11±0.91	↓	14.17	10^{-6}	A						
		rs1690821979:C	tttcctgctt	T<br>C	atagcgtgca	3.72±0.37<br>11.28±1.02	↓	15.76	10^{-6}	A						

Table S3 (continued).

Gene		Candidate SNP marker				K_D, nM, MEAN ± SEM, *in silico*					Effect on human health during atherosclerosis, atherogenesis and atheroprotection [Ref]					
#	Entrez ID	dbSNP ID:min [36]	5′ flank, 10 bp	wt<br>min	3 flank, 10 bp	wt<br>min	Δ	Z	p	ϱ	Atherogenesis	ASg	Atheroprotection	ASp	Atherosclerosis	AS
		rs1690878751:T	ccttgtgggg	C<br>T	gggaggcgga	52.63±4.21<br>46.68±3.73	↑	2.25	0.05	D						
		rs1690878906:T	ttgccccgcc	C<br>T	ttgtggggcg	52.63±4.21<br>26.13±2.09	↑	12.90	10^{-6}	A						
		rs1690879080:T	cttgccccgc	C<br>T	gggaggcgga	52.63±4.21<br>30.06±2.40	↑	10.38	10^{-6}	A						
		rs1158202397:A	agcgccttaa	G<br>A	tgcccagcgg	12.22±1.34<br>10.01±1.30	↑	2.29	0.05	D						
		rs1690837505:A	gggaggagcg	C<br>A	cttaagtgcc	12.22±1.34<br>9.52±1.05	↑	3.07	10^{-2}	C						
		rs1485587982:C	ctgctttata	G<br>C	cgtgcaaacc	3.72±0.37<br>3.11±0.31	↑	2.50	0.05	D						
		rs1219147859:A	gccctttcct	G<br>A	ctttatagcg	3.72±0.37<br>2.52±0.25	↑	5.25	10^{-6}	A						
		rs993609402:A	gccctttcct	G<br>A	cagccctcgg	60.54±5.45<br>33.22±2.66	↑	9.71	10^{-6}	A						
9	*NFE2L2* (4780)	rs993609402:C	ggccgtcaca	G<br>C	cagccctcgg	60.54±5.45<br>51.59±4.64	↑	2.41	0.05	D	in human smoking-induced atherosclerosis models using rats: Nrf2 upregulation improves endothelial cell viability treated with cigarette smoke extract [163]	←	in human disease models using the human immortalized keratinocyte cells (HaCat): improved wound healing due to alleviated oxidative stress, and decreased apoptosis [158]	←	in human disease models using the atherosclerotic mice: alleviated atherosclerosis due to enhanced autophagy and inhibited ferroptosis [160]	←
		rs993609402:T	ggccgtcaca	G<br>T	cagccctcgg	60.54±5.45<br>35.99±2.88	↑	8.73	10^{-6}	A						
		rs1690823251:T	ctcggttggc	C<br>T	gtcacagcag	60.54±5.45<br>40.18±3.62	↑	6.38	10^{-6}	A						
		rs1690824262:C	ccttggcttc	C<br>T	gtgacagcgc	60.54±5.45<br>31.29±2.50	↑	10.90	10^{-6}	A						
		rs1244557625:T	cgcccggcgc	G<br>T	accattcgct	61.76±3.71<br>42.66±4.27	↑	6.48	10^{-6}	A						
		rs911016557:T	cactcagagc	G<br>T	gcgcccggcg	61.76±3.71<br>50.57±4.05	↑	4.18	10^{-3}	B						
		rs1395569529:A	gggcgcgggt	G<br>A	aaaggtcact	61.76±3.71<br>18.05±1.62	↑	23.56	10^{-6}	A						
		rs1342618184:C	gctgggcgcg	G<br>C	gtgaaaggtc	61.76±3.71<br>54.78±3.83	↑	2.41	0.05	D						
		rs1685050986:A	cgctgggcgc	G<br>A	ggtgaaaggt	61.76±3.71<br>53.16±3.72	↑	3.34	10^{-3}	B						

Table S3 (continued).

#	Entrez ID	dbSNP ID:min [36]	5′ flank, 10 bp	wt min	3 flank, 10 bp	K_D, nM, MEAN ± SEM, *in silico* wt min	Δ	Z	p	ϱ	Atherogenesis	ASg	Atheroprotection	ASp	Atherosclerosis	AS
Gene		Candidate SNP marker									Effect on human health in atherosclerosis, atherogenesis and atheroprotection [Ref]					
		rs967734058:G	ccatcttact	T G	agggacctgc	13.78±1.24 15.69±1.41	↓	2.11	0.05	D	according to exhaustive review [165]: Nr1h3-knockout mice demonstrated accelerated atherosclerosis	→	in human disease models using Nr1h3-deficient mice: reduced atheroprotection [168]	→	in human disease models using Nr1h3-knockout mice: more aortic atherosclerotic lesions [166]	→
		rs1042479145:G	ccaaattgct	A G	cttctctggg	22.05±1.76 43.09±2.59	↓	12.72	10^{-6}	A						
		rs571195097:C	ccaggcagga	A C	ggaggggtgg	79.30±5.55 94.00±6.58	↓	3.59	10^{-3}	B						
		rs1955313655:C	tgcctatgga	G C	gggagggaac	29.47±2.65 35.99±3.60	↓	3.00	10^{-2}	C						
		rs1955313214:G	cgaggtgcct	A G	tggaggggag	29.47±2.65 45.30±2.72	↓	7.88	10^{-6}	A						
		rs1955365617:T	attgctactt	C T	tctggggctc	22.05±1.76 19.36±1.74	↑	2.21	0.05	D	according to exhaustive review [167]: NR1H3-agonists suppressed immunity that can slow down atherogenesis	←	a cohort-based clinical study [164]: NR1H1 level increased as a response to the atherogenesis progress as atheroprotection	←	according to exhaustive review [167]: NR1H3-agonists have anti-inflammatory effects that can alleviate atherosclerosis	←
		rs1955365617:G	attgctactt	C G	tctggggctc	22.05±1.76 14.06±0.98	↑	8.03	10^{-6}	A						
		rs913279941:A	tccccaaatt	G A	ctacttctct	22.05±1.76 4.41±0.49	↑	23.36	10^{-6}	A						
		rs1347016106:G	cctttcccca	A G	attgctactt	22.05±1.76 18.05±1.62	↑	3.30	10^{-3}	B						
10	*NR1H3* (10062)	rs551015896:T	gggacctttg	C T	tccacgaggt	29.47±2.65 26.13±1.83	↑	1.98	0.05	D						
		rs1955311455:A	tgggaccttt	G A	ctccacgagg	29.47±2.65 14.78±1.48	↑	10.06	10^{-6}	A						
		rs1470984249:T	cgccatctta	C T	ttagggacct	8.44±1.18 6.07±0.73	↑	3.54	10^{-3}	B						
		rs1272788302:T	aggaaggagg	G T	gtggcctgac	79.30±5.55 66.90±5.35	↑	3.12	10^{-2}	C						
		rs1247126883:A	ggagggctgt	G A	gtcaccaggc	79.30±5.55 35.63±2.49	↑	15.81	10^{-6}	A						
		rs980360050:A	gaggggaggg	C A	tgtggtcacc	79.30±5.55 54.78±3.83	↑	7.28	10^{-6}	A						
		rs146975164:T	gctcagtgtc	G T	caattccggg	80.90±6.47 70.33±5.63	↑	2.36	0.05	D						
		rs1955304541:A	agggaggctg	G A	gaaagccgct	80.90±6.47 64.93±4.55	↑	3.90	10^{-3}	B						
		rs138153600:A	gagggaacac	G A	attctggagg	29.47±2.65 25.87±1.81	↑	2.30	0.05	D						
		rs752776074:T	gaggggaggg	A T	acacgattct	29.47±2.65 19.17±1.92	↑	6.37	10^{-6}	A						

Table S3 (continued).

Gene		Candidate SNP marker				K_D, nM, MEAN ± SEM, *in silico*					Effect on human health during atherosclerosis, atherogenesis and atheroprotection [Ref]					
#	Entrez ID	dbSNP ID:min [36]	5′ flank, 10 bp	wt<br>min	3 flank, 10 bp	wt<br>min	Δ	Z	p	ϱ	Atherogenesis	ASg	Atheroprotection	ASp	Atherosclerosis	AS
11	*PF4* (5196)	rs956857054:A	ggacagccgg	G<br>A	aataaaacgt	7.12±0.64<br>8.03±0.72	↑	1.99	0.05	D	according to exhaustive review: pro-atherogenic effects [169]	→	according to exhaustive review: anti-angiogenic effects, which can reduce the formation of atherosclerotic plaques [169]	←	in human disease models using mice injected with recombinant human PF4: induced inflammation [170], which can aggravate atherosclerosis [191]	→
		rs1186687803:A	aggacagccg	G<br>A	gaataaaacg	7.12±0.64<br>6.07±0.55	↑	2.57	0.05	D						
		rs1718819711:A	aaggacagcc	G<br>A	ggaataaaac	7.12±0.64<br>5.66±0.51	↑	3.65	10^{-3}	B						
12	*PON1* (5444)	rs1807973665:T	cagggctgcg	G<br>T	ctgcaggcag	231.20±18.50<br>99.81±7.98	↑	15.19	10^{-6}	A	according to an exhaustive review: transgenic mice with additional human PON1 gene as a human disease model had an excess of foam cells as a risk factor for atherogenesis [173]	→	in human disease models using transgenic mice carrying human PON1 gene: improved efflux, reverse transport and homeostasis of cholesterol as atheroprotection [175]	←	according to an exhaustive review: an increase in PON1 activity would be expected to decrease inflammation and atherosclerosis [174]	←
		rs950406818:A	gcagggctgc	G<br>A	gctgcaggca	231.20±18.50<br>185.54±16.70	↑	3.48	10^{-3}	B						
		rs1180660384:A	agcagggctg	C<br>A	ggctgcaggc	231.20±18.50<br>207.11±14.50	↑	2.05	0.05	D						
		rs1807974016:A	tgccccagca	G<br>A	ggctgcggct	231.20±18.50<br>119.49±8.36	↑	12.29	10^{-6}	A						
		rs1237732763:T	gctgccccag	C<br>T	agggctgcgg	231.20±18.50<br>48.10±4.33	↑	25.88	10^{-6}	A						
		rs1807974238:T	ggcgctgccc	C<br>T	agcagggctg	231.20±18.50<br>74.68±6.72	↑	18.89	10^{-6}	A						
		rs1807974314:A	aatcggcgct	G<br>A	ccccagcagg	231.20±18.50<br>102.85±10.29	↑	12.83	10^{-6}	A						
		rs1204551656:T	ccaatcggcg	C<br>T	tgccccagca	231.20±18.50<br>154.98±10.85	↑	7.34	10^{-6}	A						
		rs1354114432:T	cgggccaatc	G<br>T	gcgctgcccc	231.20±18.50<br>66.90±5.35	↑	22.51	10^{-6}	A						
		rs1347833734:T	gcgggccaat	C<br>T	ggcgctgccc	231.20±18.50<br>129.45±10.36	↑	10.14	10^{-6}	A						
		rs1006420794:T	cgacccggcg	G<br>T	ggaggggcgg	231.20±18.50<br>191.19±15.30	↑	3.33	10^{-3}	B						
13	*PON2* (5445)	rs957961086:C	gcctaggcgg	A<br>C	ggacggggcg	167.88±15.11<br>193.11±19.31	↓	2.03	0.05	D	in human disease models using Pon2-knockout mice: accelerated atherogenesis [178]	→	in human disease models using human endothelial EAhy926 cells under RNAi-mediated Pon2-knockdown: vast reactive oxygen species formation [179] as atheroprotection [180, 206]	←	in human disease models using Pon2-knockout mice: vascular inflammation, blood coagulation abnormalities, increased oxidative stress and endothelial dysfunction that worsen atherosclerosis [178]	→
		rs1789543296:C	gtgggttgtt	T<br>C	gaaaaaggaa	13.64±0.82<br>28.60±2.00	↓	16.01	10^{-6}	A						
		rs1789543351:C	ggtgggttgt	T<br>C	tgaaaaagga	13.64±0.82<br>24.13±1.69	↓	12.47	10^{-6}	A						
		rs1007928363:G	gcggccggca	C<br>G	catcgagccg	96.86±6.78<br>109.21±7.64	↓	2.52	0.05	D						
		rs1019283624:G	cgcggccggc	A<br>G	ccatcgagcc	96.86±6.78<br>109.21±7.64	↓	2.52	0.05	D						

Table S3 (continued).

Gene		Candidate SNP marker				K_D, nM, MEAN ± SEM, *in silico*					Effect on human health during atherosclerosis, atherogenesis and atheroprotection [Ref]					
#	Entrez ID	dbSNP ID:min [36]	5′ flank, 10 bp	wt<br>min	3 flank, 10 bp	wt<br>min	Δ	Z	p	ϱ	Atherogenesis	ASg	Atheroprotection	ASp	Atherosclerosis	AS
13	*PON2* (5445)	rs925124276:T	ggcggaggac	G<br>T	gggcggagcg	167.88±15.11<br>112.54±9.00	↑	6.44	10^{-6}	A	in human disease models using mouse macrophages J774A.1 cell line: pomegranate juice slows atherogenesis due to stimulating an additional PON2 expression in macrophage [182]	←	according to an exhaustive review: PON2 expression level appear to be higher in female mice compared with male ones that leads an increased atheroprotection in female mice [181]	←	according to an exhaustive review: PON2 overexpression decreases atherosclerotic lesions [181]	←
		rs925124276:A	ggcggaggac	G<br>A	gggcggagcg	167.88±15.11<br>118.30±8.28	↑	5.87	10^{-6}	A						
		rs1789536504:T	aggcggagga	C<br>T	ggggcggagc	167.88±15.11<br>125.62±8.79	↑	4.89	10^{-3}	B						
		rs1312040763:A	ctaggcggag	G<br>A	acggggcgga	167.88±15.11<br>75.43±6.03	↑	13.13	10^{-6}	A						
		rs1789536654:A	cggcctaggc	G<br>A	gaggacgggg	167.88±15.11<br>85.91±7.73	↑	10.26	10^{-6}	A						
		rs1237334719:T	tcggcctagg	C<br>T	ggaggacggg	167.88±15.11<br>43.96±4.40	↑	19.30	10^{-6}	A						
		rs530897319:A	actcggccta	G<br>A	gcggaggacg	167.88±15.11<br>68.26±7.51	↑	12.46	10^{-6}	A						
		rs1789536834:G	gggactcggc	C<br>G	taggcggagg	167.88±15.11<br>99.81±9.98	↑	7.62	10^{-6}	A						
		rs1209566662:T	agggactcgg	C<br>T	ctaggcggag	167.88±15.11<br>55.33±4.98	↑	16.76	10^{-6}	A						
		rs991174688:A	cagggactcg	G<br>A	cctaggcgga	167.88±15.11<br>48.58±4.86	↑	18.42	10^{-6}	A						
		rs550708283:A	gcagggactc	G<br>A	gcctaggcgg	167.88±15.11<br>72.48±5.80	↑	13.59	10^{-6}	A						
		rs1200671015:T	cgcagggact	C<br>T	ggcctaggcg	167.88±15.11<br>95.90±7.67	↑	9.09	10^{-6}	A						
		rs1167262700:T	gacgcaggga	C<br>T	tcggcctagg	167.88±15.11<br>128.16±11.53	↑	4.12	10^{-3}	B						
		rs1789537162:T	ccgacgcagg	G<br>T	actcggccta	167.88±15.11<br>42.24±5.07	↑	18.38	10^{-6}	A						
		rs1789537162:A	ccgacgcagg	G<br>A	actcggccta	167.88±15.11<br>103.88±7.27	↑	8.09	10^{-6}	A						
		rs1422463347:A	ccccgacgca	G<br>A	ggactcggcc	167.88±15.11<br>132.06±11.89	↑	3.74	10^{-3}	B						
		rs748149426:A	cggccccgac	G<br>A	cagggactcg	167.88±15.11<br>64.93±5.19	↑	15.15	10^{-6}	A						
		rs1789537561:T	gcggccccga	C<br>T	gcagggactc	167.88±15.11<br>134.73±10.78	↑	3.46	10^{-3}	B						

Table S3 (continued).

Gene		Candidate SNP marker				K_D, nM, MEAN ± SEM, *in silico*					Effect on human health during atherosclerosis, atherogenesis and atheroprotection [Ref]					
#	Entrez ID	dbSNP ID:min [36]	5′ flank, 10 bp	wt<br>min	3 flank, 10 bp	wt<br>min	Δ	Z	p	ϱ	Atherogenesis	ASg	Atheroprotection	ASp	Atherosclerosis	AS
13	*PON2* (5445)	rs949406810:T	tccgcggccc	C<br>T	gacgcaggga	167.88±15.11<br>125.62±10.05	↑	4.66	10^{-3}	B	in human disease models using mouse macrophages J774A.1 cell line: pomegranate juice slows atherogenesis due to stimulating an additional PON2 expression in macrophage [182]	←	according to an exhaustive review: PON2 expression level appear to be higher in female mice compared with male ones that leads an increased atheroprotection in female mice [181]	←	according to an exhaustive review: PON2 overexpression decreases atherosclerotic lesions [181]	←
		rs949406810:A	tccgcggccc	C<br>A	gacgcaggga	167.88±15.11<br>125.62±10.05	↑	4.59	10^{-3}	B						
		rs1017157543:A	ggtttcgggt	G<br>A	ggttgtttga	13.64±0.82<br>11.98±0.84	↑	2.73	10^{-2}	C						
		rs1203809932:T	gggaagatgg	C<br>T	accgcccacg	96.86±6.78<br>58.16±4.65	↑	9.69	10^{-6}	A						
		rs1171473483:G	tcgagccggg	A<br>G	agatggcacc	96.86±6.78<br>85.91±6.87	↑	2.20	0.05	D						
		rs1165785791:A	ccggcaccat	C<br>A	gagccgggaa	96.86±6.78<br>19.36±1.74	↑	27.53	10^{-6}	A						
		rs1165785791:G	ccggcaccat	C<br>G	gagccgggaa	96.86±6.78<br>82.54±6.60	↑	3.02	10^{-2}	C						
14	*SERPINF1* (5176)	rs1907124305:T	ggattagaag	G<br>T	cagcaaaaaa	14.49±1.16<br>17.00±1.19	↓	3.14	10^{-2}	C	according to exhaustive review [187]: SERPINF1 deficiency can lead to increased microvessel density and more thicker walled blood vessels that can slow atherogenesis down	←	a cohort-based clinical study: SERPINF1 deficiency is a biomarker for angiogenesis [186], which contributes atheroprotection	←	in human disease models using the SERPINF1-deficient mice: aggravated atherosclerosis [188]	→
		rs929591238:C	ctggattaga	A<br>C	ggcagcaaaa	14.49±1.16<br>17.00±1.36	↓	2.92	10^{-2}	C						
		rs1907123941:C	ggacgctgga	T<br>C	tagaaggcag	14.49±1.16<br>19.95±2.00	↓	5.28	10^{-6}	A						
		rs1351227997:C	tgaccactat	T<br>C	taggccggca	3.95±0.40<br>4.87±0.54	↓	2.70	10^{-2}	C						
		rs1461693685:C	caatgaccac	T<br>C	atttaggccg	3.95±0.40<br>4.87±0.54	↓	2.70	10^{-2}	C						
		rs1330089421:G	aacaatgacc	A<br>G	ctatttaggc	3.95±0.40<br>4.87±0.54	↓	2.70	10^{-2}	C						
		rs1274932688:G	gtgcaggctt	A<br>G	gagggactag	36.35±2.91<br>43.52±3.92	↓	2.99	10^{-2}	C						
		rs1191508994:G	tctggatctt	T<br>G	aaaagctatt	7.64±0.53<br>30.98±1.86	↓	28.83	10^{-6}	A						

Table S3 (continued).

Gene		Candidate SNP marker				K_D, nM, MEAN ± SEM, *in silico*					Effect on human health during atherosclerosis, atherogenesis and atheroprotection [Ref]					
#	Entrez ID	dbSNP ID:min [36]	5′ flank, 10 bp	wt<br>min	3 flank, 10 bp	wt<br>min	Δ	Z	p	ϱ	Atherogenesis	ASg	Atheroprotection	ASp	Atherosclerosis	AS
14	*SERPINF1* (5176)	rs1907124539:T	tagaaggcag	C<br>T	aaaaaaagct	14.49±1.16<br>6.38±0.57	↑	13.94	10^6	A	in human atherogenesis models using aged vascular smooth muscle cells: SERPINF1 excess is a biomarker for atherosclerotic plaque [185]	→	according to exhaustive review [183]: blocked regeneration in human wound-healing models using mice [184]	→	in polycystic ovary syndrome: controlled inflammation [189] via inhibition [190], when atherosclerosis is a chronic inflammation [191]	←
		rs1597343431:T	tggacgctgg	A<br>T	ttagaaggca	14.49±1.16<br>9.91±0.89	↑	6.11	10^6	A						
		rs1182927022:A	caggtgtata	G<br>A	ggcatggggg	3.95±0.40<br>3.30±0.36	↑	2.62	10^2	C						
		rs144422572:A	gccggcaggt	G<br>A	tatagggcat	3.95±0.40<br>1.45±0.17	↑	12.76	10^6	A						
		rs1162211182:A	ccactattta	G<br>A	gccggcaggt	3.95±0.40<br>2.67±0.27	↑	5.67	10^6	A						
		rs900617887:A	taggctgggt	G<br>A	tggagctgca	36.35±2.91<br>30.06±3.01	↑	2.90	10^2	C						
		rs1039127862:T	agggactagg	C<br>T	tgggtgtgga	36.35±2.91<br>30.06±2.40	↑	3.25	10^2	C						
		rs1907127910:A	agagggacta	G<br>A	gctgggtgtg	36.35±2.91<br>19.36±1.74	↑	10.18	10^6	A						
		rs1907127533:A	aggcttagag	G<br>A	gactaggctg	36.35±2.91<br>17.52±1.58	↑	12.16	10^6	A						
		rs944665448:T	agtgtgcagg	C<br>T	ttagagggac	36.35±2.91<br>16.50±1.48	↑	12.53	10^6	A						
		rs1235015122:C	tcagtgtgca	G<br>C	gcttagaggg	36.35±2.91<br>28.60±2.29	↑	4.15	10^3	B						
		rs1275065786:A	agtgcaggtc	G<br>A	ctttaagaaa	10.21±0.92<br>6.64±0.53	↑	7.29	10^6	A						
		rs1907744573:A	gaaatctgct	G<br>A	cccccctggc	58.75±4.70<br>36.35±3.27	↑	8.40	10^6	A						
		rs1484347269:A	cgcgatgtgg	G<br>A	gaaatctgct	58.75±4.70<br>35.99±2.16	↑	9.83	10^6	A						
		rs189044234:A	gcagtggcgc	G<br>A	atgtggggaa	58.75±4.70<br>51.07±4.09	↑	2.61	10^2	C						
		rs1463623596:T	ggcagtggcg	C<br>T	gatgtgggga	58.75±4.70<br>37.46±3.00	↑	8.15	10^6	A						
		rs572878888:A	gggcagtggc	G<br>A	cgatgtgggg	58.75±4.70<br>37.09±2.60	↑	8.81	10^6	A						
		rs945933556:A	agggcagtgg	C<br>A	gcgatgtggg	58.75±4.70<br>51.07±4.09	↑	2.62	10^2	C						
		rs1413285931:G	tttaaaagct	A<br>G	ttggttcagg	7.64±0.53<br>6.64±0.60	↑	2.42	0.05	D						
		rs541151948:A	gagtgcaggt	C<br>A	gctttaagaa	10.21±0.92<br>7.27±0.51	↑	5.94	10^6	A	in the ClinVar database: recessive osteogenesis imperfecta [43]; in a patient with this disease [192], vessels were narrowed by hypertrophied endothelial cells, as in atherogenesis [185]	→	in the ClinVar database: recessive osteogenesis imperfecta [43]; according to a retrospective clinical cohort-based study [193], delayed fracture healing in this disease like delayed vascular wound healing in the case of reduced atheroprotection [183, 184]	→	in the ClinVar database: recessive osteogenesis imperfecta [43]; according to a clinical cohort study of children with this disease [194], continuous controlled mild inflammation, as in atherosclerosis [189-191]	←
		rs541151948:T	gagtgcaggt	C<br>T	gctttaagaa	10.21±0.92<br>8.53±0.77	↑	2.97	10^2	C						

Table S3 (continued).

	Gene	Candidate SNP marker				K_D, nM, MEAN ± SEM, *in silico*					Effect on human health during atherosclerosis, atherogenesis and atheroprotection [Ref]					
#	Entrez ID	dbSNP ID:min [36]	5′ flank, 10 bp	wt<br>min	3 flank, 10 bp	wt<br>min	Δ	Z	p	ϱ	Atherogenesis	ASg	Atheroprotection	ASp	Atherosclerosis	AS
		rs1189955365:C	aataaataca	T<br>C	ttctgttctt	2.52±0.18<br>2.92±0.26	↓	2.85	10^{-2}	C						
		rs1447693055:C	agagctataa	T<br>C	aaatacattt	2.52±0.18<br>2.92±0.26	↓	2.85	10^{-2}	C						
		rs1447693055:G	agagctataa	T<br>G	aaatacattt	2.52±0.18<br>2.92±0.26	↓	2.85	10^{-2}	C						
		rs1169024514:C	cagagctata	A<br>C	taaatacatt	2.52±0.18<br>2.92±0.26	↓	2.85	10^{-2}	C						
		rs1736880996:G	ctccagagct	A<br>G	taataaatac	2.52±0.18<br>2.92±0.26	↓	2.85	10^{-2}	C						
		rs1021057878:C	cctccagagc	T<br>C	ataataaata	2.52±0.18<br>2.92±0.26	↓	2.85	10^{-2}	C						
		rs988046151:T	gcctccagag	C<br>T	tataataaat	2.52±0.18<br>2.92±0.26	↓	2.85	10^{-2}	C						
		rs1736879696:G	cccagcctcc	A<br>G	gagctataat	2.52±0.18<br>2.81±0.17	↓	2.44	0.05	D						
15	*TLR2* (7097)	rs1578950658:C	cccgcacccc	A<br>C	gaaggagggg	62.38±4.99<br>104.93±8.39	↓	9.91	10^{-6}	A	in a human disease model using Tlr2-knockout mice: too few foam cells, the formation of which is a key event in early atherogenesis [199]	←	in a human atherogenesis model using Tlr2-knockout mice: a reduced accumulation of lipids, foam cells, and endothelial cell injuries [197]	←	in human disease models using Tlr2-deficient mice: heightened inflammation [196]	→
		rs1735505267:C	gcttacttcc	T<br>C	agtcccgggc	20.97±1.89<br>27.75±2.22	↓	4.85	10^{-3}	B						
		rs1471609683:G	ccccttttgct	T<br>G	acttcctagt	20.97±1.89<br>27.20±2.72	↓	4.01	10^{-3}	B						
		rs1194710459:C	tcccttttgc	T<br>C	tacttcctag	20.97±1.89<br>26.66±1.87	↓	4.25	10^{-3}	B						
		rs757468634:C	cttttatttg	T<br>C	aggttgaagc	4.73±0.43<br>7.34±0.59	↓	7.33	10^{-6}	A						
		rs1737007146:T	tcttttattt	G<br>T	taggttgaag	4.73±0.43<br>6.32±0.51	↓	4.91	10^{-6}	A						
		rs1194710459:C	tcccttttgc	T<br>C	tacttcctag	20.97±1.89<br>26.66±1.87	↓	4.25	10^{-3}	B						
		rs1578950658:C	cccgcacccc	A<br>C	gaaggagggg	62.38±4.99<br>104.93±8.39	↓	9.91	10^{-6}	A						
		rs1396720838:G	ccagccttgc	A<br>G	cggggcagct	88.52±7.08<br>102.85±9.26	↓	2.41	0.05	D						
		rs1735488975:C	gacccagcct	T<br>C	gcacggggca	88.52±7.08<br>102.85±9.26	↓	2.42	0.05	D						

Table S3 (continued).

Gene		Candidate SNP marker				K_D, nM, MEAN ± SEM, *in silico*					Effect on human health during atherosclerosis, atherogenesis and atheroprotection [Ref]					
#	Entrez ID	dbSNP ID:min [36]	5′ flank, 10 bp	wt<br>min	3 flank, 10 bp	wt<br>min	Δ	Z	p	ϱ	Atherogenesis	ASg	Atheroprotection	ASp	Atherosclerosis	AS
15	*TLR2* (7097)	rs1736881700:G	tataataaat	A<br>G	catttctgtt	2.52±0.18<br>2.19±0.22	↑	2.27	0.05	D	in human atherosclerosis models using macrophage cell lines: exogenous TLR2 reduces as a potential therapeutic preventer of atherogenesis [195]	←	in a human disease model using microvascular endothelial cells: TLR2 excess promotes angiogenesis and cell adhesion [198]	←	according to a microarray data meta-analysis: TLR2 excess may have a crucial role in progressing from coronary atherosclerosis to myocardial infarction [200]	→
		rs1365451972:T	gcggactttc	C<br>T	cttttgctta	62.38±4.99<br>34.24±2.40	↑	11.46	10^{-6}	A						
		rs1470210555:T	cgcggacttt	C<br>T	ccttttgctt	62.38±4.99<br>42.66±2.99	↑	7.32	10^{-6}	A						
		rs1393402068:T	accccagaag	G<br>T	agggggcgcc	62.38±4.99<br>18.60±1.49	↑	21.92	10^{-6}	A						
		rs1735501502:C	ccgcacccca	G<br>C	aaggaggggg	62.38±4.99<br>37.84±3.03	↑	9.17	10^{-6}	A						
		rs987615193:G	ttcccgcacc	C<br>G	cagaaggagg	62.38±4.99<br>49.56±3.47	↑	4.36	10^{-3}	B						
		rs1735501306:A	ggttcccgca	C<br>A	cccagaagga	62.38±4.99<br>49.56±3.47	↑	4.45	10^{-3}	B						
		rs929450367:A	ggcggttccc	G<br>A	caccccagaa	62.38±4.99<br>50.57±3.54	↑	4.07	10^{-3}	B						
		rs927217196:A	tttgcttact	T<br>A	cctagtcccg	20.97±1.89<br>5.60±0.62	↑	18.70	10^{-6}	A						
		rs1735505029:T	cttttgctta	C<br>T	ttcctagtcc	20.97±1.89<br>13.24±1.19	↑	7.35	10^{-6}	A						
		rs1735504577:C	gactttccct	T<br>C	ttgcttactt	20.97±1.89<br>17.69±1.59	↑	2.64	10^{-2}	C						
		rs1735504441:C	ggactttccc	T<br>C	tttgcttact	20.97±1.89<br>17.00±1.53	↑	3.28	10^{-2}	C						
		rs1560731404:T	cggactttcc	C<br>T	ttttgcttac	20.97±1.89<br>18.05±1.26	↑	2.55	0.05	D						
		rs914509126:A	tttgtaggtt	G<br>A	aagcactgga	4.73±0.43<br>3.36±0.30	↑	5.31	10^{-6}	A						
		rs754074952:C	tgaacctctt	T<br>C	tatttgtagg	4.73±0.43<br>3.95±0.36	↑	2.85	10^{-2}	C						
		rs757238850:A	cttatttgaa	C<br>A	ctcttttatt	4.73±0.43<br>4.15±0.33	↑	2.21	0.05	D						
		rs1326655755:A	aggggcggtt	C<br>A	ccgcacccca	62.38±4.99<br>52.63±7.37	↑	2.03	0.05	D						
		rs1735493155:A	ccgcctctgg	C<br>A	ctgtcgcagc	88.52±7.08<br>65.58±5.25	↑	5.26	10^{-6}	A						

Table S3 (continued).

Gene		Candidate SNP marker				K_D, nM, MEAN ± SEM, *in silico*					Effect on human health during atherosclerosis, atherogenesis and atheroprotection [Ref]					
#	Entrez ID	dbSNP ID:min [36]	5′ flank, 10 bp	wt min	3 flank, 10 bp	wt min	Δ	Z	p	ϱ	Atherogenesis	ASg	Atheroprotection	ASp	Atherosclerosis	AS
15	*TLR2* (7097)	rs1044715191:A	ggacccgcct	C A	tggcctgtcg	88.52±7.08 65.58±6.56	↑	4.72	10^{-3}	B	in human atherosclerosis models using macrophage cell lines: exogenous TLR2 reduces as a potential therapeutic preventer of atherogenesis [195]	←	in a human disease model using microvascular endothelial cells: TLR2 excess promotes angiogenesis and cell adhesion [198]	←	according to a microarray data meta-analysis: TLR2 excess may have a crucial role in progressing from coronary atherosclerosis to myocardial infarction [200]	→
		rs1735491448:T	ggcagctgtc	G T	gggcaggacc	88.52±7.08 75.43±5.28	↑	2.94	10^{-2}	C						
		rs1404758587:A	cacggggcag	C A	tgtcggggca	88.52±7.08 41.82±3.35	↑	13.26	10^{-6}	A						
		rs1363622662:A	gcacggggca	G A	ctgtcggggc	88.52±7.08 59.34±5.34	↑	6.82	10^{-6}	A						
		rs1735490253:T	cagccttgca	C T	ggggcagctg	88.52±7.08 55.33±4.43	↑	8.46	10^{-6}	A						
		rs923383304:T	tcgacccagc	C T	ttgcacgggg	88.52±7.08 50.57±4.05	↑	9.92	10^{-6}	A						
16	YAP1 (10413)	rs951717412:G	ctcctaactt	T G	agttttggcg	15.23±1.07 21.18±1.69	↓	6.34	10^{-6}	A	in human disease models using human umbilical vein endothelial cells (HUVECs): methotrexate exerts atheroprotective effects due to YAP1 downregulation [203]	←	in human disease models using Yap1-deficient mice: loss of atheroprotective alignment of vascular endothelial cells in the direction of blood flow [204]	→	in human disease models using atherosclerotic mice with endothelial-cell-specific Yap1-excess: harmin (a natural extract from *Peganum harmala*) reduces Yap1 activity that relieves atherosclerosis [207]	←
		rs1375834176:C	caaaagttaa	T C	aggttttcca	8.28±0.83 18.05±1.62	↓	11.78	10^{-6}	A						
		rs1943014839:G	ccaaaagtta	A G	taggttttcc	8.28±0.83 12.35±1.11	↓	5.85	10^{-6}	A						
		rs1943014574:G	cccaaaagtt	A G	ataggttttc	8.28±0.83 22.95±1.84	↓	16.01	10^{-6}	A						
		rs207472274:G	aatttactat	A G	taaaccataa	9.05±0.81 10.52±0.95	↓	2.34	0.05	D						
		rs1947929389:G	ggagaaattt	A G	ctatataaac	9.05±0.81 41.40±2.90	↓	26.56	10^{-6}	A						
		rs1315435628:G	gtccgggcgt	A G	gccctcgctc	145.95±13.14 181.87±14.55	↓	3.62	10^{-3}	B						
		rs983135768:G	aactttagtt	T G	tggcgtttga	15.23±1.07 11.51±0.81	↑	5.77	10^{-6}	A	according to an exhaustive review: inhibited apoptosis [208] that can retard atherogenesis [209]	←	in human disease models using human colorectal cancer SW48 cell line: YAP1 overexpression is accompanied by an increase in reactive oxygen species generation [205] as atheroprotection [180,206]	←	according to an exhaustive review: promoted angiogenesis [201] can contribute to atherosclerotic plaque instability, which can increase cardio-cerebrovascular disease risks [202]	→
		rs1942812689:A	taactttagt	T A	ttggcgtttg	15.23±1.07 4.87±0.44	↑	20.76	10^{-6}	A						
		rs1942812689:C	taactttagt	T C	ttggcgtttg	15.23±1.07 13.51±0.95	↑	2.47	0.05	D						
		rs1213141882:T	cctaacttta	G T	ttttggcgtt	15.23±1.07 9.61±0.77	↑	8.87	10^{-6}	A						
		rs1472128107:T	ggcgcttctc	C T	taactttagt	15.23±1.07 7.72±0.62	↑	13.51	10^{-6}	A						

Table S3. (continued).

Gene		Candidate SNP marker				K_D, nM, MEAN ± SEM, *in silico*					Effect on human health during atherosclerosis, atherogenesis and atheroprotection [Ref]					
#	Entrez ID	dbSNP ID:min [36]	5′ flank, 10 bp	wt<br>min	3 flank, 10 bp	wt<br>min	Δ	Z	p	ϱ	Atherogenesis	ASg	Atheroprotection	ASp	Atherosclerosis	AS
16	YAP1 (10413)	rs1027307673:T	gggcgcttct	C<br>T	ctaactttag	15.23±1.07<br>7.87±0.55	↑	13.91	10^{6}	A	according to an exhaustive review: inhibited apoptosis [208] that can retard atherogenesis [209]	←	in human disease models using human colorectal cancer SW48 cell line: YAP1 overexpression is accompanied by an increase in reactive oxygen species generation [205] as atheroprotection [180,206]	←	according to an exhaustive review: promoted angiogenesis [201] can contribute to atherosclerotic plaque instability, which can increase cardio-cerebrovascular disease risks [202]	→
		rs1390734168:A	ggcgggcgct	T<br>A	ctcctaactt	15.23±1.07<br>13.64±0.95	↑	2.21	0.05	D						
		rs1943014282:C	ttgcccaaaa	G<br>C	ttaataggtt	8.28±0.83<br>6.25±0.56	↑	4.18	10^{3}	B						
		rs550925743:C	ctttgcccaa	A<br>C	agttaatagg	8.28±0.83<br>6.64±0.60	↑	3.40	10^{3}	B						
		rs550925743:T	ctttgcccaa	A<br>T	agttaatagg	8.28±0.83<br>6.38±0.45	↑	4.15	10^{3}	B						
		rs1943013634:G	actttgccca	A<br>G	aagttaatag	8.28±0.83<br>6.25±0.69	↑	3.81	10^{3}	B						
		rs1441432455:T	taaacttcag	C<br>T	ataggaactt	8.28±0.83<br>7.12±0.78	↑	2.02	0.05	D						
		rs1947929167:T	tcaggatgga	G<br>T	aaatttacta	9.05±0.81<br>7.56±0.60	↑	2.93	10^{2}	C						
		rs771074378:A	actcaggatg	G<br>A	agaaatttac	9.05±0.81<br>5.49±0.55	↑	7.27	10^{6}	A						
		rs111626988:T	gtgcgcgtcg	G<br>T	gggaggcaga	145.95±13.14<br>103.88±8.31	↑	5.76	10^{6}	A						
		rs1238665178:T	tcagggggtg	C<br>T	gcgtcggggg	145.95±13.14<br>97.83±8.80	↑	6.23	10^{6}	A						
		rs902415336:A	gcgtagccct	C<br>A	gctcgcctgg	145.95±13.14<br>80.10±7.21	↑	9.38	10^{6}	A						

Note: see the footnotes to Tables S1 and S2. *Alleles*: wt, ancestral; min, minor; deletion. K_D, dissociation constant of the TBP–DNA complex; Z and p, Fisher's Z-test and its statistical significance estimate; ρ, heuristic rank of candidate SNP markers from the "best" (A) to the "worst" (E).

Table S4. Comparison of the effects of unidirectional changes (a) in the expression of the human hub genes for atherogenesis, atheroprotection and atherosclerosis on the activity of these processes and (b) in the expression of the corresponding animal homologous genes that are differentially expressed (DEG) during domestication on the microevolutionary events leading to differences between domestic and wild animals.

#	Gene (NCBI Entrez ID)	(a) Humans: effects of changes in gene expression on human health [Refs]						(b) Animals: effects of changes in gene expression on the pattern of divergence between domestic animals and their wild conspecifics from their most recent ancestor								
		Atherogenesis (ASg)		Atheroprotection (ASp)		Atherosclerosis (AS)										
		deficit	excess	deficit	excess	deficit	excess	Domestic	Wild	Tissue	Homologous DEG	log_2	P_{ADG}	deficit	excess	[Ref]
1	*APOA1* (335)	ASg⟶ [116]	ASg⟶ [117]	ASp⟶ [116]	ASp⟵ [118]	AS⟶ [116]	AS⟵ [116]	guinea pig	cavy	frontal cortex	*Apoa1*	−3.21	10^{-2}	domestic	wild	[215]
2	*C1QTNF9* (338872)	ASg⟶ [124]	ASg⟵ [121]	ASp⟵ [123]	ASp⟵ [120]	AS⟶ [122]	AS⟵ [119]	guinea pig	cavy	frontal cortex	*C1qb*	1.26	10^{-4}	wild	domestic	[215]
								guinea pig	cavy	frontal cortex	*Col4a3*	1.84	10^{-11}	wild	domestic	[215]
								guinea pig	cavy	frontal cortex	*Col11a2*	−2.19	10^{-11}	domestic	wild	[215]
								guinea pig	cavy	frontal cortex	*Col9a1*	−0.94	0.05	domestic	wild	[215]
								guinea pig	cavy	frontal cortex	*Col9a2*	0.61	0.05	wild	domestic	[215]
								guinea pig	cavy	frontal cortex	*Col6a2*	0.74	0.05	wild	domestic	[215]
								guinea pig	cavy	frontal cortex	*Col24a1*	1.35	10^{-9}	wild	domestic	[215]
								tame rat	aggressive rat	midbrain tegmentum	*Col14a1*	1.90	0.05	wild	domestic	[213]
								domectic rabbit	wild rabbit	parietal-temporal cortex	*Col6a3*	1.45	10^{-4}	wild	domestic	[216]
								domectic rabbit	wild rabbit	parietal-temporal cortex	*Col8a1*	1.24	10^{-3}	wild	domestic	[216]
								domectic rabbit	wild rabbit	hippocampus	*Col28a1*	−2.69	0.05	domestic	wild	[216]
								domectic chicken	wild hicken	pituarity	*C1qtnf4*	1.01	10^{-4}	wild	domestic	[221]
								domectic chicken	wild hicken	pituarity	*Col28a1*	−0.95	10^{-2}	domestic	wild	[221]
								domectic chicken	wild hicken	pituarity	*Col4a4*	−0.79	10^{-7}	domestic	wild	[221]
								domectic chicken	wild hicken	pituarity	*Col8a2*	0.72	10^{-7}	wild	domestic	[221]
								domectic chicken	wild hicken	pituarity	*Col14a1*	−0.65	10^{-4}	domestic	wild	[221]
3	*KLF2* (10365)	ASg⟶ [148]	ASg⟵ [146]	ASp⟵ [149]	ASp⟵ [145]	AS⟶ [147]	AS⟵ [150]	tame fox	aggressive fox	frontal cortex	*Klf12*	−0.31	0.05	domestic	wild	[218]
								dog	wolf	blood	*Klf1*	−4.78	10^{-5}	domestic	wild	[217]
4	*LCAT* (3931)	ASg⟶ [152]	ASg⟵ [155]	ASp⟶ [156]	ASp⟵ [151]	AS⟶ [153]	AS⟵ [154]	guinea pig	cavy	frontal cortex	*Lcat*	−1.14	0.05	domestic	wild	[215]
5	*SERPINF1* (5176)	ASg⟵ [187]	ASg⟶ [185]	ASp⟵ [186]	ASp⟶ [183, 184]	AS⟶ [188]	AS⟵ [189, 190, 191]	guinea pig	cavy	frontal cortex	*Serpinf1*	−1.88	10^{-6}	domestic	wild	[215]
								domestic rabbit	wild rabbit	hypothalamus	*Serpinf1*	−2.01	10^{-2}	domestic	wild	[216]
6	*TLR2* (7097)	ASg⟵ [199]	ASg⟵ [195]	ASp⟵ [197]	ASp⟵ [198]	AS⟶ [196]	AS⟶ [200]	guinea pig	cavy	frontal cortex	*Tlr3*	1.39	0.05	wild	domestic	[215]
								guinea pig	cavy	frontal cortex	*Tlr2*	−1.22	0.05	domestic	wild	[215]
								domestic chicken	wild chicken	pituarity	*Tlr1a*	0.51	10^{-4}	wild	domestic	[221]
								domestic chicken	wild chicken	pituarity	*Tlr21*	0.59	10^{-8}	wild	domestic	[221]

Note: see the footnotes to Tables S1, S2, and S3. log_2: the log_2-transformed fold change (i.e. the ratio of tame to aggressive rats' expression levels of a given gene); P_{ADJ}: statistical significance according to Fisher's Z-test with the Benjamini correction for multiple comparisons.

© 2023 by the authors. Submitted for possible open access publication under the terms and conditions of the Creative Commons Attribution (CC BY) license (http://creativecommons.org/licenses/by/4.0/).
